# The endocarp evolution of Cissampelideae (Menispermaceae): integrating extant and fossil species

**DOI:** 10.1093/aob/mcaf240

**Published:** 2025-10-22

**Authors:** Lian Lian, Qiang Zhang, Wei Wang

**Affiliations:** State Key Laboratory of Plant Diversity and Specialty Crops, Institute of Botany, Chinese Academy of Sciences, Beijing 100093, China; China National Botanical Garden, Beijing 100093, China; Guangxi Key Laboratory of Plant Conservation and Restoration Ecology in Karst Terrain, Guangxi Institute of Botany, Guangxi Zhuang Autonomous Region and Chinese Academy of Sciences, Guilin 541006, China; State Key Laboratory of Plant Diversity and Specialty Crops, Institute of Botany, Chinese Academy of Sciences, Beijing 100093, China; China National Botanical Garden, Beijing 100093, China; University of Chinese Academy of Sciences, Beijing 100049, China

**Keywords:** Character evolution, Cissampelideae, fossil, Menispermaceae, morphology, phylogeny

## Abstract

**Background and Aims:**

The integration of extant and extinct species is essential to understand the character evolution of organisms. However, the effect of incorporating fossil information on inferring evolutionary patterns of morphological characters is still poorly understood. Here, we used Cissampelideae, a tribe of pantropical lianas with rich fossil records, to provide new insights into the role of fossils in the inference of character evolution.

**Methods:**

Based on seven DNA regions and 24 endocarp characters, we reconstructed a dated phylogeny for 39 extant and 11 fossil species of Cissampelideae. For comparison, we also reconstructed a dated phylogeny for only extant species. Within the dated phylogenetic frameworks, we then reconstructed the evolutionary patterns of endocarp characters in the tribe.

**Key Results:**

Ancestral-state inferences of 6 of the 24 endocarp characters are changed when fossils are integrated. The endocarp of the most recent common ancestor of Cissampelideae had unbroken short raised transverse ridges lower than the unspiny dorsal crest. Within Cissampelideae, the broken transverse ridge is derived from the unbroken transverse ridge, the columnar protuberance is derived from the long raised transverse ridge, and the long raised transverse ridge is derived from the short raised transverse ridge.

**Conclusions:**

Endocarp transverse ridges of Cissampelideae show evolutionary trends of becoming fragmented and more elevated, which may be related to adaptation to limestone karst environments. This study highlights that fossil species have played important roles in recording morphological character evolution and showcases the necessity of integrating fossil and extant taxa to improve the inference of character evolution.

## INTRODUCTION

Understanding how morphological characters evolved through time is a fundamental question in evolutionary biology (Futuyma, 1979). The emergence of phylogenies underpins our ability to understand the evolutionary history of morphological characters in different organisms. In recent decades an explosion of studies has reconstructed past evolutionary events using data from living taxa (e.g. Guzmán and Vargas, 2005; Wang and Chen, 2007; Figueroa *et al.*, 2008; Jacobs *et al.*, 2009). However, living taxa only represent a small part of evolutionary history and more than 99 % of all species that ever existed are now extinct (Jablonski, 2004); especially older lineages often have higher extinction rates (Marshall, 2017). Data from only extant taxa might not be enough to faithfully recover events that occurred in the distant past, as trees based only on living species are lacking the necessary information to distinguish between alternative hypotheses (Louca and Pennell, 2020) and may even induce incorrect conclusions (Slater, 2013). The fossil record is our only resource for understanding the evolutionary patterns and processes of plant groups that have occurred in deep time. Fossils of each plant taxon, whether with or without plesiomorphy, can provide additional data related to their evolutionary history of morphological characters (Ksepka *et al.*, 2017; Mao *et al.*, 2021; Wright *et al.*, 2021). However, only a few simulation studies have been devoted to investigating the effect of incorporating fossil information on evolutionary inferences of morphological characters (Hermsen and Hendricks, 2008; Slater *et al.*, 2012; Gearty *et al.*, 2023), and empirical studies are still lacking.

Besides morphological characters, the stratigraphic sequence of fossil taxa in formations is also a potential source of phylogenetic reconstruction (Fisher, 1980). Although there has been debate about incorporating stratigraphic data into phylogenetic analysis (Smith, 2000; Alroy, 2002; Fisher, 2008), it has been proved by both simulation and empirical studies that stratigraphic information from fossils has a positive impact on phylogenetic inference (King *et al.*, 2017; Turner *et al.*, 2017; King, 2021; Mongiardino Koch *et al.*, 2021). The development of the Bayesian tip-dating method (also known as total-evidence dating; Ronquist *et al.*, 2012) makes it possible to infer the evolutionary patterns of morphological characters in a dated-phylogenetic framework (O’Reilly *et al.*, 2015). This method can integrate morphological data and stratigraphic information from the fossil record (Gavryushkina *et al.*, 2017) as well as molecular and morphological data from living organisms, and has been widely used in morphological character evolution analyses (Mao *et al.*, 2021; Wright *et al.*, 2021). For most studies, evolutionary inferences of morphological characters were only discussed based on the Bayesian tip-dating tree. To better understand the evolutionary history of morphological characters, further reconstructions of ancestral character states are needed.

Menispermaceae is a pantropical liana family and commonly known as the moonseed family because its endocarp is often curved in a crescent shape. Endocarps have long been considered the most important characters for the classification of the family (Diels, 1910). The bony or woody endocarps of Menispermaceae are easily preserved and fossilized in formations due to their durable and lignified structure (Jacques, 2009*a*). Cissampelideae is one of the most diverse tribes in this family, including six genera and ∼126 species (Ortiz *et al.*, 2016; Lian *et al.*, 2024). Chesters (1957) first reported two endocarp fossils of the tribe from the Miocene of Kenya, and the oldest confirmed fossil endocarp assignable to Cissampelideae is from the Early Danian of the Palaeocene (Jud *et al.*, 2018). Importantly, the vast majority of moonseed endocarp fossils that have been reported in the past decade were attributed to this tribe (Lian *et al.*, 2024). Based on combined morphological and molecular data, Lian *et al.* (2024) discovered that three fossil species (*Stephania jacquesii*, *S. auriformis* and *S. ornamenta*) that were previously assigned to Cissampelideae belong in Anomospermeae. After excluding the wrongly assigned fossils, Cissampelideae contains 11 endocarp fossil species (Table 1). There are a large number of detailed morphological descriptions for endocarps of extant and fossil species (Jacques, 2009*b*; Herrera *et al.*, 2011; Wefferling *et al.*, 2013; Ortiz *et al.*, 2016). Among the members of the tribe, the endocarps possess extensive morphological variations, such as the number of transverse ridges (varying from <10 to >20), an ornamental type of transverse ridges (short raised or long raised), perforation (presence or absence), chamber (presence or absence) and number of dorsal crests (one or two). Thus, Cissampelideae is an ideal group to integrate extant and fossil species to investigate the evolutionary inference of morphological characters. Moreover, species with different ornamentation on Cissampelideae endocarps tend to live in different habitats. For example, *Stephania* species that have endocarps with columnar protuberances grow exclusively on limestone karst (Luo, 1982), while *Botryodiscia* with relatively smooth endocarps are distributed in roadside shrubland (Luo, 1982; Luo *et al.*, 2008). Thus, investigating endocarp evolution can also contribute to improvement of our understanding of the adaptive evolution of Cissampelideae.

**Table 1.** Fossil taxa of endocarps assigned to Cissampelideae.

Fossil taxon	Epoch	Age minimum (Ma)	Age maximum (Ma)	Locality	Reference
*Cissampelos defranceschii*	Middle Eocene	37.71	48.07	Xizang, China	Del Rio *et al.* (2021)
*Cissampelos rusingensis*	Burdigalian (Miocene)	15.98	20.45	Nyanza, Kenya	Chesters (1957)
*Palaeoluna bogotensis*	Middle–Late Paleocene	56.00	61.66	Cundinamarca, Colombia	Herrera *et al.* (2011)
*Stephania bangorensis*	Middle Eocene	37.71	48.07	Xizang, China	Del Rio *et al.* (2021)
*Stephania geniculata*	Middle Paleocene	59.24	61.66	Guangdong, China	Han *et al.* (2020)
*Stephania hootae*	Middle Eocene	37.71	48.07	Hessen, Germany	Collinson et al. (2012)
*Stephania miocenica*	Burdigalian (Miocene)	15.98	20.45	Nyanza, Kenya	Chesters (1957)
*Stephania palaeosudamericana*	Middle–Late Paleocene	56.00	61.66	Guajira, Colombia	Herrera *et al.* (2011)
*Stephania psittaca*	Early Danian (Paleocene)	61.66	66.00	Chubut, Argentina	Jud *et al.* (2018)
*Stephania shuangxingii*	Middle Eocene	37.71	48.07	Xizang, China	Del Rio *et al.* (2021)
*Stephania wilfii*	Paleocene–Eocene boundary to Middle Eocene	37.71	56.00	Wyoming, USA; Xizang, China	Han *et al.* (2018) and Del Rio *et al.* (2021)

**Fig. 1.**
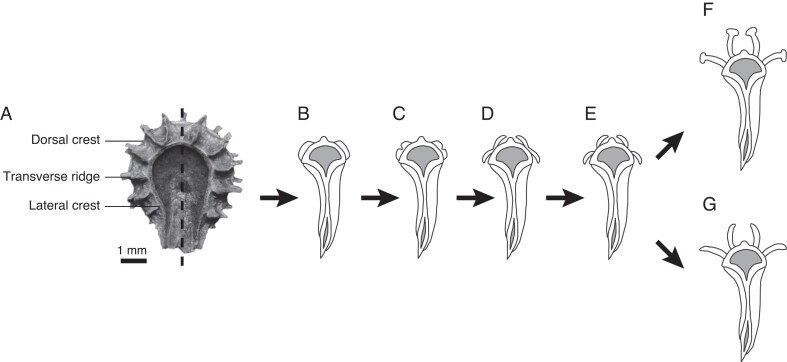
Cutaway diagram of the evolutionary pattern of surface ornamentation on endocarps in *Stephania*. (A) *Stephania longa* endocarp showing the terminology used in this study. The dashed line indicates the cut position. (B) Short raised transverse ridges. (C) Broken short raised transverse ridges. (D) Columnar protuberances with base connection. (E) Columnar protuberances with base separation. (F) Columnar protuberances with tubercle-like apex. (G) Columnar protuberances with hook-like apex.

As the largest genus within Menispermaceae, *Stephania* exhibits a significant amount of intrageneric variation in endocarp characters (Diels, 1910; Jacques, 2009*b*; Ortiz *et al.*, 2016). Based on the Chinese *Stephania* (including *Botryodiscia* as *Stephania* subg. *Botryodiscia*), Luo (1982) proposed the hypothesis that the collumnotype ornamentation (corresponding to columnar protuberances in this study) on endocarps in *Stephania* evolved from costulotype (corresponding to short raised transverse ridges in this study) (Fig. 1). Based on this hypothesis, the short raised transverse ridges are broken in the middle and protrusion extends at both ends, leading to the formation of columnar protuberances (Fig. 1). This hypothesis has never been tested in a phylogenetic framework. In particular, nine ancient endocarp fossils with unbroken transverse ridges were reported from *Stephania* (Han *et al.*, 2018, 2020; Jud *et al.*, 2018; Del Rio *et al.*, 2021), of which six have been confirmed to be stem members of Cissampelideae (Lian *et al.*, 2024). To better understand the evolutionary history of endocarps in this tribe, one needs to infer the evolutionary patterns of endocarps in a tribe-wide phylogenetic framework by integrating extant and fossil species.

In this study, we generated a dated phylogeny for Cissampelideae based on the combined molecular and morphological dataset with 39 extant and 11 fossil species. For comparison, evolutionary inferences of endocarp characters in Cissampelideae with and without fossil taxa were performed. Our aims were (1) to evaluate the effect of incorporating fossil information for the inference of character evolution; and (2) to investigate the evolutionary history of the endocarp of Cissampelideae and its potential adaptation to karst habitats.

## MATERIALS AND METHODS

### Molecular data

We sampled 39 taxa, representing all six of the currently recognized genera of Cissampelideae (Supplementary Data Table S1). Thirty additional species of Menispermoideae were selected as outgroups, representing all the other seven tribes of the subfamily (Lian *et al.*, 2020). Our taxon sampling covers the diversity of endocarps in Cissampelideae, as well as in Menispermoideae. Menispermeae were used to root the tree based on our previous studies (Wang *et al.*, 2012; Ortiz *et al.*, 2016). Seven DNA regions, including five plastid (*rbc*L, *atp*B, *mat*K, *ndh*F, *trn*L-F) and two nuclear (26S rDNA, ITS) loci, were used in this study. Genomic DNA extraction, amplification and sequencing procedures followed Wang *et al.* (2012, 2016). DNA sequences were assembled, edited and aligned using Geneious Prime 2025.1.2 (Kearse *et al.*, 2012). Voucher information and GenBank accession numbers are listed in Supplementary Data Table S1.

### Morphological data

Based on our previous study (Lian *et al.*, 2024), a total of 11 endocarp fossils assigned to Cissampelideae were included (Table 1). Information on morphological features was obtained from the literature and personal observations, particularly Herrera *et al.* (2011), Jacques *et al.* (2011), Wefferling *et al.* (2013), Ortiz *et al.* (2016) and Lian *et al.* (2024) (Supplementary Data Table S2). Finally, we obtained 17 endocarp characters for subsequent analyses. The morphological character matrix containing 53 fossil and living species is provided in Supplementary Data Table S3.

### Phylogenetic analyses and divergence time estimation

DNA sequences were assembled, aligned and adjusted manually in Geneious v.10.1.3 (Kearse *et al.*, 2012). The final alignments contained 69 living taxa with 1386 bp for *rbc*L, 1407 bp for *atp*B, 1251 bp for *mat*K, 2073 bp for *ndh*F, 1127 bp for *trn*L-F (excluding two poly regions with 17 nucleotides), 1355 bp for 26S rDNA and 601 bp for ITS. The seven-locus dataset is provided in the Supplementary data (File S1). Our previous study indicated that relationships within Cissampelideae recovered by these five plastid DNA and two nuclear DNA datasets are not significantly incongruent (Lian *et al.*, 2024). Here, we combined the five plastid and two nuclear DNA regions for subsequent analyses.

Based on the combined molecular and morphological dataset, phylogeny and divergence times were co-estimated using the Bayesian inference method with a Bayesian uncorrelated log-normal clock model (Drummond *et al.*, 2006), as implemented in BEAST v.2.7.7 (Bouckaert *et al.*, 2014). Bayesian phylogenetic tip-dating analysis was performed under a fossilized birth–death prior process (Heath *et al.*, 2014). For molecular data, the GTR model with a γ distribution was used for each DNA locus partition separately. For morphological data, the Mk model with an equal rate distribution (Lewis, 2001) was used. The calibrated ages of the fossils were assigned a uniform prior distribution with the confidence interval of the deposition time of the fossil, defined as the interval from the maximum to the minimum possible age of the stratum in which the fossil was found. We used the geological time scale of Cohen *et al.* (2013, updated). To avoid overestimation of root age, we set the maximum age of the root at 100 Ma, which is the crown group age of the Menispermoideae (Wang *et al.*, 2012), with a normal distribution and standard deviation of 3. Markov chain Monte Carlo (MCMC) chains were run for 50 million generations, sampling every 1000 generations. Convergence and the adequate effective sample size values (>200) were checked in Tracer v.1.7 (Rambaut *et al.*, 2018). After the initial 25 % of trees had been discarded as burn-in, the maximum clade credibility (MCC) tree with median branch lengths and 95 % highest posterior density (HPD) intervals on nodes was then built using TreeAnnotator v.2.1.3 (part of the BEAST package).

For comparative purposes, we also performed node-dating analysis only based on the molecular data for 69 extant taxa. Phylogeny and divergence times were co-estimated using the Bayesian inference method in BEAST v.2.7.7 (Bouckaert *et al.*, 2014). Eight fossils that can be confidently placed in our tree were selected as calibration points (Supplementary Data Table S4) and used with a uniform prior distribution (hard-bound constraint; Ho and Phillips, 2009; Parham *et al.*, 2012). The setting of the maximum age of the root was the same with the tip-dating analysis. Two independent runs, each consisting of four MCMC chains, were conducted with one tree sampled for every 1000 generations over 50 million generations, starting with a random tree. Stationarity was determined in Tracer v.1.7 (Rambaut *et al.*, 2018). A majority-rule (>50 %) consensus tree was constructed after removing the burn-in period samples (the first 25 % of sampled trees). Phylogenetic analyses were also conducted using the maximum likelihood (ML) method in RAxML v.8.0 (Stamatakis, 2014). For ML analysis, RAxML was performed with the GTR + I + Γ substitution model for each DNA region, and the fast bootstrap option with 1000 replicates.

### Evolutionary reconstructions of endocarp characters

Patterns of character evolution were inferred using the MCC trees generated from the tip-dating analyses with the ML approach as implemented in Mesquite v.3.81 (Maddison and Maddison, 2023). To assess the effect of including fossil species on inferring evolutionary patterns of characters, we also ran analyses using the MCC tree generated from the node-dating analysis. The Markov *k*-state one-parameter model of evolution for discrete unordered characters (Lewis, 2001) was used.

## RESULTS

### Phylogeny of Cissampelideae

**Fig. 2.**
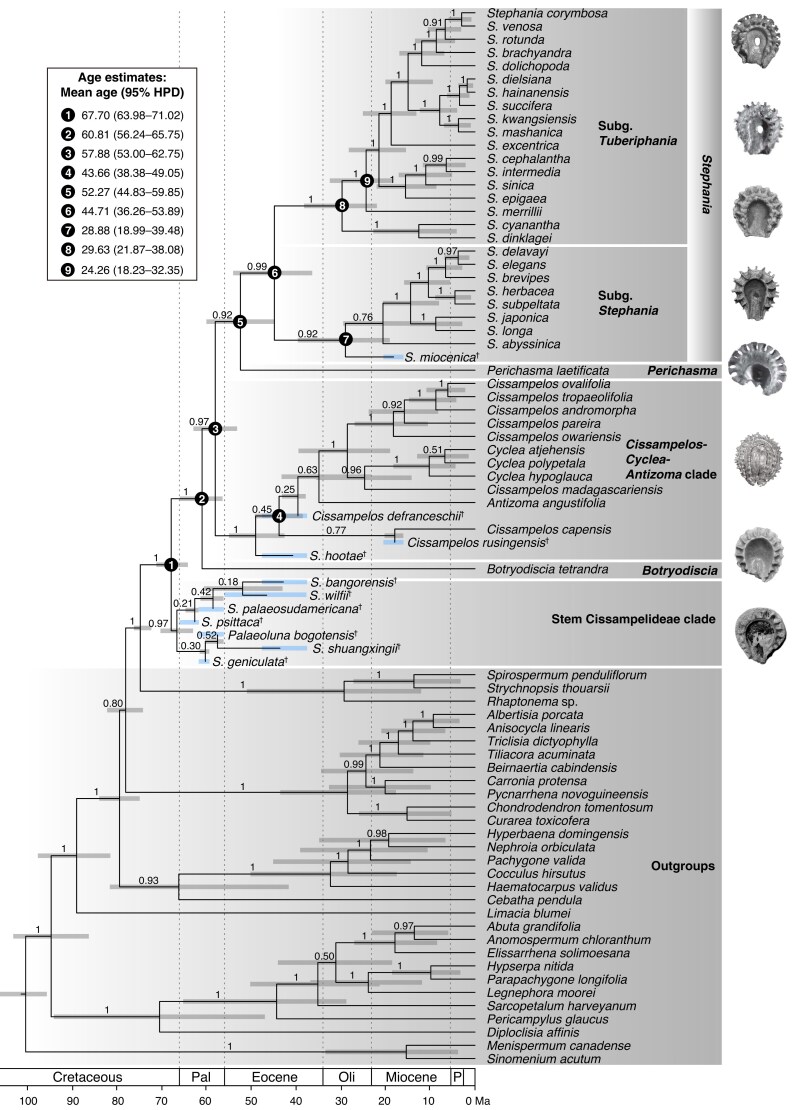
Chronogram of Cissampelideae with extant and extinct taxa using tip-dating in BEAST. Numbers above branches are posterior probabilities. Grey node bars indicate 95 % HPD intervals. Blue bars represent uncertainty in the fossil ages. Taxa with ‘^†^’ are fossil species. Nodes of interests are marked as 1–9. Representative endocarps of Cissampelideae are shown on the right (from the top: *Stephania brachyandra*, *Stephania rotunda*, *Stephania cephalantha*, *Stephania longa*, *Perichasma laetificata*, *Cyclea hypoglauca*, *Botryodiscia tetrandra* and *Stephania psittaca*^†^). Pal, Palocene; Oli, Oligocene; P, Pliocene.

The topology of extant and fossil Cissampelideae phylogeny generated based on the combined molecular and morphological dataset is shown in Fig. 2. The monophyly of Cissampelideae is strongly supported (posterior probability (PP) = 1.00). Within Cissampelideae, a clade consists of seven fossil species (indicated with ‘^†^’ in the text) of *Stephania* and *Palaeoluna* (i.e. *S*. *psittaca*^†^, *S*. *geniculata*^†^, *S*. *palaeosudamericana*^†^, *S*. *shuangxingii*^†^, *S*. *wilfii*^†^, *S*. *bangorensis*^†^ and *P*. *bogotensis*^†^) is the first-branching lineage (referred to hereafter as the stem Cissampelideae clade; PP = 1.00), followed by *Botryodiscia* (PP = 0.97), then the clade contains three fossil species (*C*. *defranceschii*^†^, *C*. *rusingensis*^†^ and *S*. *hootae*^†^) and extant *Cissampelos*, *Cyclea* and *Antizoma* (*Cissampelos–Cyclea–Antizoma* clade; PP = 0.92). *Perichasma* is sister to *Stephania* (PP = 0.99). *Stephania* consists of *S.* subg. *Stephania* (PP = 0.92) and *S.* subg. *Tuberiphania* (PP = 1.00), both which are strongly supported as monophyletic.

For extant Cissampelideae, the Bayesian inference and ML analyses resulted in identical trees (Supplementary Data Fig. S1). The topology of living Cissampelideae phylogeny (Supplementary Data Fig. S1) is highly congruent with the extant and fossil Cissampelideae phylogeny (Fig. 2) except for fossil insertion.

### Divergence time estimation

Divergence time estimates for extant and fossil Cissampelideae using tip-dating are shown in Fig. 2. The crown group age of Cissampelideae was estimated at 67.70 Ma (95 % HPD 63.98–71.02; node 1). The extant Cissampelideae dated back to 60.81 Ma (95 % HPD 56.24–65.75; node 2). The split of the *Cissampelos–Cyclea–Antizoma* clade and its sister group occurred at 57.78 Ma (95 % HPD 53.00–62.75; node 3). The crown group age of the extant *Cissampelos–Cyclea–Antizoma* clade was estimated at 43.66 Ma (95 % HPD 38.38–49.05; node 4). The split of *Perichasma* and *Stephania* was estimated at 52.27 Ma (95 % HPD 44.83–59.85; node 5). The split of *S.* subg. *Stephania* and *S.* subg. *Tuberiphania* was at 44.71 Ma (95 % HPD 36.26–53.89; node 6). The crown group ages of *S.* subg. *Stephania* and *S.* subg. *Tuberiphania* were estimated at 28.88 Ma (95 % HPD 18.99–39.48; node 7) and 29.63 Ma (95 % HPD 21.87–38.08; node 8), respectively.

Divergence time estimates for extant Cissampelideae using node-dating are shown in Supplementary Data Fig. S1. The crown group age of extant Cissampelideae was estimated at 57.18 Ma (95 % HPD 48.14–67.16; node 1). The split of the *Cissampelos–Cyclea–Antizoma* clade and its sister group occurred at 53.60 Ma (95 % HPD 45.00–63.36; node 2). The crown group age of the *Cissampelos–Cyclea–Antizoma* clade was estimated at 35.39 Ma (95 % HPD 25.62–44.95; node 3). The split of *Perichasma* and *Stephania* occurred at 49.54 Ma (95 % HPD 41.10–59.49; node 4). The split of *S.* subg. *Stephania* and *S.* subg. *Tuberiphania* was at 44.27 Ma (95 % HPD 34.77–53.53; node 5). The crown group ages of *S.* subg. *Stephania* and *S.* subg. *Tuberiphania* were estimated at 22.84 Ma (95 % HPD 14.61–31.55; node 6) and 30.40 Ma (95 % HPD 23.18–38.06; node 7), respectively. The crown group age of Asian *S.* subg. *Tuberiphania* was estimated at 24.26 Ma (95 % HPD 18.23–32.35; node 8).

### Endocarp evolution of Cissampelideae

**Fig. 3.**
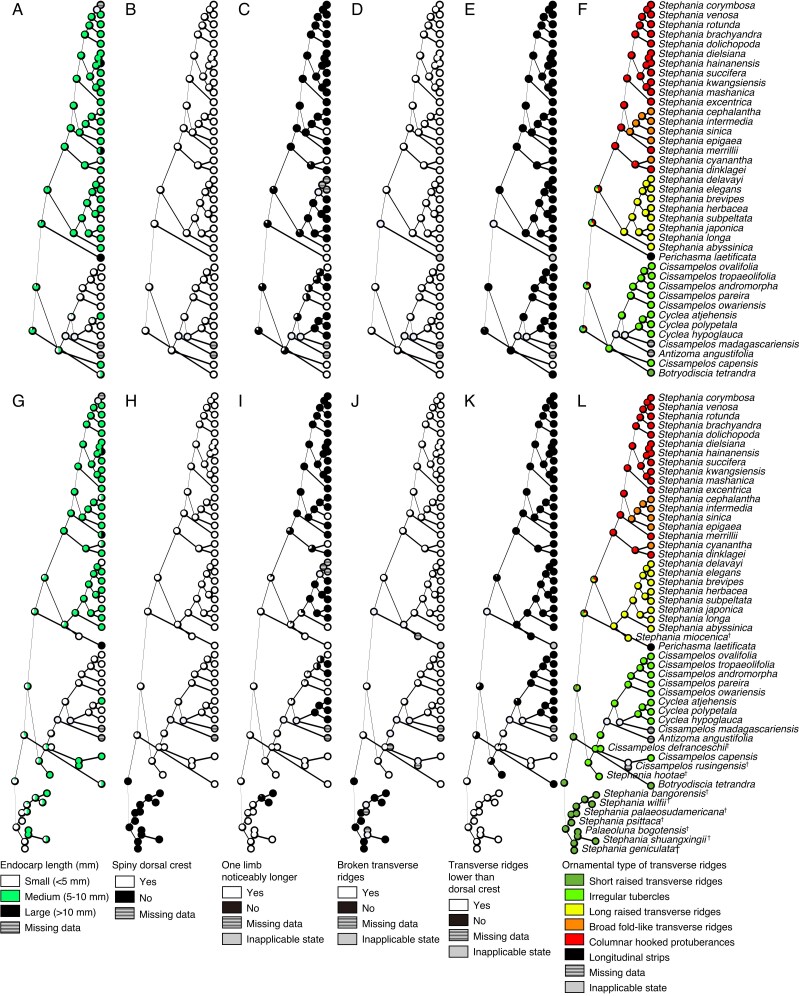
Comparison of likelihood inference of character evolution in Cissampelideae using Mesquite with and without fossil taxa. (A–F) Only extant taxa. (G–L) Extant and fossil taxa. (A, G) Endocarp length. (B, H) Spiny dorsal crest. (C, I) One limb noticeably longer. (D, J) Broken transverse ridges. (E, K) Transverse ridges lower than dorsal crest. (F, L) Ornamental type of transverse ridges.

**Fig. 4.**
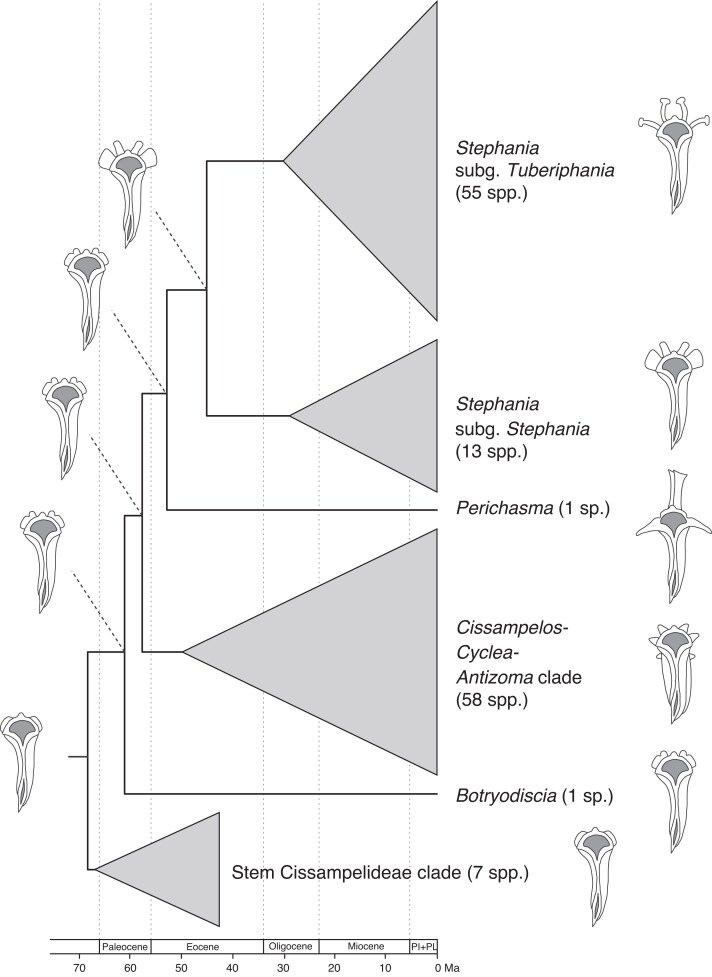
Endocarp evolution in Cissampelideae. Topology is modified from Fig. 2. Pl, Pliocene; PL, Pleistocene.

Likelihood inferences of evolution of endocarp characters in Cissampelideae with and without fossil taxa are shown in Supplementary Data Figs S2 and S3, respectively. Six of the 24 endocarp characters’ ancestral-state inferences were different when fossils were integrated, i.e. endocarp length, spiny dorsal crest or not, one limb noticeably longer or not, broken transverse ridges or not, transverse ridges lower than dorsal crest or not, and ornamental type of transverse ridges (Fig. 3). For example, medium size (5–10 mm) endocarp was inferred to be the ancestral state of Cissampelideae based on extant species (Fig. 3A), while small size (<5 mm) endocarp was inferred to be the ancestral state of Cissampelideae based on extant and fossil species (Fig. 3G). Based on the integration of inferences of 24 characters, the putative endocarp of ancestral Cissampelideae had unbroken short raised transverse ridges lower than the unspiny dorsal crest (Fig. 4).

## DISCUSSION

### The effect of incorporating fossils on evolutionary inferences of morphological characters

The intertribal relationships within Menispermoideae recovered here by integrating extant and fossil taxa are highly congruent with the results of previous studies (Wang *et al.*, 2012; Ortiz *et al.*, 2016). All nodes, especially related to fossils, in the tree are resolved, supporting the positive impact of stratigraphic information from fossils on tree topology (Mongiardino Koch *et al.*, 2021). Our previous study has also indicated that including stratigraphic information may greatly increase the proportion of resolved nodes, especially nodes involving fossil taxa (Lian *et al.*, 2024). Within Cissampelideae, our phylogenetic analyses support the stem Cissampelideae clade as the earliest diverging lineage, which coincides with our previous study (Lian *et al.*, 2024). Within extant Cissampelideae, the intergeneric relationships recovered here are in agreement with the results derived from our previous studies (Wang *et al.*, 2012; Ortiz *et al.*, 2016; Lian *et al.*, 2020, 2024). Our tip-dating analysis suggests a crown group age of 67.70 Ma (95 % HPD 63.98–71.02) for Cissampelideae, which is consistent with the results of our previous study (Lian *et al.*, 2024; 63–74 Ma). Tip-dating analysis and node-dating analysis suggest the crown ages of 60.81 Ma (95 % HPD 56.24–65.75) and 57.18 Ma (95 % HPD 48.14–67.16) for the extant Cissampelideae, which are highly congruent with those of our previous study (Lian *et al.*, 2024; 48–64 Ma).

Without the inclusion of fossil taxa, evolutionary inferences of endocarp characters indicate that the broken irregular tubercle higher than the dorsal crest is the ancestral state in Cissampelideae, and the long raised transverse ridges and columnar protuberances are derived from the irregular tubercle (Fig. 3). Based on the likelihood inferences integrating extant and fossil taxa (Fig. 3), we discover that the ancestor of Cissampelideae might possess endocarp with unbroken transverse ridges that are lower than the dorsal crest. The fossil species, especially the stem Cissampelideae clade, recorded some primitive states that are lost in extant species, such as unbroken short raised transverse ridges, transverse ridges lower than the unspiny dorsal crest, and one limb noticeably longer, which are key for the evolutionary inference of endocarps in Cissampelideae (Fig. 3). Additionally, in Menispermoideae, medium size (5–10 mm) endocarp was inferred to be the ancestral state based on extant species (Fig. 3A), while small size (<5 mm) endocarp was inferred to be the ancestral state based on extant and fossil species (Fig. 3G). These results suggest that the integration of fossil and extant taxa is essential to infer accurately the evolutionary patterns of characters rather than only considering extant taxa, as in other studies (e.g. Finarelli and Flynn, 2006; Jones *et al.*, 2018; Mao *et al.*, 2021).

### Evolution of endocarps in Cissampelideae

By integrating fossils and extant taxa, we inferred the endocarp evolution of Cissampelideae (Fig. 4). Endocarp characters, including number of dorsal crests, perforation, chamber and ornament type of transverse ridges, have traditionally been used for generic recognition within Cissampelideae, especially for the delimitation of *Stephania*. These characters are reconstructed on the dated phylogeny with the extant and fossil species (Supplementary Data Fig. S2). Likelihood inferences indicate evolutionary patterns of endocarp morphological characters (Fig. 3). The endocarp of the ancestor of Cissampelideae had unbroken transverse ridges lower than the dorsal crest, whereas broken transverse ridges higher than the dorsal crest evolved in the middle Palaeocene (Fig. 4). With the emergence of broken transverse ridges, multiple types of transverse ridges developed from the broken short transverse ridges; for example, *Botryodiscia* evolved short raised transverse ridges, the *Cissampelos–Cyclea–Antizoma* clade evolved irregular tubercles, *Perichasma* evolved longitudinal strips, and *Stephania* evolved long raised transverse ridges. In *Stephania*, *S.* subg. *Stephania* kept the long raised transverse ridges and *S.* subg. *Tuberiphania* evolved columnar hooked protuberances. In Cissampelideae, columnar protuberances were hypothesized to evolve from the broken transverse ridges (Luo, 1982). Based on this hypothesis, the short raised transverse ridges were broken in the middle and protrusion extended at both ends, leading to the formation of columnar protuberances (Fig. 1). However, all endocarps of living Cissampelideae have varying degrees of broken transverse ridges (Jacques, 2009*b*). Our results suggest that the broken transverse ridges are derived from the unbroken transverse ridges, columnar protuberances are derived from the long raised transverse ridges, and the long raised transverse ridges are derived from the short raised transverse ridges.

The species of *S.* subg. *Tuberiphania* with columnar protuberances tend to live in limestone karst habitats (Luo, 1982), whereas other species in Cissampelideae with relatively smooth endocarps are distributed in non-karst habitats (Diels, 1910; Luo *et al.*, 2008). Considering that a tubercle-like or hook-like apex occurs at the end of the columnar protrusion, the columnar protuberances on the endocarps of *S.* subg. *Tuberiphania* may be a fixed device, hence facilitating adaptation to limestone karst habitats (Luo *et al.*, 2008). The Asian *S.* subg. *Tuberiphania* was estimated to start diversifying in the Late Oligocene (∼24 Ma; node 9 in Fig. 2). Around the Oligocene–Miocene boundary (∼23 Ma), the deformation and uplift of the Qinghai–Tibet Plateau and the eastward lateral extrusion of Indochina took place (Ding *et al.*, 2017; Deng *et al.*, 2021; Li *et al.*, 2024). Along with the orogeny, the eastward throughflow of the Yangtze River system occurred at the same time (Zheng, 2015). Meanwhile, the East Asian summer monsoon started to become established (∼25 Ma; Ding *et al.*, 2021) and the South Asian summer monsoon intensity increased (24–23 Ma; Clift and Webb, 2019), which brought high precipitation. The limestone karst is primarily made up of calcium carbonate, which easily dissolves in freshwater, and high precipitation and river incision accelerated the dissolution of limestone. These geoclimatic events might have promoted the formation of insular karst habitats and thus provided new ecological opportunities for *S.* subg. *Tuberiphania*. This hypothesis needs to be tested by evaluating trait–environment associations in a phylogenetic framework with dense taxon sampling in *Stephania*.

### Conclusions

We present a tip-dated phylogenetic analysis of Cissampelideae based on combined molecular and morphological data with extant and fossil species and reveal endocarp evolution in this tribe. The endocarp of the ancestor of Cissampelideae had unbroken short raised transverse ridges lower than an unspiny dorsal crest. Within Cissampelideae, the broken transverse ridges are derived from unbroken transverse ridges, columnar protuberances are derived from long raised transverse ridges, and long raised transverse ridges are derived from short raised transverse ridges, which may be related to adaptation to karst habitats. This study highlights the key role of fossils in evolutionary inferences about morphological characters.

## Supplementary Material

mcaf240_Supplementary_Data

## SUPPLEMENTARY DATA

Supplementary data are available at Annals of Botany online and consist of the following. Table S1: taxa, vouchers and GenBank accession numbers for the sequences used in this study. Table S2: endocarp characters and character states used in this study. Table S3: data matrix of 24 endocarp characters. Table S4: calibrating fossils used for node-dating. Figure S1: chronogram of Cissampelideae inferred from the seven-locus dataset using node-dating in BEAST. Figure S2: likelihood inference of evolution of endocarp characters in Cissampelideae with extant and extinct taxa using Mesquite. Figure S3: likelihood inference of evolution of endocarp characters in Cissampelideae with extant taxa using Mesquite. File S1: combined data matrix in Nexus format.

## References

[mcaf240-B1] Alroy J . 2002. Stratigraphy in phylogeny reconstruction—reply to Smith (2000). Journal of Paleontology 76: 587–589. doi:10.1666/0022-3360(2002)076<0587:SIPRRT>2.0.CO;2

[mcaf240-B2] Bouckaert R , Heled J, Kühnert D, et al 2014. BEAST 2: a software platform for Bayesian evolutionary analysis. PLoS Computational Biology 10: e1003537. doi:10.1371/journal.pcbi.1003537 24722319 PMC3985171

[mcaf240-B3] Chesters K . 1957. The Miocene flora of Rusinga Island, Lake Victoria, Kenya. Palaeontographica Abteilung B 101: 30–71.

[mcaf240-B4] Clift PD , Webb AAG. 2019. A history of the Asian monsoon and its interactions with solid earth tectonics in Cenozoic South Asia. In: Treloar PJ, Searle MP. eds. Himalayan tectonics: a modern synthesis. London: Geological Society, 631–652.

[mcaf240-B5] Cohen KM , Finney SC, Gibbard PL, Fan JX. 2013; updated. The ICS International Chronostratigraphic Chart. Episodes 36: 199–204. doi:10.18814/epiiugs/2013/v36i3/002

[mcaf240-B6] Collinson ME , Manchester SR, Wilde V. 2012. Fossil fruits and seeds of the middle Eocene Messel biota, Germany. Abhandlungen der Senckenbergischen Naturforschenden Gesellschaft 570: 1–249.

[mcaf240-B7] Del Rio C , Huang J, Liu P, et al 2021. New Eocene fossil fruits and leaves of Menispermaceae from the central Tibetan plateau and their biogeographic implications. Journal of Systematics and Evolution 59: 1287–1306. doi:10.1111/jse.12701

[mcaf240-B8] Deng T , Wu FX, Wang SQ, Su Tao, Zhou Zhekun. 2021. Major turnover of biotas across the Oligocene/Miocene boundary on the Tibetan plateau. Palaeogeography, Palaeoclimatology, Palaeoecology 567: 110241. doi:10.1016/j.palaeo.2021.110241

[mcaf240-B9] Diels L . 1910. Menispermaceae. In: Engler A. ed. Das Pflanzenreich IV, Vol. 94. Leipzig: Wilhelm Engelmann, 1–140.

[mcaf240-B10] Ding WJ , Hou DJ, Gan J, Wu P, Zhang M, George SC. 2021. Palaeovegetation variation in response to the late Oligocene-early Miocene East Asian summer monsoon in the Ying-Qiong Basin, South China Sea. Palaeogeography, Palaeoclimatology, Palaeoecology 567: 110205. doi:10.1016/j.palaeo.2020.110205

[mcaf240-B11] Ding L , Spicer RA, Yang J, et al 2017. Quantifying the rise of the Himalaya orogen and implications for the South Asian monsoon. Geology 45: 215–218. doi:10.1130/G38583.1

[mcaf240-B12] Drummond AJ , Ho S, Phillips M, Rambaut A. 2006. Relaxed phylogenetics and dating with confidence. PLoS Biology 4: e88. doi:10.1371/journal.pbio.0040088 16683862 PMC1395354

[mcaf240-B13] Figueroa C , Salazar GA, Zavaleta HA, Engleman EM. 2008. Root character evolution and systematics in Cranichidinae, Prescottiinae and Spiranthinae (Orchidaceae, Cranichideae). Annals of Botany 101: 509–520. doi:10.1093/aob/mcm328 18263628 PMC2710200

[mcaf240-B14] Finarelli JA , Flynn JJ. 2006. Ancestral state reconstruction of body size in the Caniformia (Carnivora, Mammalia): the effects of incorporating data from the fossil record. Systematic Biology 55: 301–313. doi:10.1080/10635150500541698 16611601

[mcaf240-B15] Fisher D . 1980. The role of stratigraphic data in phylogenetic inference. Geological Society of America 12: 426.

[mcaf240-B16] Fisher DC . 2008. Stratocladistics: integrating temporal data and character data in phylogenetic inference. Annual Review of Ecology, Evolution, and Systematics 39: 365–385. doi:10.1146/annurev.ecolsys.38.091206.095752

[mcaf240-B17] Futuyma DJ . 1979. Evolutionary biology. Sunderland: Sinauer.

[mcaf240-B18] Gavryushkina A , Heath TA, Ksepka DT, Stadler T, Welch D, Drummond AJ. 2017. Bayesian total-evidence dating reveals the recent crown radiation of penguins. Systematic Biology 66: 57–73. doi:10.1093/sysbio/syw060 28173531 PMC5410945

[mcaf240-B19] Gearty W , Allen BJ, Godoy PL, Chiarenza AA. 2023. Including fossil tips often, but not always, vastly improves the reconstruction of trait evolution using phylogenetic comparative methods. Geological Society of America Abstracts 55: 393013. doi:10.1130/abs/2023AM-393013

[mcaf240-B20] Guzmán B , Vargas P. 2005. Systematics, character evolution, and biogeography of *Cistus* L. (Cistaceae) based on ITS, *trnL-trnF*, and *matK* sequences. Molecular Phylogenetics and Evolution 37: 644–660. doi:10.1016/j.ympev.2005.04.026 16055353

[mcaf240-B21] Han M , Manchester SR, Fu QY, Jin JH, Quan C. 2018. Paleogene fossil fruits of *Stephania* (Menispermaceae) from North America and East Asia. Journal of Systematics and Evolution 56: 81–91. doi:10.1111/jse.12288

[mcaf240-B22] Han M , Wu X, Tu M, Kodrul TM, Jin J. 2020. Diversity of Menispermaceae from the Paleocene and Eocene of South China. Journal of Systematics and Evolution 58: 354–366. doi:10.1111/jse.12499

[mcaf240-B23] Heath TA , Huelsenbeck JP, Stadler T. 2014. The fossilized birth-death process for coherent calibration of divergence-time estimates. Proceedings of the National Academy of Sciences of the United States of America 111: E2957–E2966. doi:10.1073/pnas.1319091111 25009181 PMC4115571

[mcaf240-B24] Hermsen EJ , Hendricks JR. 2008. W(h)ither fossils? Studying morphological character evolution in the age of molecular sequences. Annals of the Missouri Botanical Garden 95: 72–100. doi:10.3417/2006206

[mcaf240-B25] Herrera F , Manchester SR, Hoot SB, et al 2011. Phytogeographic implications of fossil endocarps of Menispermaceae from the Paleocene of Colombia. American Journal of Botany 98: 2004–2017. doi:10.3732/ajb.1000461 22114219

[mcaf240-B26] Ho SYW , Phillips MJ. 2009. Accounting for calibration uncertainties in phylogenetic estimation of evolutionary divergence times. Systematic Biology 58: 367–380. doi:10.1093/sysbio/syp035 20525591

[mcaf240-B27] Jablonski D . 2004. Extinction: past and present. Nature 427: 589. doi:10.1038/427589a 14961099

[mcaf240-B28] Jacobs B , Lens F, Smets E. 2009. Evolution of fruit and seed characters in the *Diervilla* and *Lonicera* clades (Caprifoliaceae, Dipsacales). Annals of Botany 104: 253–276. doi:10.1093/aob/mcp131 19502353 PMC2710890

[mcaf240-B29] Jacques FMB . 2009a. Fossil history of the Menispermaceae (Ranunculales). Annales de Paleontologie 95: 53–69. doi:10.1016/j.annpal.2009.03.001

[mcaf240-B30] Jacques FMB . 2009b. Survey of the Menispermaceae endocarps. ADA Forecast 31: 47–87. doi:10.5252/a2009n1a4

[mcaf240-B31] Jacques FMB , Wang W, Ortiz RDC, Zhou ZK, Li HL, Chen ZD. 2011. Integrating fossils in a molecular-based phylogeny and testing them as calibration points for divergence time estimates in Menispermaceae. Journal of Systematics and Evolution 49: 25–49. doi:10.1111/j.1759-6831.2010.00105.x

[mcaf240-B32] Jones KE , Angielczyk KD, Polly PD, et al 2018. Fossils reveal the complex evolutionary history of the mammalian regionalized spine. Science 361: 1249–1252. doi:10.1126/science.aar3126 30237356

[mcaf240-B33] Jud NA , Iglesias A, Wilf P, Gandolfo MA. 2018. Fossil moonseeds from the Paleogene of West Gondwana (Patagonia Argentina). American Journal of Botany 105: 927–942. doi:10.1002/ajb2.1092 29882954

[mcaf240-B34] Kearse M , Moir R, Wilson A, et al 2012. Geneious basic: an integrated and extendable desktop software platform for the organization and analysis of sequence data. Bioinformatics 28: 1647–1649. doi:10.1093/bioinformatics/bts199 22543367 PMC3371832

[mcaf240-B35] King B . 2021. Bayesian tip-dated phylogenetics in paleontology: topological effects and stratigraphic fit. Systematic Biology 70: 283–294. doi:10.1093/sysbio/syaa057 32692834

[mcaf240-B36] King B , Qiao T, Lee MSY, Zhu M, Long JA. 2017. Bayesian morphological clock methods resurrect placoderm monophyly and reveal rapid early evolution in jawed vertebrates. Systematic Biology 66: 499–516. doi:10.1093/sysbio/syw107 27920231

[mcaf240-B37] Ksepka DT , Stidham TA, Williamson TE. 2017. Early Paleocene landbird supports rapid phylogenetic and morphological diversification of crown birds after the K-Pg mass extinction. Proceedings of the National Academy of Sciences of the United States of America 114: 8047–8052. doi:10.1073/pnas.1700188114 28696285 PMC5544281

[mcaf240-B38] Lewis PO . 2001. A likelihood approach to estimating phylogeny from discrete morphological character data. Systematic Biology 50: 913–925. doi:10.1080/106351501753462876 12116640

[mcaf240-B39] Li XQ , Peng HW, Xiang XG, et al 2024. Phylogenetic evidence clarifies the history of the extrusion of Indochina. Proceedings of the National Academy of Sciences of the United States of America 121: e2322527121. doi:10.1073/pnas.2322527121 39159371 PMC11363272

[mcaf240-B40] Lian L , Ortiz RDC, Jabbour F, et al 2020. Phylogeny and biogeography of Pachygoneae (Menispermaceae), with consideration of the boreotropical flora hypothesis and resurrection of the genera *Cebatha* and *Nephroia*. Molecular Phylogenetics and Evolution 148: 106825. doi:10.1016/j.ympev.2020.106825 32294547

[mcaf240-B41] Lian L , Peng HW, Erst AS, et al 2024. Bayesian tip-dated phylogeny and biogeography of Cissampelideae (Menispermaceae): mitigating the effects of homoplastic morphological characters. Cladistics 40: 391–410. doi:10.1111/cla.12573 38469932

[mcaf240-B42] Louca S , Pennell MW. 2020. Extant timetrees are consistent with a myriad of diversification histories. Nature 580: 502–505. doi:10.1038/s41586-020-2176-1 32322065

[mcaf240-B43] Luo XR . 1982. A systematic notes on the genus *Stephania* of China. Bulletin of Botanical Research 2: 33–59.

[mcaf240-B44] Luo X , Lo HS, Tao C, Gilbert MG. 2008. Menispermaceae. In: Wu Z, Raven PH. eds. Flora of China, Vol. 7. Beijing: Science Press; St Louis: Missouri Botanical Garden Press, 1–31.

[mcaf240-B45] Maddison WP , Maddison DR. 2023. Mesquite: a modular system for evolutionary analysis. Version 3.81. http://www.mesquiteproject.org (23 March 2025, date last accessed).

[mcaf240-B46] Mao F , Zhang C, Liu C, Meng J. 2021. Fossoriality and evolutionary development in two Cretaceous mammaliamorphs. Nature 592: 577–582. doi:10.1038/s41586-021-03433-2 33828300

[mcaf240-B47] Marshall CR . 2017. Five palaeobiological laws needed to understand the evolution of the living biota. Nature Ecology & Evolution 1: 165. doi:10.1038/s41559-017-0165 28812640

[mcaf240-B48] Mongiardino Koch N , Garwood RJ, Parry LA. 2021. Fossils improve phylogenetic analyses of morphological characters. Proceedings of the Royal Society of London B 288: 20210044. doi:10.1098/rspb.2021.0044 PMC8246652 33947239

[mcaf240-B49] O’Reilly JE , dos Reis M, Donoghue PCJ. 2015. Dating tips for divergence-time estimation. Trends in Genetics 31: 637–650. doi:10.1016/j.tig.2015.08.001 26439502

[mcaf240-B50] Ortiz RDC , Wang W, Jacques FMB, Chen ZD. 2016. Phylogeny and a revised tribal classification of Menispermaceae based on molecular and morphological data. Taxon 65: 1288–1312. doi:10.12705/656.5

[mcaf240-B51] Parham JF , Donoghue PCJ, Bell CJ, et al 2012. Best practices for justifying fossil calibrations. Systematic Biology 61: 346–359. doi:10.1093/sysbio/syr107 22105867 PMC3280042

[mcaf240-B52] Rambaut A , Drummond AJ, Xie D, Baele G, Suchard MA. 2018. Posterior summarization in Bayesian phylogenetics using Tracer 1.7. Systematic Biology 67: 901–904. doi:10.1093/sysbio/syy032 29718447 PMC6101584

[mcaf240-B53] Ronquist F , Teslenko M, Van der Mark P, et al 2012. MrBayes 3.2: efficient Bayesian phylogenetic inference and model choice across a large model space. Systematic Biology 61: 539–542. doi:10.1093/sysbio/sys029 22357727 PMC3329765

[mcaf240-B54] Slater GJ . 2013. Phylogenetic evidence for a shift in the mode of mammalian body size evolution at the Cretaceous-Palaeogene boundary. Methods in Ecology and Evolution 4: 734–744. doi:10.1111/2041-210X.12084

[mcaf240-B55] Slater GJ , Harmon LJ, Alfaro ME. 2012. Integrating fossils with molecular phylogenies improves inference of trait evolution. Evolution 66: 3931–3944. doi:10.1111/j.1558-5646.2012.01723.x 23206147

[mcaf240-B56] Smith AB . 2000. Stratigraphy in phylogeny reconstruction. Journal of Paleontology 74: 763–766. doi:10.1666/0022-3360(2000)074<0763:SIPR>2.0.CO;2

[mcaf240-B57] Stamatakis A . 2014. RAxML version 8: a tool for phylogenetic analysis and post-analysis of large phylogenies. Bioinformatics 30: 1312–1313. doi:10.1093/bioinformatics/btu033 24451623 PMC3998144

[mcaf240-B58] Turner AH , Pritchard AC, Matzke NJ. 2017. Empirical and Bayesian approaches to fossil only divergence times: a study across three reptile clades. PLoS One 12: e0169885. doi:10.1371/journal.pone.0169885 28187191 PMC5302793

[mcaf240-B59] Wang W , Chen ZD. 2007. Generic level phylogeny of Thalictroideae (Ranunculaceae) – implications for the taxonomic status of *Paropyrum* and petal evolution. Taxon 56: 811–821. doi:10.2307/25065863

[mcaf240-B60] Wang W , Lin L, Xiang XG, et al 2016. The rise of angiosperm-dominated herbaceous floras: insights from Ranunculaceae. Scientific Reports 6: 27259. doi:10.1038/srep27259 27251635 PMC4890112

[mcaf240-B61] Wang W , Ortiz RDC, Jacques FMB, et al 2012. Menispermaceae and the diversification of tropical rainforests near the Cretaceous-Paleogene boundary. New Phytologist 195: 470–478. doi:10.1111/j.1469-8137.2012.04158.x 22548458

[mcaf240-B62] Wefferling KM , Hoot SB, Neves SS. 2013. Phylogeny and fruit evolution in Menispermaceae. American Journal of Botany 100: 883–905. doi:10.3732/ajb.1200556 23608646

[mcaf240-B63] Wright A , Wagner PJ, Wright DF. 2021. Testing character evolution models in phylogenetic paleobiology: a case study with Cambrian echinoderms. Cambridge: Cambridge University Press.

[mcaf240-B64] Zheng H . 2015. Birth of the Yangtze River: age and tectonic-geomorphic implications. National Science Review 2: 438–453. doi:10.1093/nsr/nwv063

