# Supplementary material for: The endocarp evolution of Cissampelideae (Menispermaceae): integrating extant and fossil species
Source: Ann Bot. 2025 Oct 22;137(6):2015–24. doi: 10.1093/aob/mcaf240 (PMC13274978; doi:10.1093/aob/mcaf240)
Supplement: mcaf240_Supplementary_Data [file mcaf240_supplementary_data.zip › Lian et al.-Revised Fig. S3.pdf]

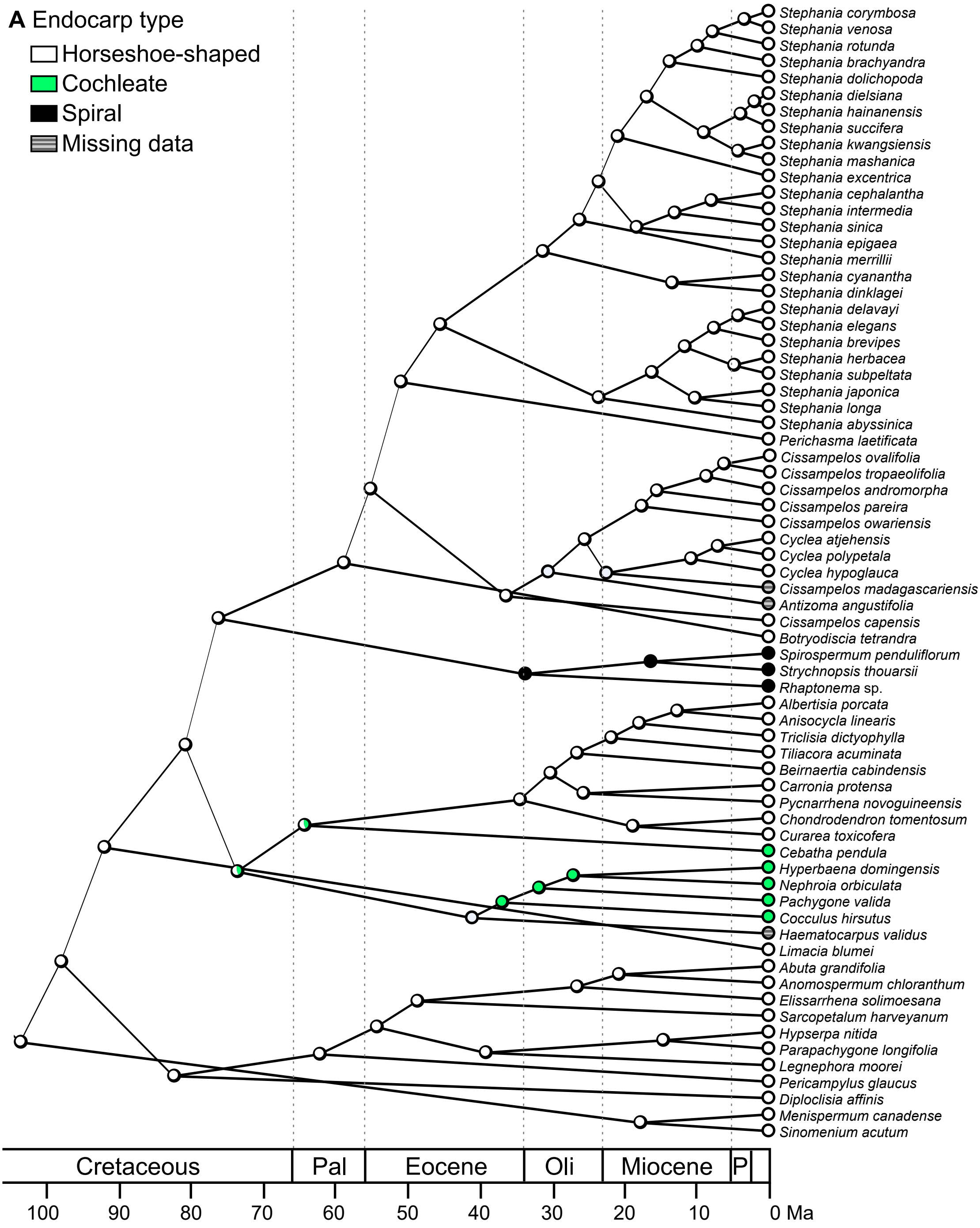

A Endocarp type
Horseshoe-shaped
Cochleate
Spiral
Missing data
Stephania corymbosa
Stephania venosa
Stephania rotunda
Stephania brachyandra
Stephania dolichopoda
Stephania dielsiana
Stephania hainanensis
Stephania succifera
Stephania kwangsiensis
Stephania mashanica
Stephania excentrica
Stephania cephalantha
Stephania intermedia
Stephania sinica
Stephania epigaea
Stephania merrillii
Stephania cyanantha
Stephania dinklagei
Stephania delavayi
Stephania elegans
Stephania brevipes
Stephania herbacea
Stephania subpeltata
Stephania japonica
Stephania longa
Stephania abyssinica
Perichasma laetificata
Cissampelos ovalifolia
Cissampelos tropaeolifolia
Cissampelos andromorpha
Cissampelos pareira
Cissampelos owariensis
Cyclea atjehensis
Cyclea polypetala
Cyclea hypoglauca
Cissampelos madagascariensis
Antizoma angustifolia
Cissampelos capensis
Botryodiscia tetrandra
Spirospermum penduliflorum
Strychnopsis thouarsii
Rhaptonema sp.
Albertisia porcata
Anisocycla linearis
Triclisia dictyophylla
Tiliacora acuminata
Beirnaertia cabindensis
Carronia protensa
Pycnarrhena novoguineensis
Chondrodendron tomentosum
Curarea toxicofera
Cebatha pendula
Hyperbaena domingensis
Nephroia orbiculata
Pachygone valida
Cocculus hirsutus
Haematocarpus validus
Limacia blumei
Abuta grandifolia
Anomospermum chloranthum
Elissarrhena solimoesana
Sarcopetalum harveyanum
Hypserpa nitida
Parapachygone longifolia
Legnephora moorei
Pericampylus glaucus
Diploclisia affinis
Menispermum canadense
Sinomenium acutum
Cretaceous
Pal
Eocene
Oli
Miocene
P
100
90
80
70
60
50
40
30
20
10
0 Ma

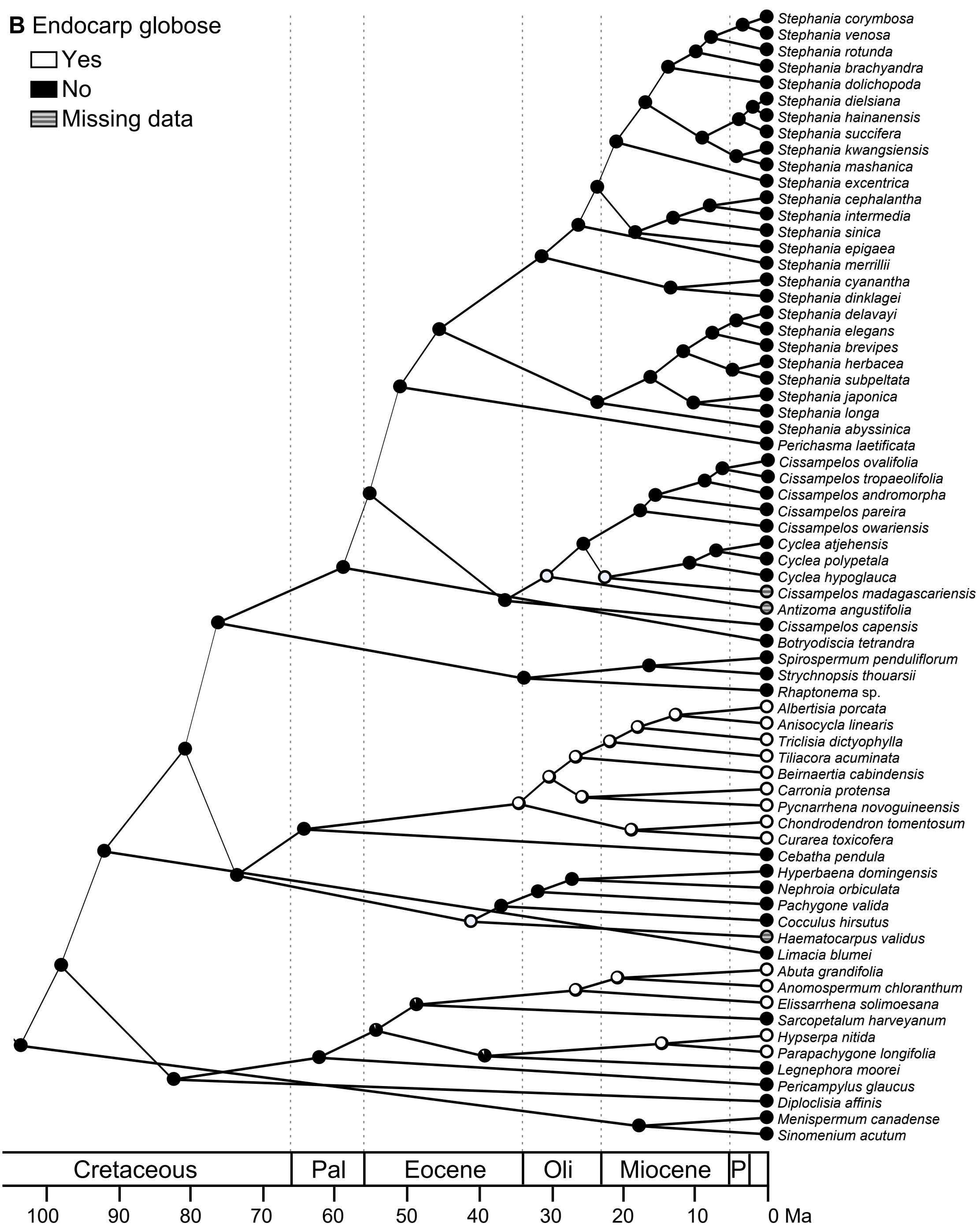

B Endocarp globose
Yes
No
Missing data
Stephania corymbosa
Stephania venosa
Stephania rotunda
Stephania brachyandra
Stephania dolichopoda
Stephania dielsiana
Stephania hainanensis
Stephania succifera
Stephania kwangsiensis
Stephania mashanica
Stephania excentrica
Stephania cephalantha
Stephania intermedia
Stephania sinica
Stephania epigaea
Stephania merrillii
Stephania cyanantha
Stephania dinklagei
Stephania delavayi
Stephania elegans
Stephania brevipes
Stephania herbacea
Stephania subpeltata
Stephania japonica
Stephania longa
Stephania abyssinica
Perichasma laetificata
Cissampelos ovalifolia
Cissampelos tropaeolifolia
Cissampelos andromorpha
Cissampelos pareira
Cissampelos owariensis
Cyclea atjehensis
Cyclea polypetala
Cyclea hypoglauca
Cissampelos madagascariensis
Antizoma angustifolia
Cissampelos capensis
Botryodiscia tetrandra
Spirospermum penduliflorum
Strychnopsis thouarsii
Rhaptonema sp.
Albertisia porcata
Anisocycla linearis
Triclisia dictyophylla
Tiliacora acuminata
Beirnaertia cabindensis
Carronia protensa
Pycnarrhena novoguineensis
Chondrodendron tomentosum
Curarea toxicofera
Cebatha pendula
Hyperbaena domingensis
Nephroia orbiculata
Pachygone valida
Cocculus hirsutus
Haematocarpus validus
Limacia blumei
Abuta grandifolia
Anomospermum chloranthum
Elissarrhena solimoesana
Sarcopetalum harveyanum
Hypserpa nitida
Parapachygone longifolia
Legnephora moorei
Pericampylus glaucus
Diploclisia affinis
Menispermum canadense
Sinomenium acutum
Cretaceous
Pal
Eocene
Oli
Miocene
P
100
90
80
70
60
50
40
30
20
10
0 Ma

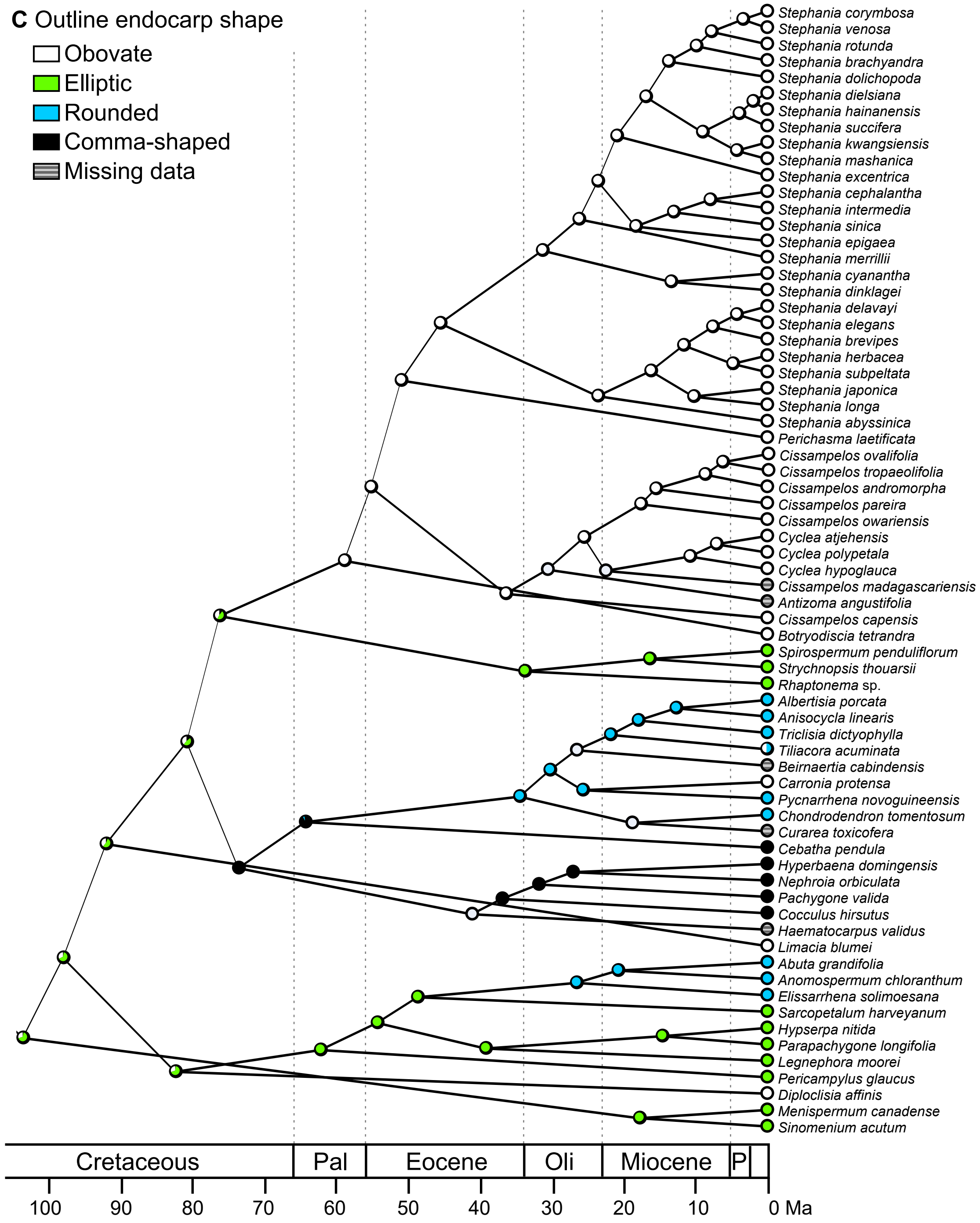
C Outline endocarp shape
Obovate
Elliptic
Rounded
Comma-shaped
Missing data
Stephania corymbosa
Stephania venosa
Stephania rotunda
Stephania brachyandra
Stephania dolichopoda
Stephania dielsiana
Stephania hainanensis
Stephania succifera
Stephania kwangsiensis
Stephania mashanica
Stephania excentrica
Stephania cephalantha
Stephania intermedia
Stephania sinica
Stephania epigaea
Stephania merrillii
Stephania cyanantha
Stephania dinklagei
Stephania delavayi
Stephania elegans
Stephania brevipes
Stephania herbacea
Stephania subpeltata
Stephania japonica
Stephania longa
Stephania abyssinica
Perichasma laetificata
Cissampelos ovalifolia
Cissampelos tropaeolifolia
Cissampelos andromorpha
Cissampelos pareira
Cissampelos owariensis
Cyclea atjehensis
Cyclea polypetala
Cyclea hypoglauca
Cissampelos madagascariensis
Antizoma angustifolia
Cissampelos capensis
Botryodiscia tetrandra
Spirospermum penduliflorum
Strychnopsis thouarsii
Rhaptonema sp.
Albertisia porcata
Anisocycla linearis
Triclisia dictyophylla
Tiliacora acuminata
Beirnaertia cabindensis
Carronia protensa
Pycnarrhena novoguineensis
Chondrodendron tomentosum
Curarea toxicofera
Cebatha pendula
Hyperbaena domingensis
Nephroia orbiculata
Pachygone valida
Cocculus hirsutus
Haematocarpus validus
Limacia blumei
Abuta grandifolia
Anomospermum chloranthum
Elissarrhena solimoesana
Sarcopetalum harveyanum
Hypserpa nitida
Parapachygone longifolia
Legnephora moorei
Pericampylus glaucus
Diploclisia affinis
Menispermum canadense
Sinomenium acutum
Cretaceous
Pal
Eocene
Oli
Miocene
P
100
90
80
70
60
50
40
30
20
10
0 Ma

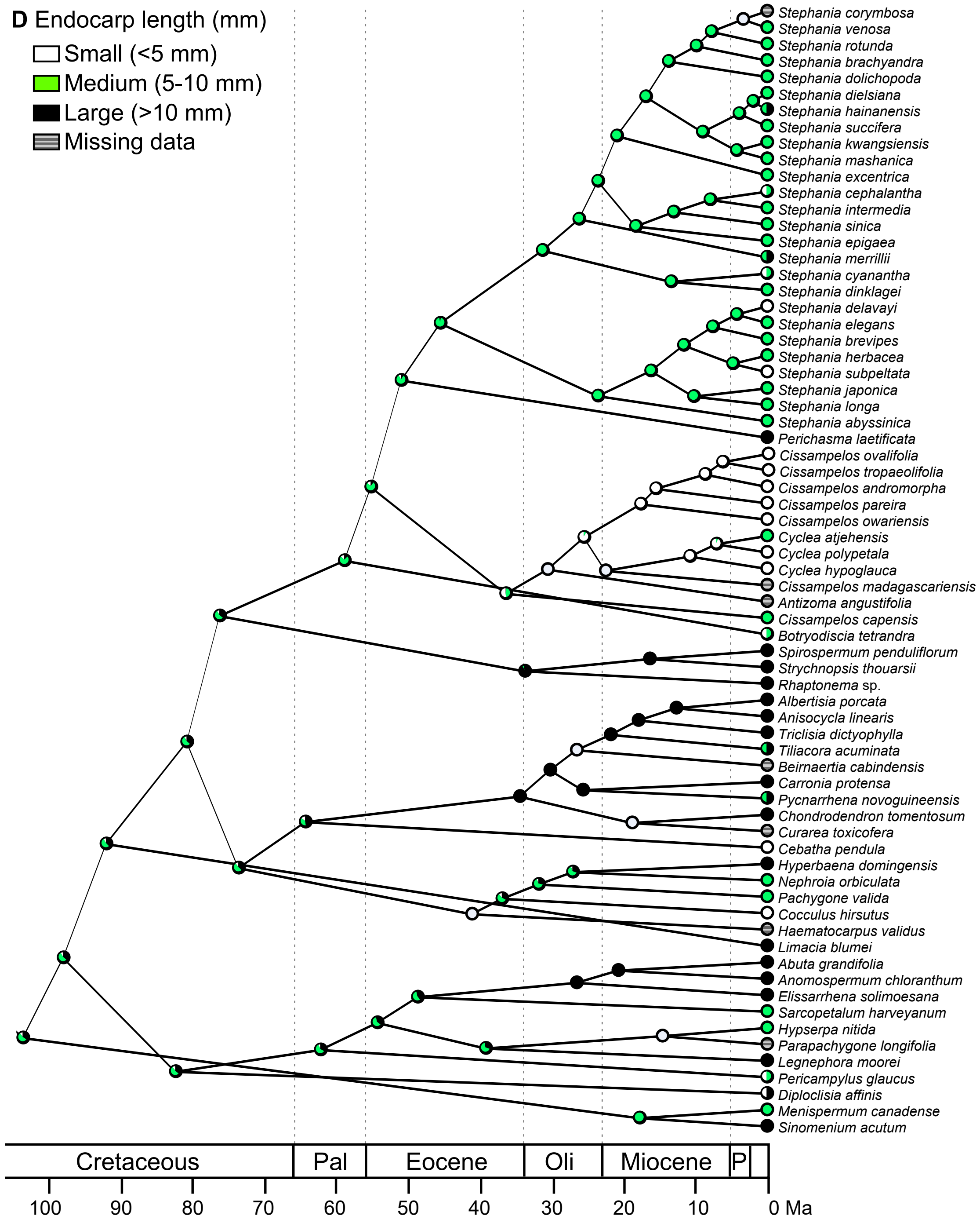

D Endocarp length (mm)
Small (<5 mm)
Medium (5-10 mm)
Large (>10 mm)
Missing data
Stephania corymbosa
Stephania venosa
Stephania rotunda
Stephania brachyandra
Stephania dolichopoda
Stephania dielsiana
Stephania hainanensis
Stephania succifera
Stephania kwangsiensis
Stephania mashanica
Stephania excentrica
Stephania cephalantha
Stephania intermedia
Stephania sinica
Stephania epigaea
Stephania merrillii
Stephania cyanantha
Stephania dinklagei
Stephania delavayi
Stephania elegans
Stephania brevipes
Stephania herbacea
Stephania subpeltata
Stephania japonica
Stephania longa
Stephania abyssinica
Perichasma laetificata
Cissampelos ovalifolia
Cissampelos tropaeolifolia
Cissampelos andromorpha
Cissampelos pareira
Cissampelos owariensis
Cyclea atjehensis
Cyclea polypetala
Cyclea hypoglauca
Cissampelos madagascariensis
Antizoma angustifolia
Cissampelos capensis
Botryodiscia tetrandra
Spirospermum penduliflorum
Strychnopsis thouarsii
Rhaptonema sp.
Albertisia porcata
Anisocycla linearis
Triclisia dictyophylla
Tiliacora acuminata
Beirnaertia cabindensis
Carronia protensa
Pycnarrhena novoguineensis
Chondrodendron tomentosum
Curarea toxicofera
Cebatha pendula
Hyperbaena domingensis
Nephroia orbiculata
Pachygone valida
Cocculus hirsutus
Haematocarpus validus
Limacia blumei
Abuta grandifolia
Anomospermum chloranthum
Elissarrhena solimoesana
Sarcopetalum harveyanum
Hypserpa nitida
Parapachygone longifolia
Legnephora moorei
Pericampylus glaucus
Diploclisia affinis
Menispermum canadense
Sinomenium acutum
Cretaceous
Pal
Eocene
Oli
Miocene
P
100
90
80
70
60
50
40
30
20
10
0 Ma

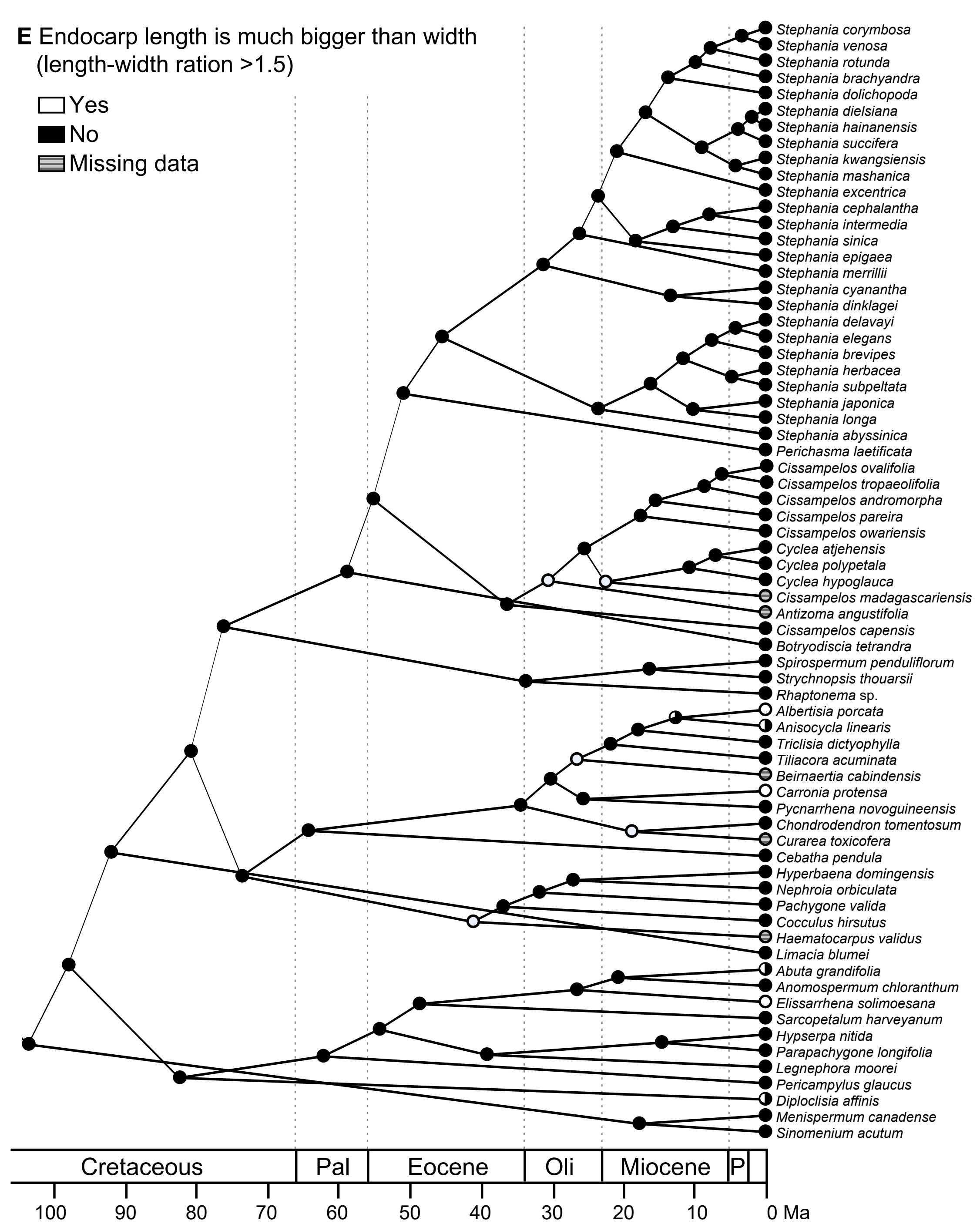

E Endocarp length is much bigger than width (length-width ration >1.5)
Yes
No
Missing data
Stephania corymbosa
Stephania venosa
Stephania rotunda
Stephania brachyandra
Stephania dolichopoda
Stephania dielsiana
Stephania hainanensis
Stephania succifera
Stephania kwangsiensis
Stephania mashanica
Stephania excentrica
Stephania cephalantha
Stephania intermedia
Stephania sinica
Stephania epigaea
Stephania merrillii
Stephania cyanantha
Stephania dinklagei
Stephania delavayi
Stephania elegans
Stephania brevipes
Stephania herbacea
Stephania subpeltata
Stephania japonica
Stephania longa
Stephania abyssinica
Perichasma laetificata
Cissampelos ovalifolia
Cissampelos tropaeolifolia
Cissampelos andromorpha
Cissampelos pareira
Cissampelos owariensis
Cyclea atjehensis
Cyclea polypetala
Cyclea hypoglauca
Cissampelos madagascariensis
Antizoma angustifolia
Cissampelos capensis
Botryodiscia tetrandra
Spirospermum penduliflorum
Strychnopsis thouarsii
Rhaptonema sp.
Albertisia porcata
Anisocycla linearis
Triclisia dictyophylla
Tiliacora acuminata
Beirnaertia cabindensis
Carronia protensa
Pycnarrhena novoguineensis
Chondrodendron tomentosum
Curarea toxicofera
Cebatha pendula
Hyperbaena domingensis
Nephroia orbiculata
Pachygone valida
Cocculus hirsutus
Haematocarpus validus
Limacia blumei
Abuta grandifolia
Anomospermum chloranthum
Elissarrhena solimoesana
Sarcopetalum harveyanum
Hypserpa nitida
Parapachygone longifolia
Legnephora moorei
Pericampylus glaucus
Diploclisia affinis
Menispermum canadense
Sinomenium acutum
Cretaceous
Pal
Eocene
Oli
Miocene
P
100
90
80
70
60
50
40
30
20
10
0 Ma

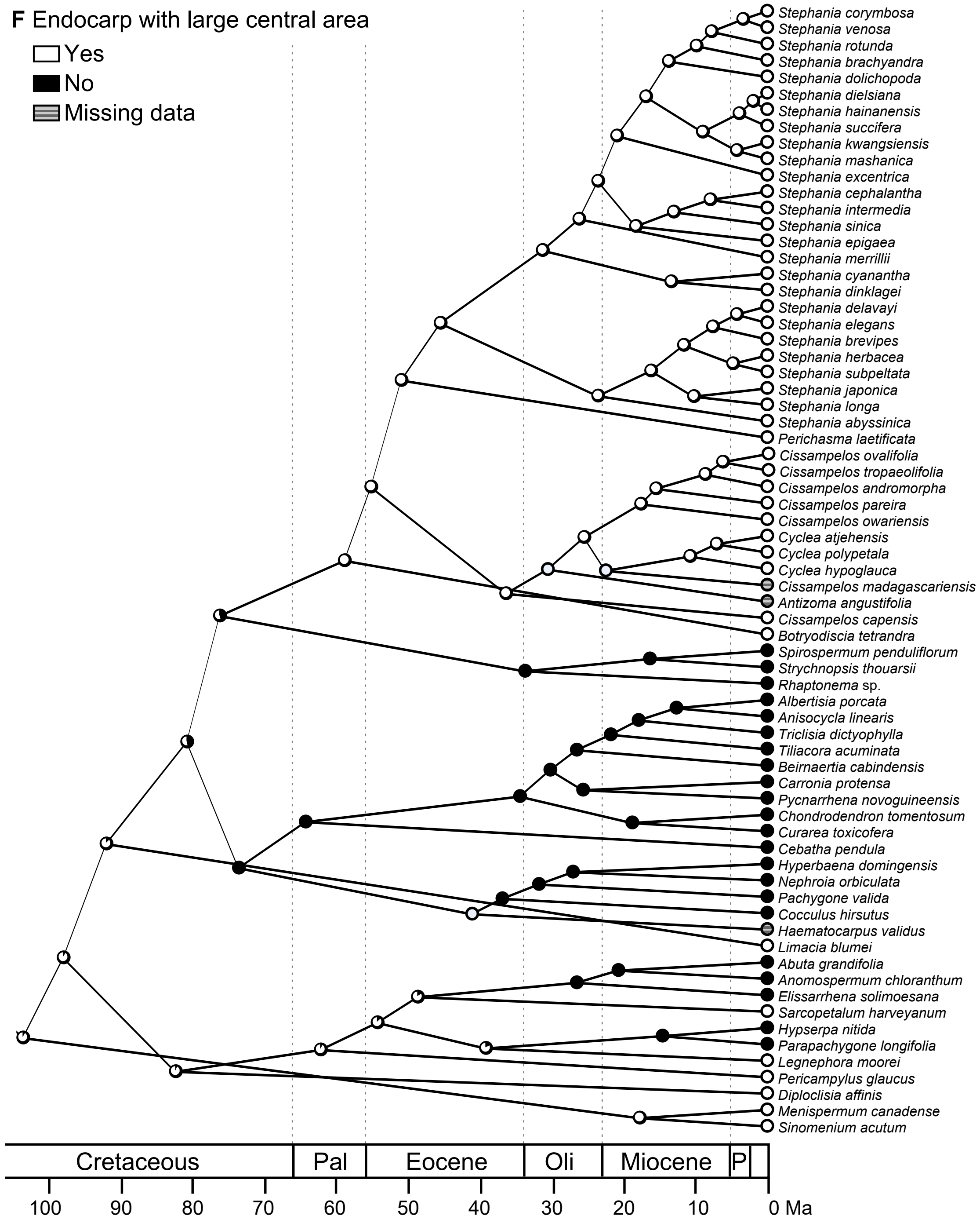
F Endocarp with large central area
Yes
No
Missing data
Stephania corymbosa
Stephania venosa
Stephania rotunda
Stephania brachyandra
Stephania dolichopoda
Stephania dielsiana
Stephania hainanensis
Stephania succifera
Stephania kwangsiensis
Stephania mashanica
Stephania excentrica
Stephania cephalantha
Stephania intermedia
Stephania sinica
Stephania epigaea
Stephania merrillii
Stephania cyanantha
Stephania dinklagei
Stephania delavayi
Stephania elegans
Stephania brevipes
Stephania herbacea
Stephania subpeltata
Stephania japonica
Stephania longa
Stephania abyssinica
Perichasma laetificata
Cissampelos ovalifolia
Cissampelos tropaeolifolia
Cissampelos andromorpha
Cissampelos pareira
Cissampelos owariensis
Cyclea atjehensis
Cyclea polypetala
Cyclea hypoglauca
Cissampelos madagascariensis
Antizoma angustifolia
Cissampelos capensis
Botryodiscia tetrandra
Spirospermum penduliflorum
Strychnopsis thouarsii
Rhaptonema sp.
Albertisia porcata
Anisocycla linearis
Triclisia dictyophylla
Tiliacora acuminata
Beirnaertia cabindensis
Carronia protensa
Pycnarrhena novoguineensis
Chondrodendron tomentosum
Curarea toxicofera
Cebatha pendula
Hyperbaena domingensis
Nephroia orbiculata
Pachygone valida
Cocculus hirsutus
Haematocarpus validus
Limacia blumei
Abuta grandifolia
Anomospermum chloranthum
Elissarrhena solimoesana
Sarcopetalum harveyanum
Hypserpa nitida
Parapachygone longifolia
Legnephora moorei
Pericampylus glaucus
Diploclisia affinis
Menispermum canadense
Sinomenium acutum
Cretaceous
Pal
Eocene
Oli
Miocene
P
100
90
80
70
60
50
40
30
20
10
0 Ma

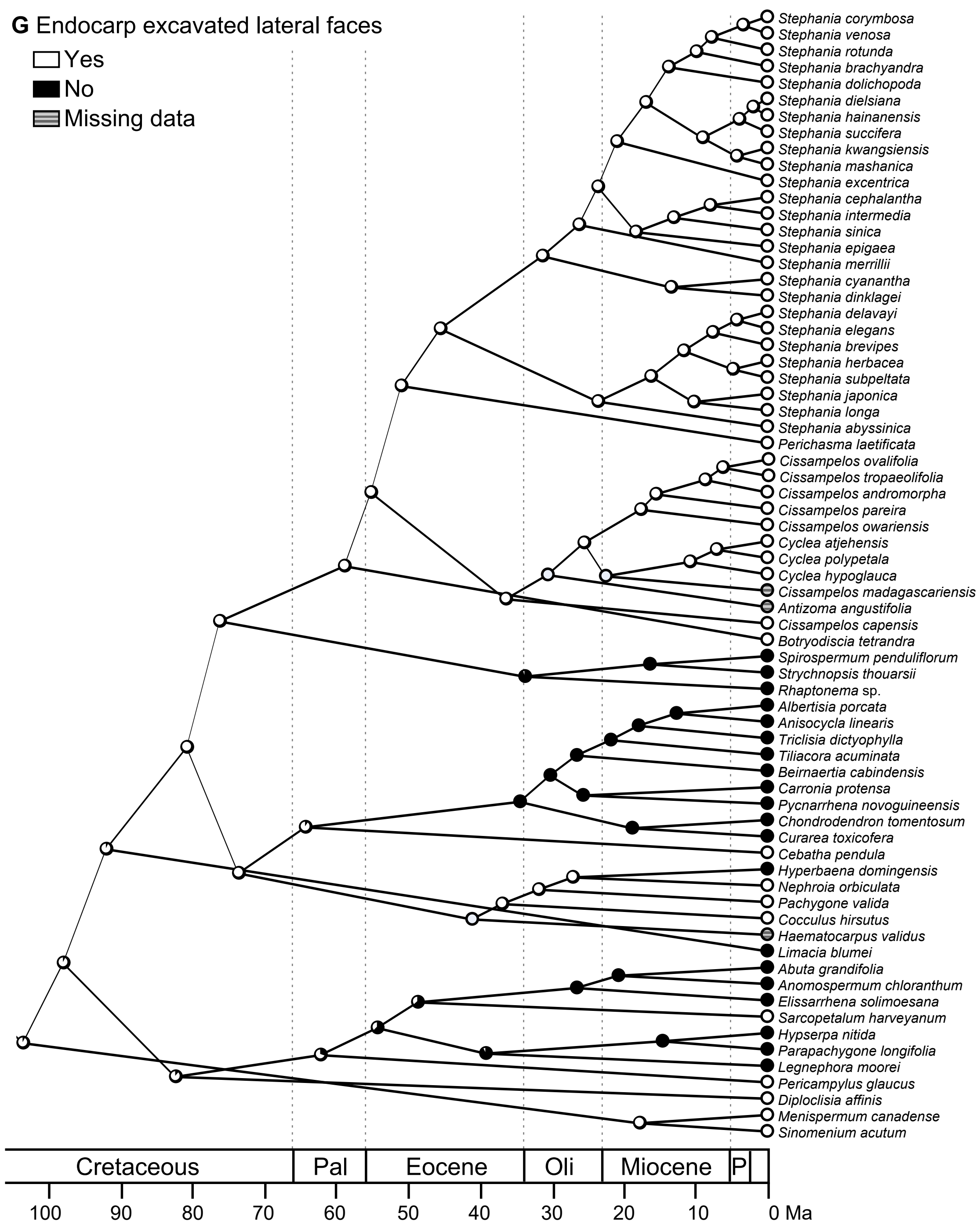

G Endocarp excavated lateral faces
Yes
No
Missing data
Stephania corymbosa
Stephania venosa
Stephania rotunda
Stephania brachyandra
Stephania dolichopoda
Stephania dielsiana
Stephania hainanensis
Stephania succifera
Stephania kwangsiensis
Stephania mashanica
Stephania excentrica
Stephania cephalantha
Stephania intermedia
Stephania sinica
Stephania epigaea
Stephania merrillii
Stephania cyanantha
Stephania dinklagei
Stephania delavayi
Stephania elegans
Stephania brevipes
Stephania herbacea
Stephania subpeltata
Stephania japonica
Stephania longa
Stephania abyssinica
Perichasma laetificata
Cissampelos ovalifolia
Cissampelos tropaeolifolia
Cissampelos andromorpha
Cissampelos pareira
Cissampelos owariensis
Cyclea atjehensis
Cyclea polypetala
Cyclea hypoglauca
Cissampelos madagascariensis
Antizoma angustifolia
Cissampelos capensis
Botryodiscia tetrandra
Spirospermum penduliflorum
Strychnopsis thouarsii
Rhaptonema sp.
Albertisia porcata
Anisocycla linearis
Triclisia dictyophylla
Tiliacora acuminata
Beirnaertia cabindensis
Carronia protensa
Pycnarrhena novoguineensis
Chondrodendron tomentosum
Curarea toxicofera
Cebatha pendula
Hyperbaena domingensis
Nephroia orbiculata
Pachygone valida
Cocculus hirsutus
Haematocarpus validus
Limacia blumei
Abuta grandifolia
Anomospermum chloranthum
Elissarrhena solimoesana
Sarcopetalum harveyanum
Hypserpa nitida
Parapachygone longifolia
Legnephora moorei
Pericampylus glaucus
Diploclisia affinis
Menispermum canadense
Sinomenium acutum
Cretaceous
Pal
Eocene
Oli
Miocene
P
100
90
80
70
60
50
40
30
20
10
0 Ma

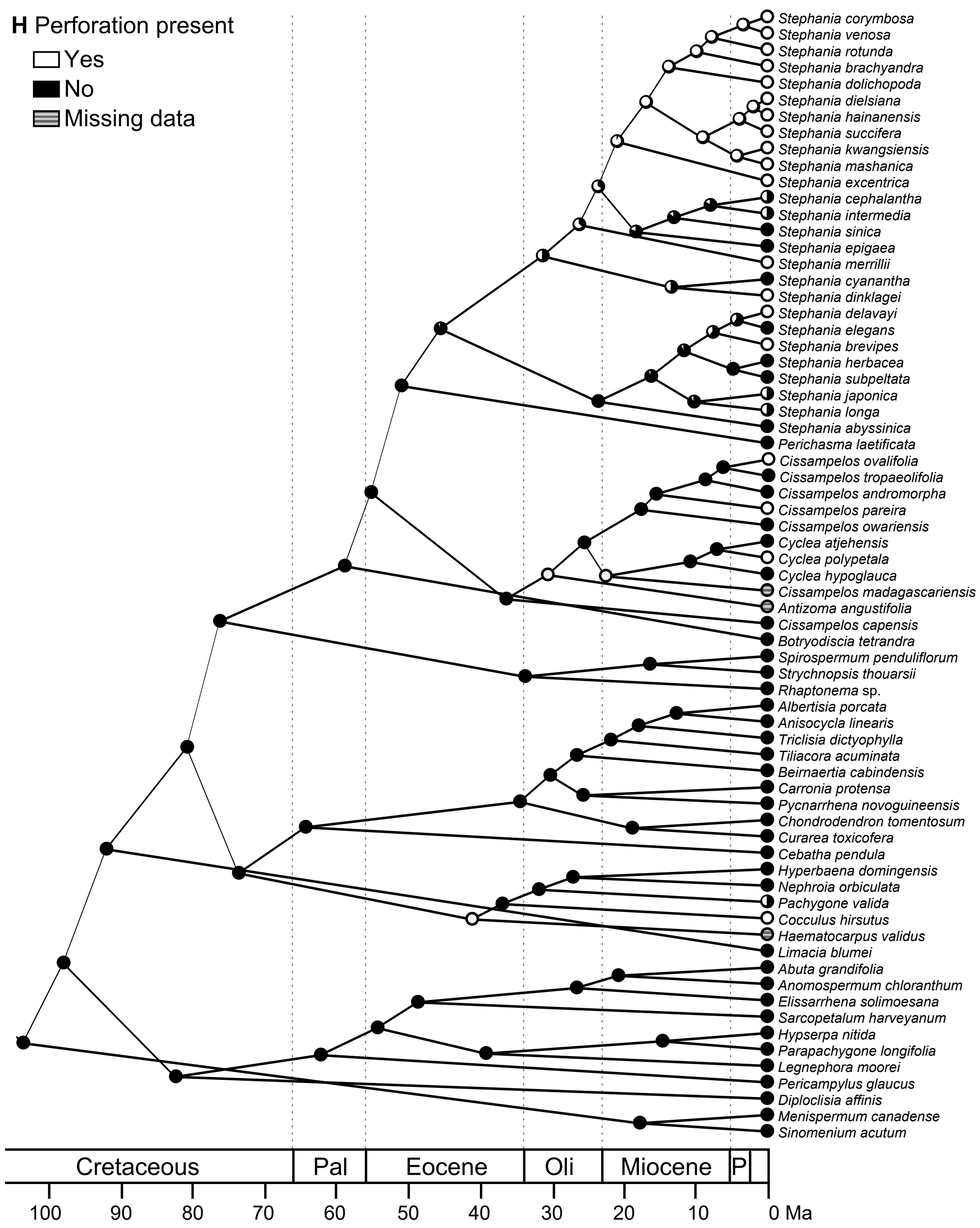
H Perforation present
Yes
No
Missing data
Stephania corymbosa
Stephania venosa
Stephania rotunda
Stephania brachyandra
Stephania dolichopoda
Stephania dielsiana
Stephania hainanensis
Stephania succifera
Stephania kwangsiensis
Stephania mashanica
Stephania excentrica
Stephania cephalantha
Stephania intermedia
Stephania sinica
Stephania epigaea
Stephania merrillii
Stephania cyanantha
Stephania dinklagei
Stephania delavayi
Stephania elegans
Stephania brevipes
Stephania herbacea
Stephania subpeltata
Stephania japonica
Stephania longa
Stephania abyssinica
Perichasma laetificata
Cissampelos ovalifolia
Cissampelos tropaeolifolia
Cissampelos andromorpha
Cissampelos pareira
Cissampelos owariensis
Cyclea atjehensis
Cyclea polypetala
Cyclea hypoglauca
Cissampelos madagascariensis
Antizoma angustifolia
Cissampelos capensis
Botryodiscia tetrandra
Spirospermum penduliflorum
Strychnopsis thouarsii
Rhaptonema sp.
Albertisia porcata
Anisocycla linearis
Triclisia dictyophylla
Tiliacora acuminata
Beirnaertia cabindensis
Carronia protensa
Pycnarrhena novoguineensis
Chondrodendron tomentosum
Curarea toxicofera
Cebatha pendula
Hyperbaena domingensis
Nephroia orbiculata
Pachygone valida
Cocculus hirsutus
Haematocarpus validus
Limacia blumei
Abuta grandifolia
Anomospermum chloranthum
Elissarrhena solimoesana
Sarcopetalum harveyanum
Hypserpa nitida
Parapachygone longifolia
Legnephora moorei
Pericampylus glaucus
Diploclisia affinis
Menispermum canadense
Sinomenium acutum
Cretaceous
Pal
Eocene
Oli
Miocene
P
100
90
80
70
60
50
40
30
20
10
0 Ma

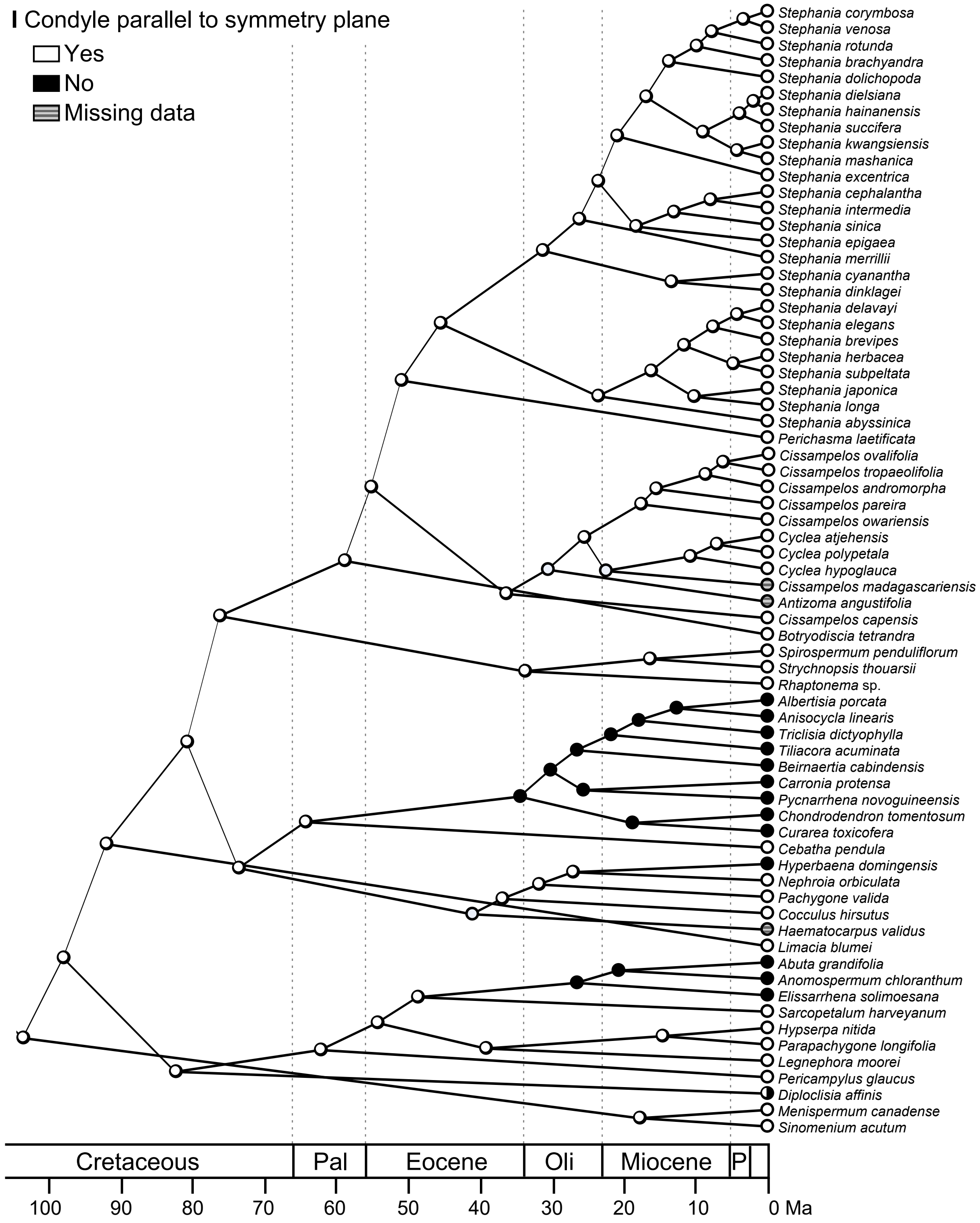

Condyle parallel to symmetry plane
Yes
No
Missing data
Stephania corymbosa
Stephania venosa
Stephania rotunda
Stephania brachyandra
Stephania dolichopoda
Stephania dielsiana
Stephania hainanensis
Stephania succifera
Stephania kwangsiensis
Stephania mashanica
Stephania excentrica
Stephania cephalantha
Stephania intermedia
Stephania sinica
Stephania epigaea
Stephania merrillii
Stephania cyanantha
Stephania dinklagei
Stephania delavayi
Stephania elegans
Stephania brevipes
Stephania herbacea
Stephania subpeltata
Stephania japonica
Stephania longa
Stephania abyssinica
Perichasma laetificata
Cissampelos ovalifolia
Cissampelos tropaeolifolia
Cissampelos andromorpha
Cissampelos pareira
Cissampelos owariensis
Cyclea atjehensis
Cyclea polypetala
Cyclea hypoglauca
Cissampelos madagascariensis
Antizoma angustifolia
Cissampelos capensis
Botryodiscia tetrandra
Spirospermum penduliflorum
Strychnopsis thouarsii
Rhaptonema sp.
Albertisia porcata
Anisocycla linearis
Triclisia dictyophylla
Tiliacora acuminata
Beirnaertia cabindensis
Carronia protensa
Pycnarrhena novoguineensis
Chondrodendron tomentosum
Curarea toxicofera
Cebatha pendula
Hyperbaena domingensis
Nephroia orbiculata
Pachygone valida
Cocculus hirsutus
Haematocarpus validus
Limacia blumei
Abuta grandifolia
Anomospermum chloranthum
Elissarrhena solimoesana
Sarcopetalum harveyanum
Hypserpa nitida
Parapachygone longifolia
Legnephora moorei
Pericampylus glaucus
Diploclisia affinis
Menispermum canadense
Sinomenium acutum
Cretaceous
Pal
Eocene
Oli
Miocene
P
100
90
80
70
60
50
40
30
20
10
0 Ma

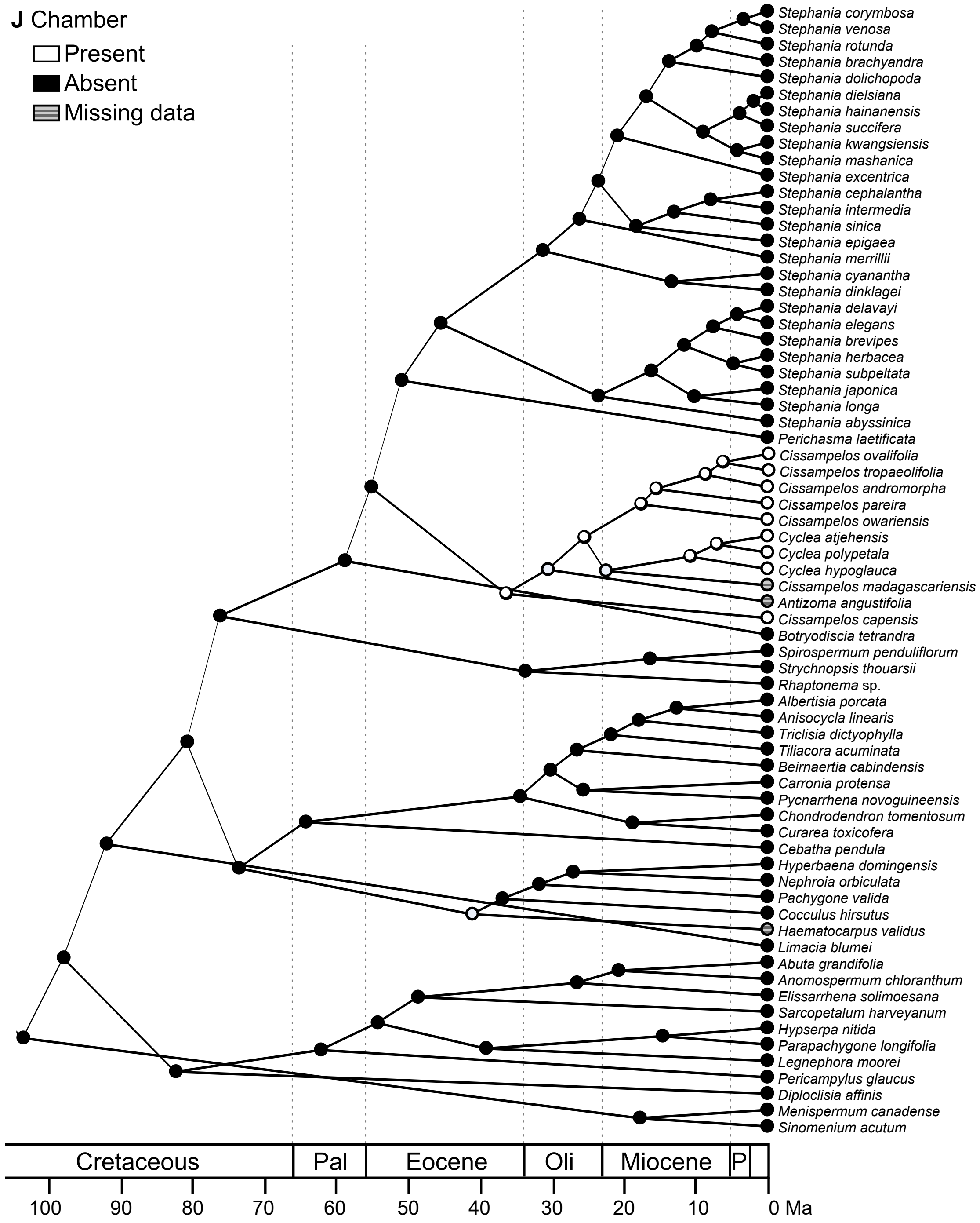
J Chamber
Present
Absent
Missing data
Stephania corymbosa
Stephania venosa
Stephania rotunda
Stephania brachyandra
Stephania dolichopoda
Stephania dielsiana
Stephania hainanensis
Stephania succifera
Stephania kwangsiensis
Stephania mashanica
Stephania excentrica
Stephania cephalantha
Stephania intermedia
Stephania sinica
Stephania epigaea
Stephania merrillii
Stephania cyanantha
Stephania dinklagei
Stephania delavayi
Stephania elegans
Stephania brevipes
Stephania herbacea
Stephania subpeltata
Stephania japonica
Stephania longa
Stephania abyssinica
Perichasma laetificata
Cissampelos ovalifolia
Cissampelos tropaeolifolia
Cissampelos andromorpha
Cissampelos pareira
Cissampelos owariensis
Cyclea atjehensis
Cyclea polypetala
Cyclea hypoglauca
Cissampelos madagascariensis
Antizoma angustifolia
Cissampelos capensis
Botryodiscia tetrandra
Spirospermum penduliflorum
Strychnopsis thouarsii
Rhaptonema sp.
Albertisia porcata
Anisocycla linearis
Triclisia dictyophylla
Tiliacora acuminata
Beirnaertia cabindensis
Carronia protensa
Pycnarrhena novoguineensis
Chondrodendron tomentosum
Curarea toxicofera
Cebatha pendula
Hyperbaena domingensis
Nephroia orbiculata
Pachygone valida
Cocculus hirsutus
Haematocarpus validus
Limacia blumei
Abuta grandifolia
Anomospermum chloranthum
Elissarrhena solimoesana
Sarcopetalum harveyanum
Hypserpa nitida
Parapachygone longifolia
Legnephora moorei
Pericampylus glaucus
Diploclisia affinis
Menispermum canadense
Sinomenium acutum
Cretaceous
Pal
Eocene
Oli
Miocene
P
100
90
80
70
60
50
40
30
20
10
0 Ma

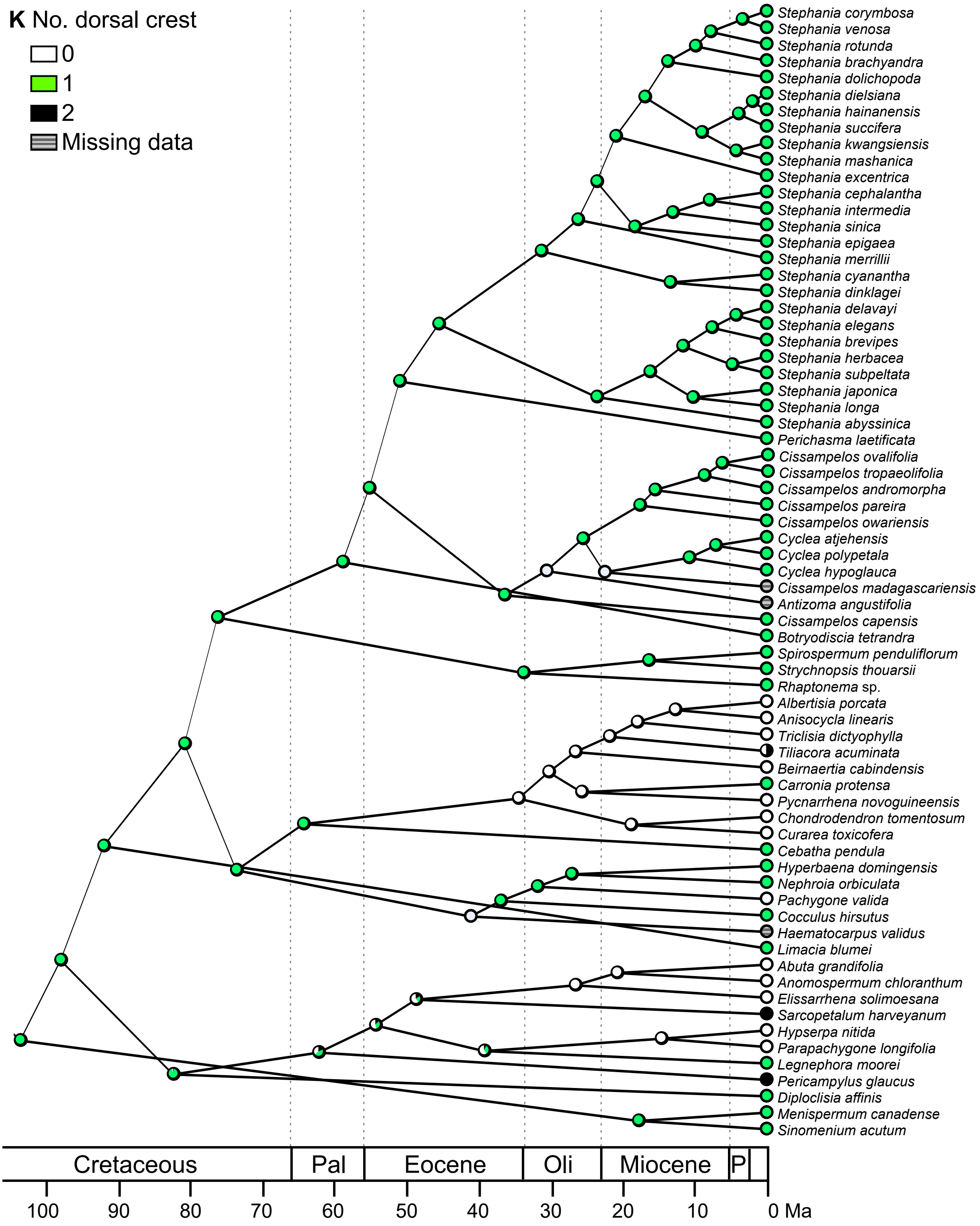
K No. dorsal crest
0
1
2
Missing data
Stephania corymbosa
Stephania venosa
Stephania rotunda
Stephania brachyandra
Stephania dolichopoda
Stephania dielsiana
Stephania hainanensis
Stephania succifera
Stephania kwangsiensis
Stephania mashanica
Stephania excentrica
Stephania cephalantha
Stephania intermedia
Stephania sinica
Stephania epigaea
Stephania merrillii
Stephania cyanantha
Stephania dinklagei
Stephania delavayi
Stephania elegans
Stephania brevipes
Stephania herbacea
Stephania subpeltata
Stephania japonica
Stephania longa
Stephania abyssinica
Perichasma laetificata
Cissampelos ovalifolia
Cissampelos tropaeolifolia
Cissampelos andromorpha
Cissampelos pareira
Cissampelos owariensis
Cyclea atjehensis
Cyclea polypetala
Cyclea hypoglauca
Cissampelos madagascariensis
Antizoma angustifolia
Cissampelos capensis
Botryodiscia tetrandra
Spirospermum penduliflorum
Strychnopsis thouarsii
Rhaptonema sp.
Albertisia porcata
Anisocycla linearis
Triclisia dictyophylla
Tiliacora acuminata
Beirnaertia cabindensis
Carronia protensa
Pycnarrhena novoguineensis
Chondrodendron tomentosum
Curarea toxicofera
Cebatha pendula
Hyperbaena domingensis
Nephroia orbiculata
Pachygone valida
Cocculus hirsutus
Haematocarpus validus
Limacia blumei
Abuta grandifolia
Anomospermum chloranthum
Elissarrhena solimoesana
Sarcopetalum harveyanum
Hypserpa nitida
Parapachygone longifolia
Legnephora moorei
Pericampylus glaucus
Diploclisia affinis
Menispermum canadense
Sinomenium acutum
Cretaceous
Pal
Eocene
Oli
Miocene
P
100
90
80
70
60
50
40
30
20
10
0 Ma

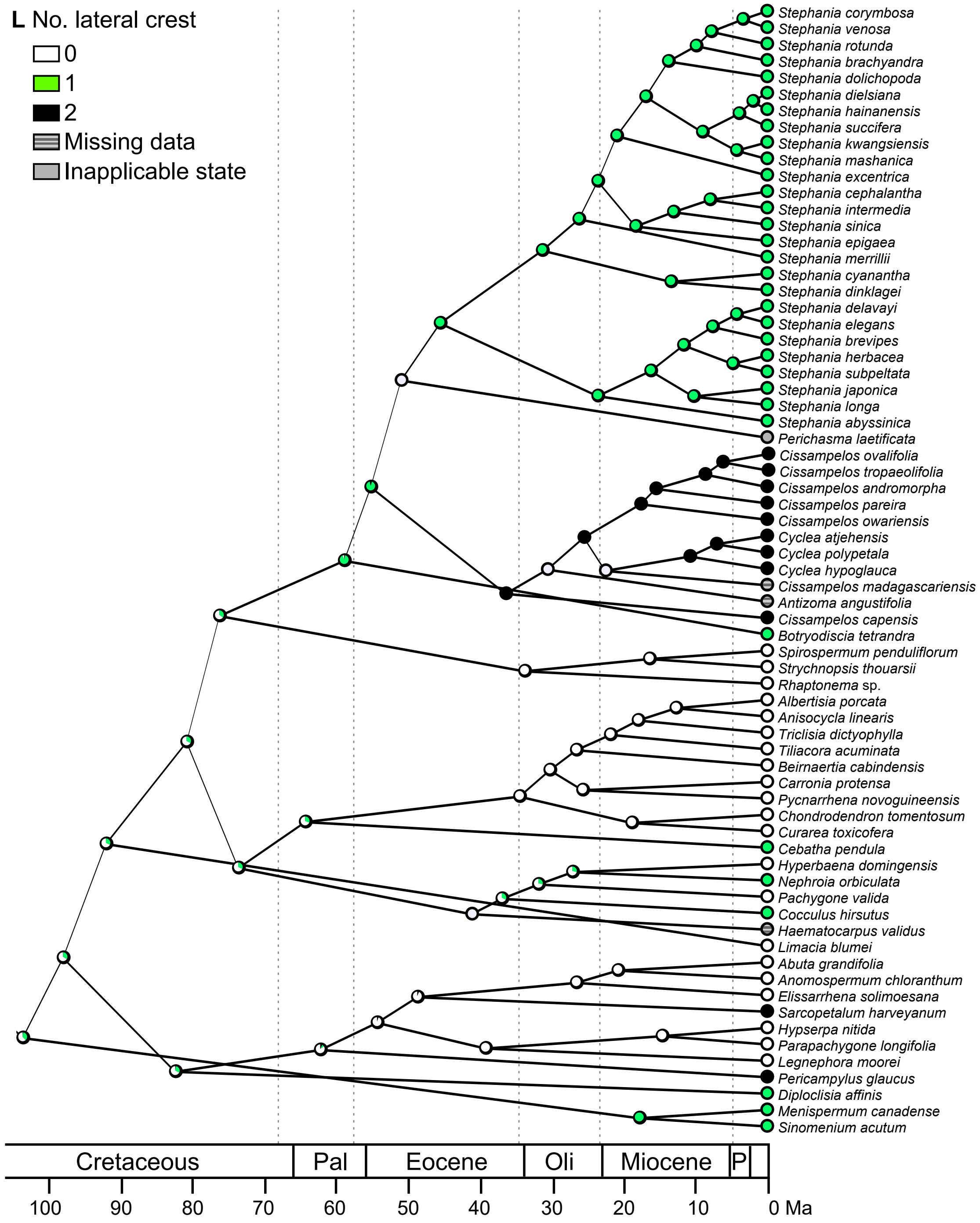

L No. lateral crest
0
1
2
Missing data
Inapplicable state
Stephania corymbosa
Stephania venosa
Stephania rotunda
Stephania brachyandra
Stephania dolichopoda
Stephania dielsiana
Stephania hainanensis
Stephania succifera
Stephania kwangsiensis
Stephania mashanica
Stephania excentrica
Stephania cephalantha
Stephania intermedia
Stephania sinica
Stephania epigaea
Stephania merrillii
Stephania cyanantha
Stephania dinklagei
Stephania delavayi
Stephania elegans
Stephania brevipes
Stephania herbacea
Stephania subpeltata
Stephania japonica
Stephania longa
Stephania abyssinica
Perichasma laetificata
Cissampelos ovalifolia
Cissampelos tropaeolifolia
Cissampelos andromorpha
Cissampelos pareira
Cissampelos owariensis
Cyclea atjehensis
Cyclea polypetala
Cyclea hypoglauca
Cissampelos madagascariensis
Antizoma angustifolia
Cissampelos capensis
Botryodiscia tetrandra
Spirospermum penduliflorum
Strychnopsis thouarsii
Rhaptonema sp.
Albertisia porcata
Anisocycla linearis
Triclisia dictyophylla
Tiliacora acuminata
Beirnaertia cabindensis
Carronia protensa
Pycnarrhena novoguineensis
Chondrodendron tomentosum
Curarea toxicofera
Cebatha pendula
Hyperbaena domingensis
Nephroia orbiculata
Pachygone valida
Cocculus hirsutus
Haematocarpus validus
Limacia blumei
Abuta grandifolia
Anomospermum chloranthum
Elissarrhena solimoesana
Sarcopetalum harveyanum
Hypserpa nitida
Parapachygone longifolia
Legnephora moorei
Pericampylus glaucus
Diploclisia affinis
Menispermum canadense
Sinomenium acutum
Cretaceous
Pal
Eocene
Oli
Miocene
P
100
90
80
70
60
50
40
30
20
10
0 Ma

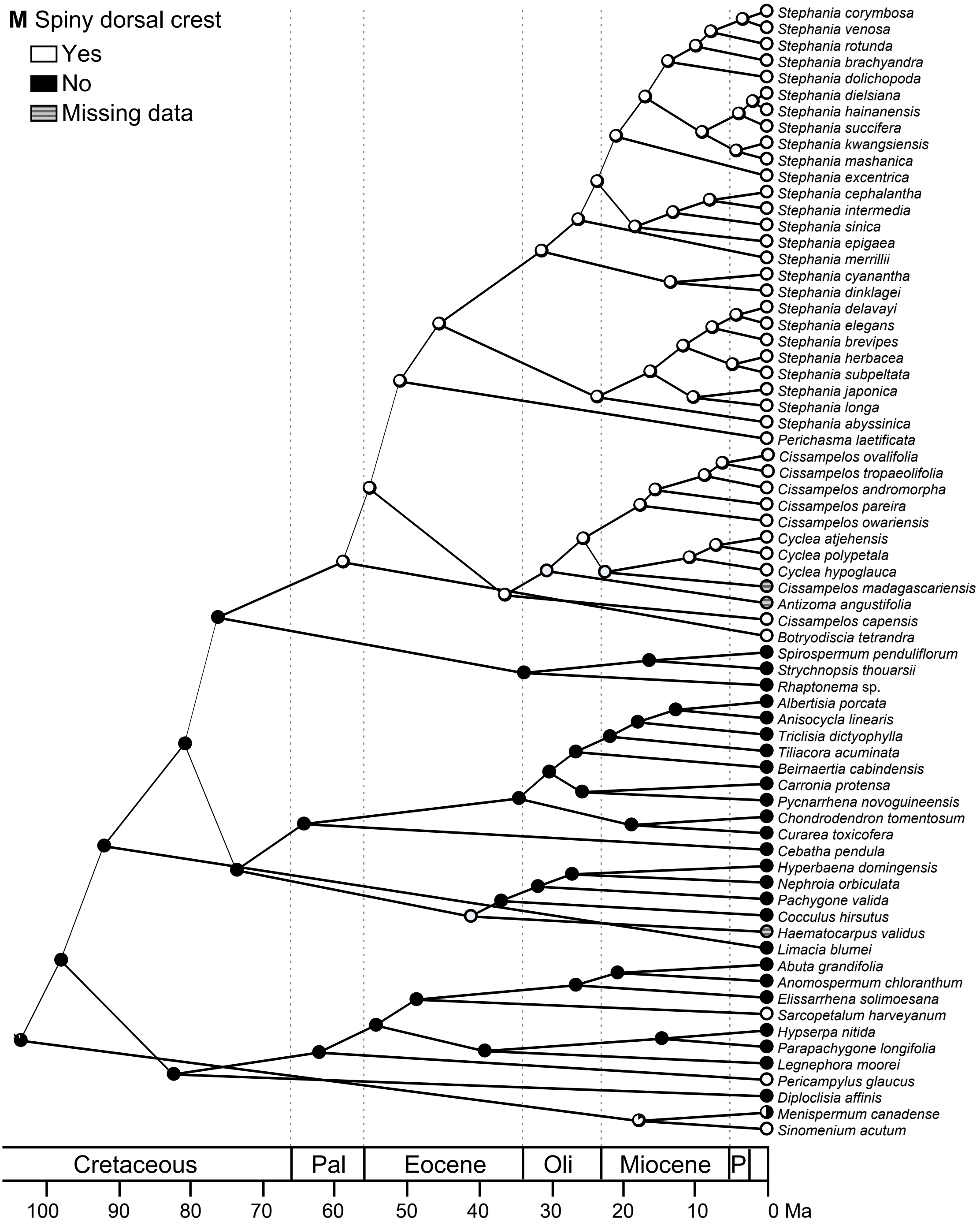
M Spiny dorsal crest
Yes
No
Missing data
Stephania corymbosa
Stephania venosa
Stephania rotunda
Stephania brachyandra
Stephania dolichopoda
Stephania dielsiana
Stephania hainanensis
Stephania succifera
Stephania kwangsiensis
Stephania mashanica
Stephania excentrica
Stephania cephalantha
Stephania intermedia
Stephania sinica
Stephania epigaea
Stephania merrillii
Stephania cyanantha
Stephania dinklagei
Stephania delavayi
Stephania elegans
Stephania brevipes
Stephania herbacea
Stephania subpeltata
Stephania japonica
Stephania longa
Stephania abyssinica
Perichasma laetificata
Cissampelos ovalifolia
Cissampelos tropaeolifolia
Cissampelos andromorpha
Cissampelos pareira
Cissampelos owariensis
Cyclea atjehensis
Cyclea polypetala
Cyclea hypoglauca
Cissampelos madagascariensis
Antizoma angustifolia
Cissampelos capensis
Botryodiscia tetrandra
Spirospermum penduliflorum
Strychnopsis thouarsii
Rhaptonema sp.
Albertisia porcata
Anisocycla linearis
Triclisia dictyophylla
Tiliacora acuminata
Beirnaertia cabindensis
Carronia protensa
Pycnarrhena novoguineensis
Chondrodendron tomentosum
Curarea toxicofera
Cebatha pendula
Hyperbaena domingensis
Nephroia orbiculata
Pachygone valida
Cocculus hirsutus
Haematocarpus validus
Limacia blumei
Abuta grandifolia
Anomospermum chloranthum
Elissarrhena solimoesana
Sarcopetalum harveyanum
Hypserpa nitida
Parapachygone longifolia
Legnephora moorei
Pericampylus glaucus
Diploclisia affinis
Menispermum canadense
Sinomenium acutum
Cretaceous
Pal
Eocene
Oli
Miocene
P
100
90
80
70
60
50
40
30
20
10
0 Ma

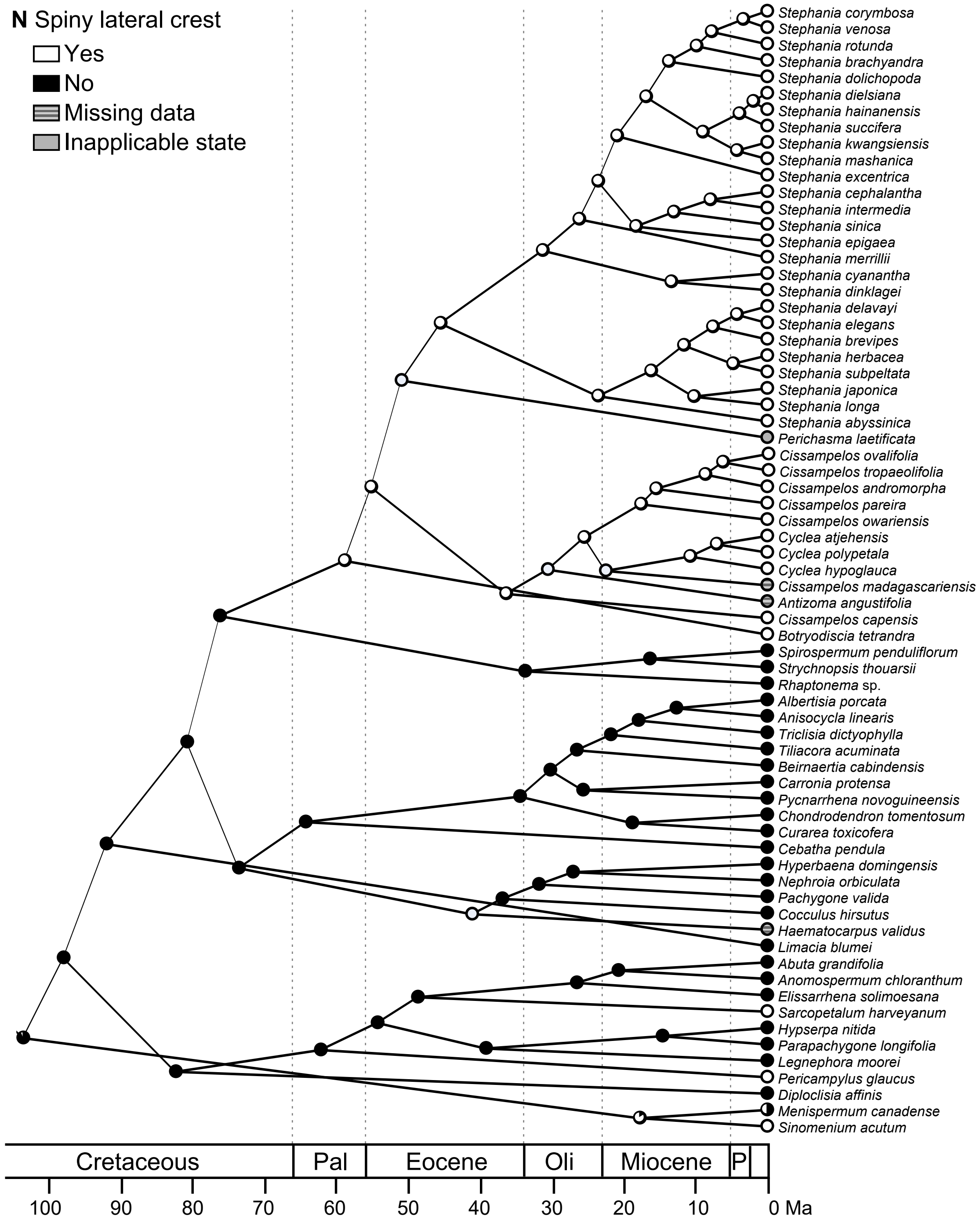
N Spiny lateral crest
Yes
No
Missing data
Inapplicable state
Stephania corymbosa
Stephania venosa
Stephania rotunda
Stephania brachyandra
Stephania dolichopoda
Stephania dielsiana
Stephania hainanensis
Stephania succifera
Stephania kwangsiensis
Stephania mashanica
Stephania excentrica
Stephania cephalantha
Stephania intermedia
Stephania sinica
Stephania epigaea
Stephania merrillii
Stephania cyanantha
Stephania dinklagei
Stephania delavayi
Stephania elegans
Stephania brevipes
Stephania herbacea
Stephania subpeltata
Stephania japonica
Stephania longa
Stephania abyssinica
Perichasma laetificata
Cissampelos ovalifolia
Cissampelos tropaeolifolia
Cissampelos andromorpha
Cissampelos pareira
Cissampelos owariensis
Cyclea atjehensis
Cyclea polypetala
Cyclea hypoglauca
Cissampelos madagascariensis
Antizoma angustifolia
Cissampelos capensis
Botryodiscia tetrandra
Spirospermum penduliflorum
Strychnopsis thouarsii
Rhaptonema sp.
Albertisia porcata
Anisocycla linearis
Triclisia dictyophylla
Tiliacora acuminata
Beirnaertia cabindensis
Carronia protensa
Pycnarrhena novoguineensis
Chondrodendron tomentosum
Curarea toxicofera
Cebatha pendula
Hyperbaena domingensis
Nephroia orbiculata
Pachygone valida
Cocculus hirsutus
Haematocarpus validus
Limacia blumei
Abuta grandifolia
Anomospermum chloranthum
Elissarrhena solimoesana
Sarcopetalum harveyanum
Hypserpa nitida
Parapachygone longifolia
Legnephora moorei
Pericampylus glaucus
Diploclisia affinis
Menispermum canadense
Sinomenium acutum
Cretaceous
Pal
Eocene
Oli
Miocene
P
100
90
80
70
60
50
40
30
20
10
0 Ma

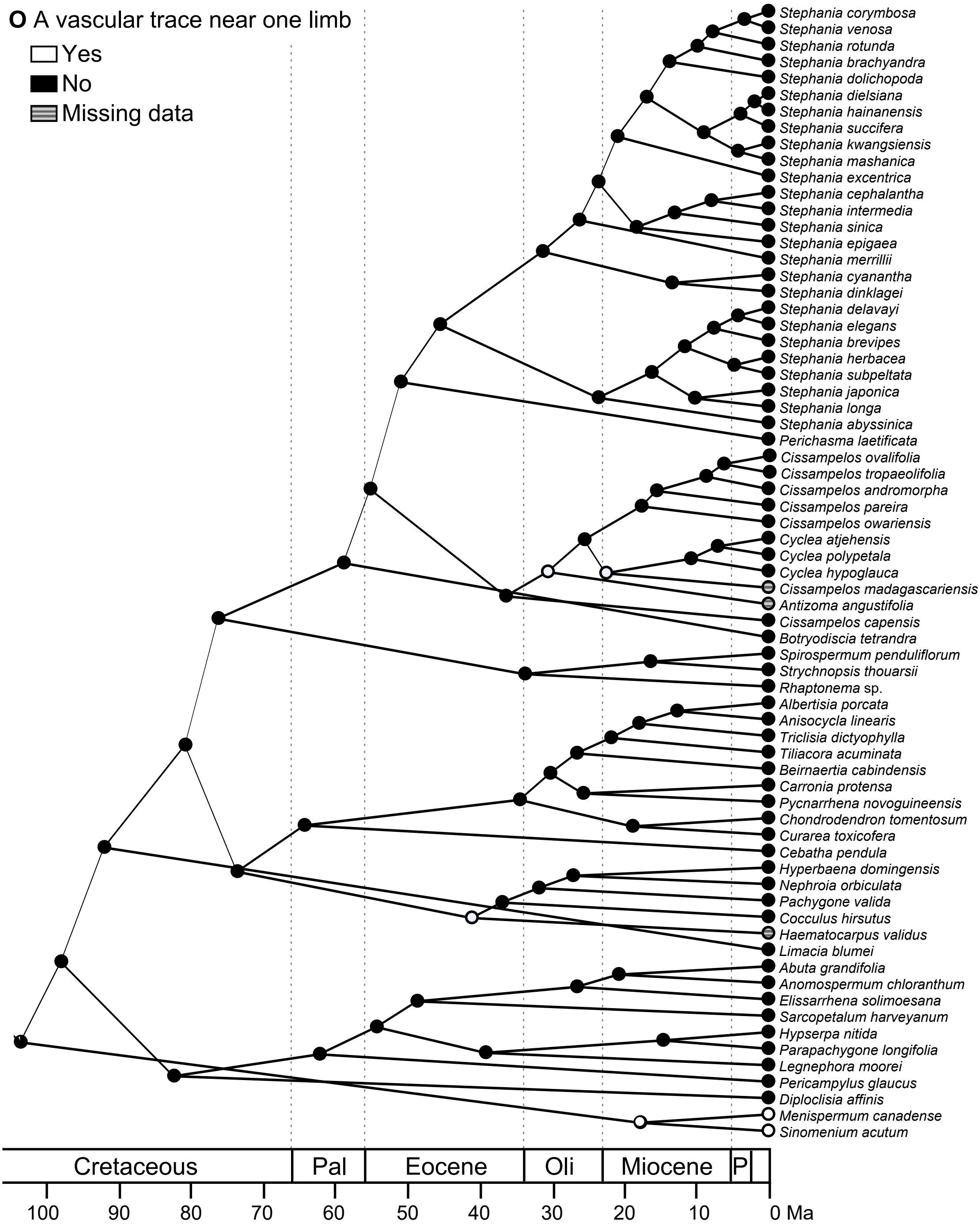
O A vascular trace near one limb
Yes
No
Missing data
Stephania corymbosa
Stephania venosa
Stephania rotunda
Stephania brachyandra
Stephania dolichopoda
Stephania dielsiana
Stephania hainanensis
Stephania succifera
Stephania kwangsiensis
Stephania mashanica
Stephania excentrica
Stephania cephalantha
Stephania intermedia
Stephania sinica
Stephania epigaea
Stephania merrillii
Stephania cyanantha
Stephania dinklagei
Stephania delavayi
Stephania elegans
Stephania brevipes
Stephania herbacea
Stephania subpeltata
Stephania japonica
Stephania longa
Stephania abyssinica
Perichasma laetificata
Cissampelos ovalifolia
Cissampelos tropaeolifolia
Cissampelos andromorpha
Cissampelos pareira
Cissampelos owariensis
Cyclea atjehensis
Cyclea polypetala
Cyclea hypoglauca
Cissampelos madagascariensis
Antizoma angustifolia
Cissampelos capensis
Botryodiscia tetrandra
Spirospermum penduliflorum
Strychnopsis thouarsii
Rhaptonema sp.
Albertisia porcata
Anisocycla linearis
Triclisia dictyophylla
Tiliacora acuminata
Beirnaertia cabindensis
Carronia protensa
Pycnarrhena novoguineensis
Chondrodendron tomentosum
Curarea toxicofera
Cebatha pendula
Hyperbaena domingensis
Nephroia orbiculata
Pachygone valida
Cocculus hirsutus
Haematocarpus validus
Limacia blumei
Abuta grandifolia
Anomospermum chloranthum
Elissarrhena solimoesana
Sarcopetalum harveyanum
Hypserpa nitida
Parapachygone longifolia
Legnephora moorei
Pericampylus glaucus
Diploclisia affinis
Menispermum canadense
Sinomenium acutum
Cretaceous
Pal
Eocene
Oli
Miocene
P
100
90
80
70
60
50
40
30
20
10
0 Ma

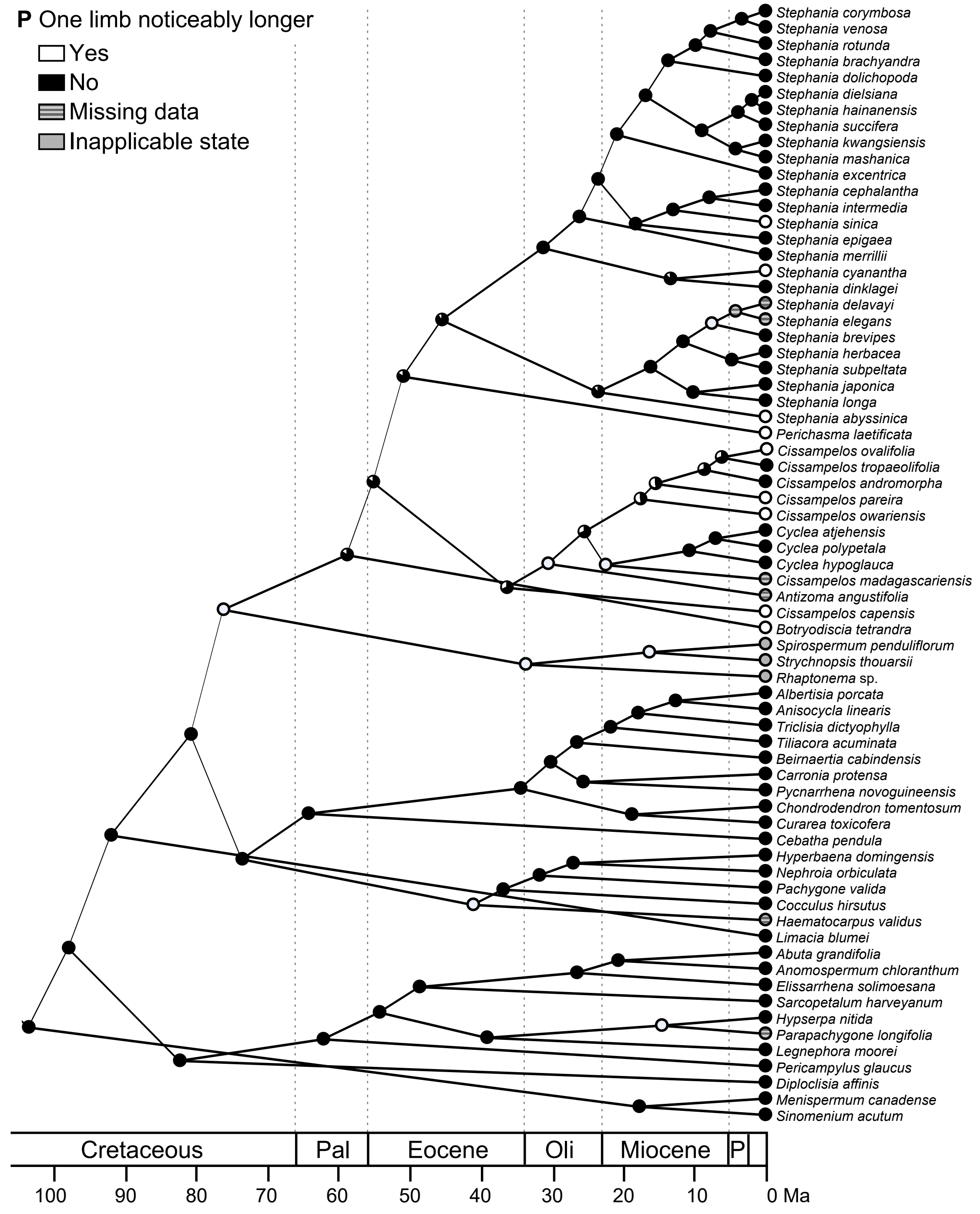

P One limb noticeably longer
Yes
No
Missing data
Inapplicable state
Stephania corymbosa
Stephania venosa
Stephania rotunda
Stephania brachyandra
Stephania dolichopoda
Stephania dielsiana
Stephania hainanensis
Stephania succifera
Stephania kwangsiensis
Stephania mashanica
Stephania excentrica
Stephania cephalantha
Stephania intermedia
Stephania sinica
Stephania epigaea
Stephania merrillii
Stephania cyanantha
Stephania dinklagei
Stephania delavayi
Stephania elegans
Stephania brevipes
Stephania herbacea
Stephania subpeltata
Stephania japonica
Stephania longa
Stephania abyssinica
Perichasma laetificata
Cissampelos ovalifolia
Cissampelos tropaeolifolia
Cissampelos andromorpha
Cissampelos pareira
Cissampelos owariensis
Cyclea atjehensis
Cyclea polypetala
Cyclea hypoglauca
Cissampelos madagascariensis
Antizoma angustifolia
Cissampelos capensis
Botryodiscia tetrandra
Spirospermum penduliflorum
Strychnopsis thouarsii
Rhaptonema sp.
Albertisia porcata
Anisocycla linearis
Triclisia dictyophylla
Tiliacora acuminata
Beirnaertia cabindensis
Carronia protensa
Pycnarrhena novoguineensis
Chondrodendron tomentosum
Curarea toxicofera
Cebatha pendula
Hyperbaena domingensis
Nephroia orbiculata
Pachygone valida
Cocculus hirsutus
Haematocarpus validus
Limacia blumei
Abuta grandifolia
Anomospermum chloranthum
Elissarrhena solimoesana
Sarcopetalum harveyanum
Hypserpa nitida
Parapachygone longifolia
Legnephora moorei
Pericampylus glaucus
Diploclisia affinis
Menispermum canadense
Sinomenium acutum
Cretaceous
Pal
Eocene
Oli
Miocene
P
100
90
80
70
60
50
40
30
20
10
0 Ma

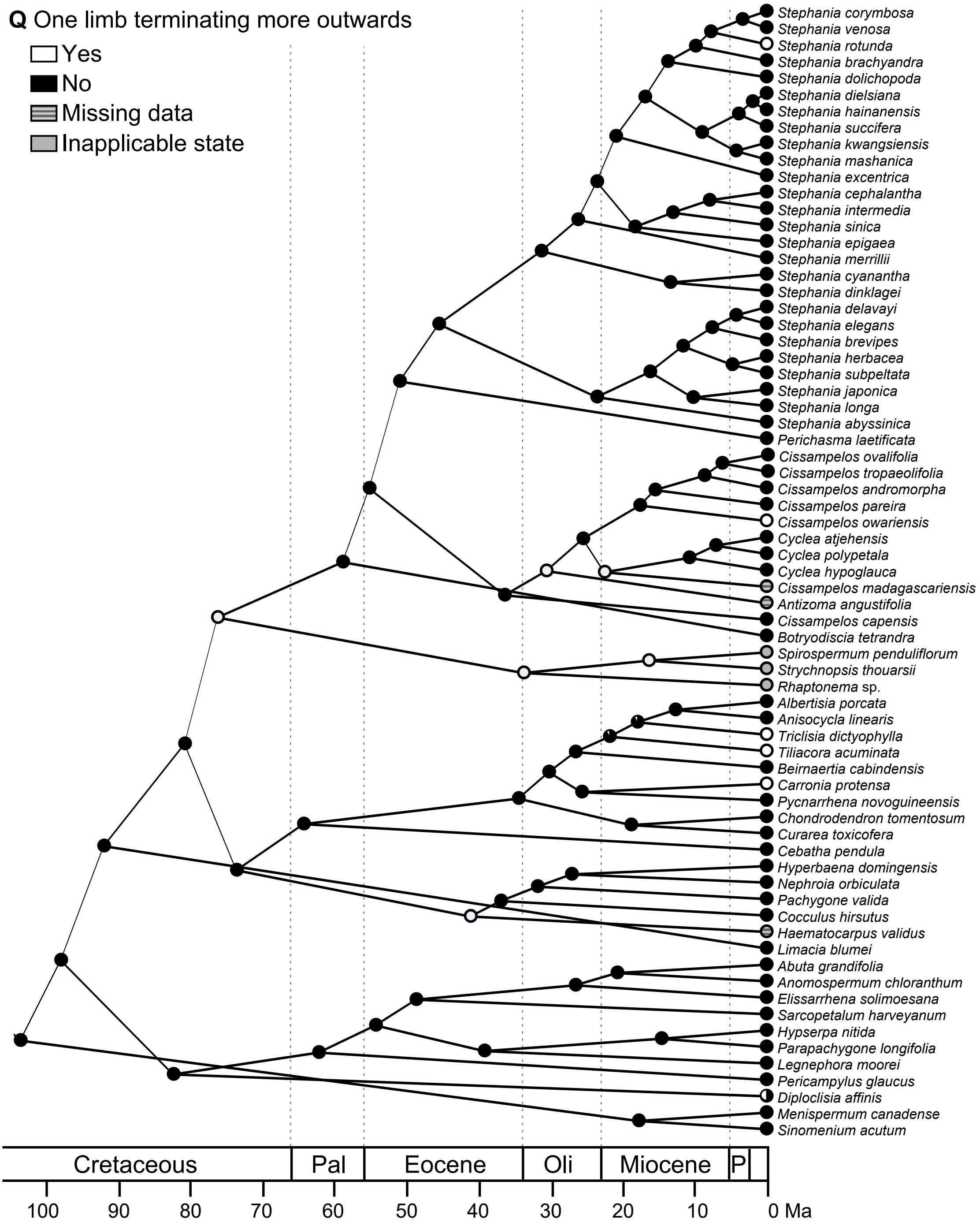
Q One limb terminating more outwards
Yes
No
Missing data
Inapplicable state
Stephania corymbosa
Stephania venosa
Stephania rotunda
Stephania brachyandra
Stephania dolichopoda
Stephania dielsiana
Stephania hainanensis
Stephania succifera
Stephania kwangsiensis
Stephania mashanica
Stephania excentrica
Stephania cephalantha
Stephania intermedia
Stephania sinica
Stephania epigaea
Stephania merrillii
Stephania cyanantha
Stephania dinklagei
Stephania delavayi
Stephania elegans
Stephania brevipes
Stephania herbacea
Stephania subpeltata
Stephania japonica
Stephania longa
Stephania abyssinica
Perichasma laetificata
Cissampelos ovalifolia
Cissampelos tropaeolifolia
Cissampelos andromorpha
Cissampelos pareira
Cissampelos owariensis
Cyclea atjehensis
Cyclea polypetala
Cyclea hypoglauca
Cissampelos madagascariensis
Antizoma angustifolia
Cissampelos capensis
Botryodiscia tetrandra
Spirospermum penduliflorum
Strychnopsis thouarsii
Rhaptonema sp.
Albertisia porcata
Anisocycla linearis
Triclisia dictyophylla
Tiliacora acuminata
Beirnaertia cabindensis
Carronia protensa
Pycnarrhena novoguineensis
Chondrodendron tomentosum
Curarea toxicofera
Cebatha pendula
Hyperbaena domingensis
Nephroia orbiculata
Pachygone valida
Cocculus hirsutus
Haematocarpus validus
Limacia blumei
Abuta grandifolia
Anomospermum chloranthum
Elissarrhena solimoesana
Sarcopetalum harveyanum
Hypserpa nitida
Parapachygone longifolia
Legnephora moorei
Pericampylus glaucus
Diploclisia affinis
Menispermum canadense
Sinomenium acutum
Cretaceous
Pal
Eocene
Oli
Miocene
P
100
90
80
70
60
50
40
30
20
10
0 Ma

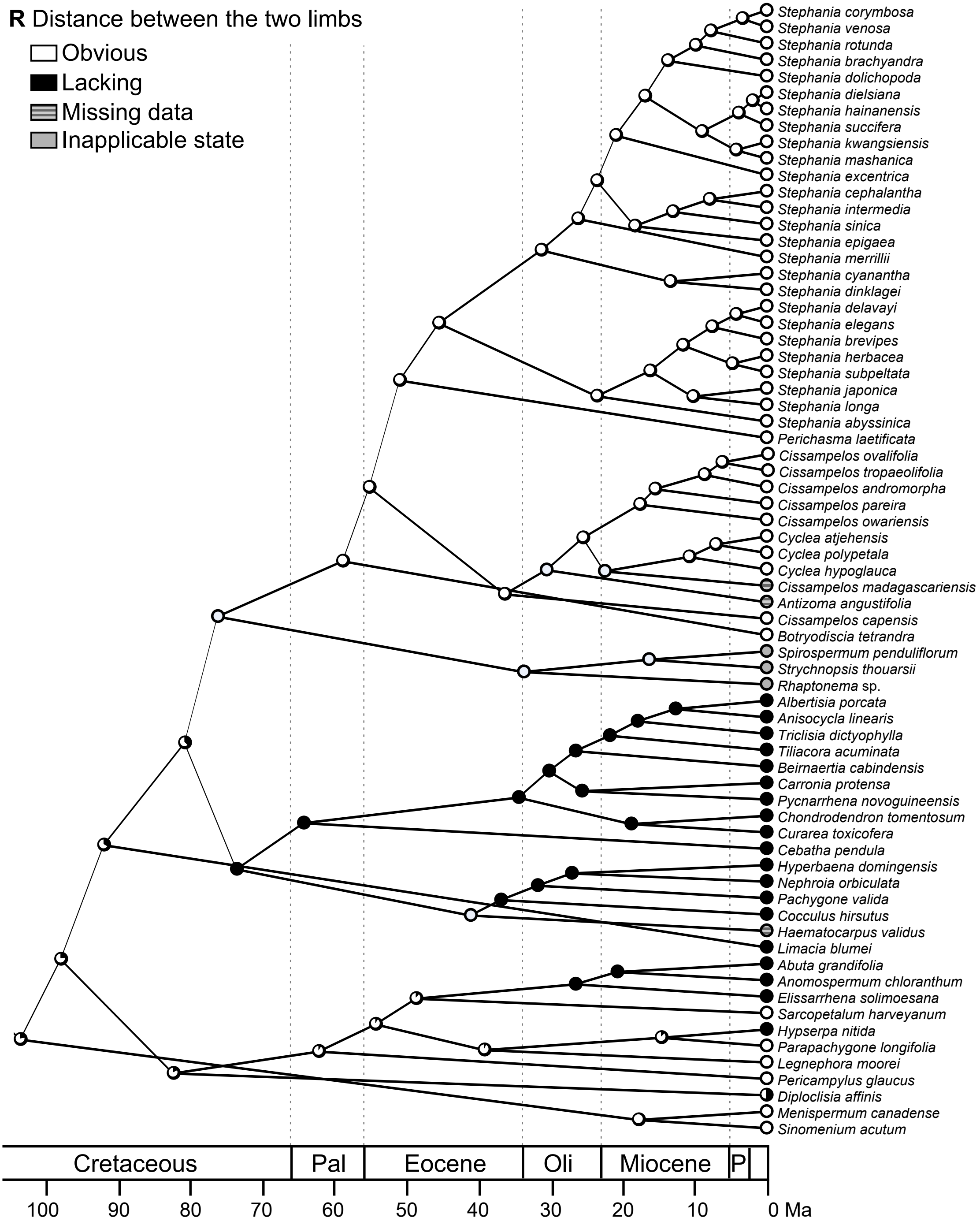
R Distance between the two limbs
Obvious
Lacking
Missing data
Inapplicable state
Stephania corymbosa
Stephania venosa
Stephania rotunda
Stephania brachyandra
Stephania dolichopoda
Stephania dielsiana
Stephania hainanensis
Stephania succifera
Stephania kwangsiensis
Stephania mashanica
Stephania excentrica
Stephania cephalantha
Stephania intermedia
Stephania sinica
Stephania epigaea
Stephania merrillii
Stephania cyanantha
Stephania dinklagei
Stephania delavayi
Stephania elegans
Stephania brevipes
Stephania herbacea
Stephania subpeltata
Stephania japonica
Stephania longa
Stephania abyssinica
Perichasma laetificata
Cissampelos ovalifolia
Cissampelos tropaeolifolia
Cissampelos andromorpha
Cissampelos pareira
Cissampelos owariensis
Cyclea atjehensis
Cyclea polypetala
Cyclea hypoglauca
Cissampelos madagascariensis
Antizoma angustifolia
Cissampelos capensis
Botryodiscia tetrandra
Spirospermum penduliflorum
Strychnopsis thouarsii
Rhaptonema sp.
Albertisia porcata
Anisocycla linearis
Triclisia dictyophylla
Tiliacora acuminata
Beirnaertia cabindensis
Carronia protensa
Pycnarrhena novoguineensis
Chondrodendron tomentosum
Curarea toxicofera
Cebatha pendula
Hyperbaena domingensis
Nephroia orbiculata
Pachygone valida
Cocculus hirsutus
Haematocarpus validus
Limacia blumei
Abuta grandifolia
Anomospermum chloranthum
Elissarrhena solimoesana
Sarcopetalum harveyanum
Hypserpa nitida
Parapachygone longifolia
Legnephora moorei
Pericampylus glaucus
Diploclisia affinis
Menispermum canadense
Sinomenium acutum
Cretaceous
Pal
Eocene
Oli
Miocene
P
100
90
80
70
60
50
40
30
20
10
0 Ma

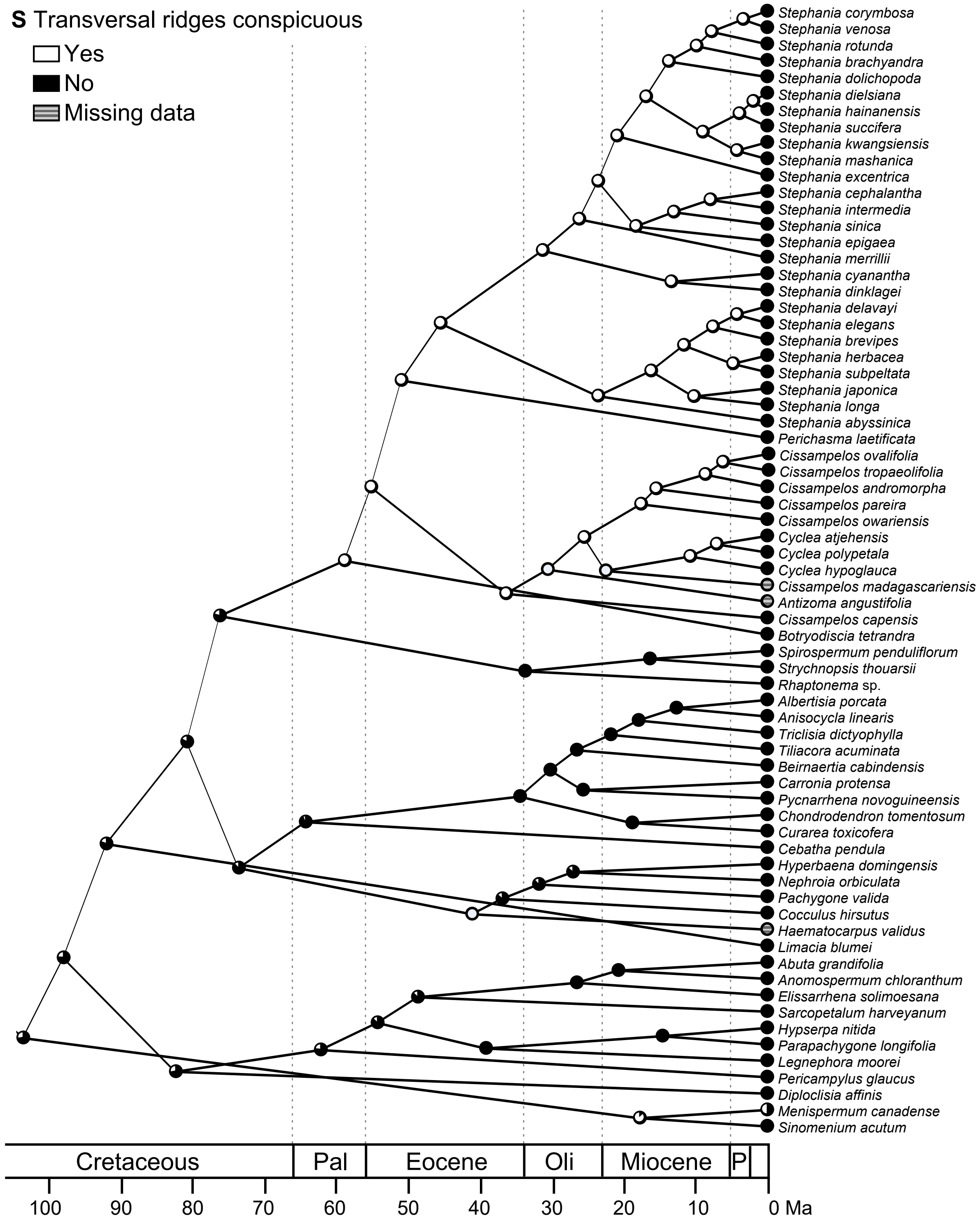
S Transversal ridges conspicuous
Yes
No
Missing data
Stephania corymbosa
Stephania venosa
Stephania rotunda
Stephania brachyandra
Stephania dolichopoda
Stephania dielsiana
Stephania hainanensis
Stephania succifera
Stephania kwangsiensis
Stephania mashanica
Stephania excentrica
Stephania cephalantha
Stephania intermedia
Stephania sinica
Stephania epigaea
Stephania merrillii
Stephania cyanantha
Stephania dinklagei
Stephania delavayi
Stephania elegans
Stephania brevipes
Stephania herbacea
Stephania subpeltata
Stephania japonica
Stephania longa
Stephania abyssinica
Perichasma laetificata
Cissampelos ovalifolia
Cissampelos tropaeolifolia
Cissampelos andromorpha
Cissampelos pareira
Cissampelos owariensis
Cyclea atjehensis
Cyclea polypetala
Cyclea hypoglauca
Cissampelos madagascariensis
Antizoma angustifolia
Cissampelos capensis
Botryodiscia tetrandra
Spirospermum penduliflorum
Strychnopsis thouarsii
Rhaptonema sp.
Albertisia porcata
Anisocycla linearis
Triclisia dictyophylla
Tiliacora acuminata
Beirnaertia cabindensis
Carronia protensa
Pycnarrhena novoguineensis
Chondrodendron tomentosum
Curarea toxicofera
Cebatha pendula
Hyperbaena domingensis
Nephroia orbiculata
Pachygone valida
Cocculus hirsutus
Haematocarpus validus
Limacia blumei
Abuta grandifolia
Anomospermum chloranthum
Elissarrhena solimoesana
Sarcopetalum harveyanum
Hypserpa nitida
Parapachygone longifolia
Legnephora moorei
Pericampylus glaucus
Diploclisia affinis
Menispermum canadense
Sinomenium acutum
Cretaceous
Pal
Eocene
Oli
Miocene
P
100
90
80
70
60
50
40
30
20
10
0 Ma

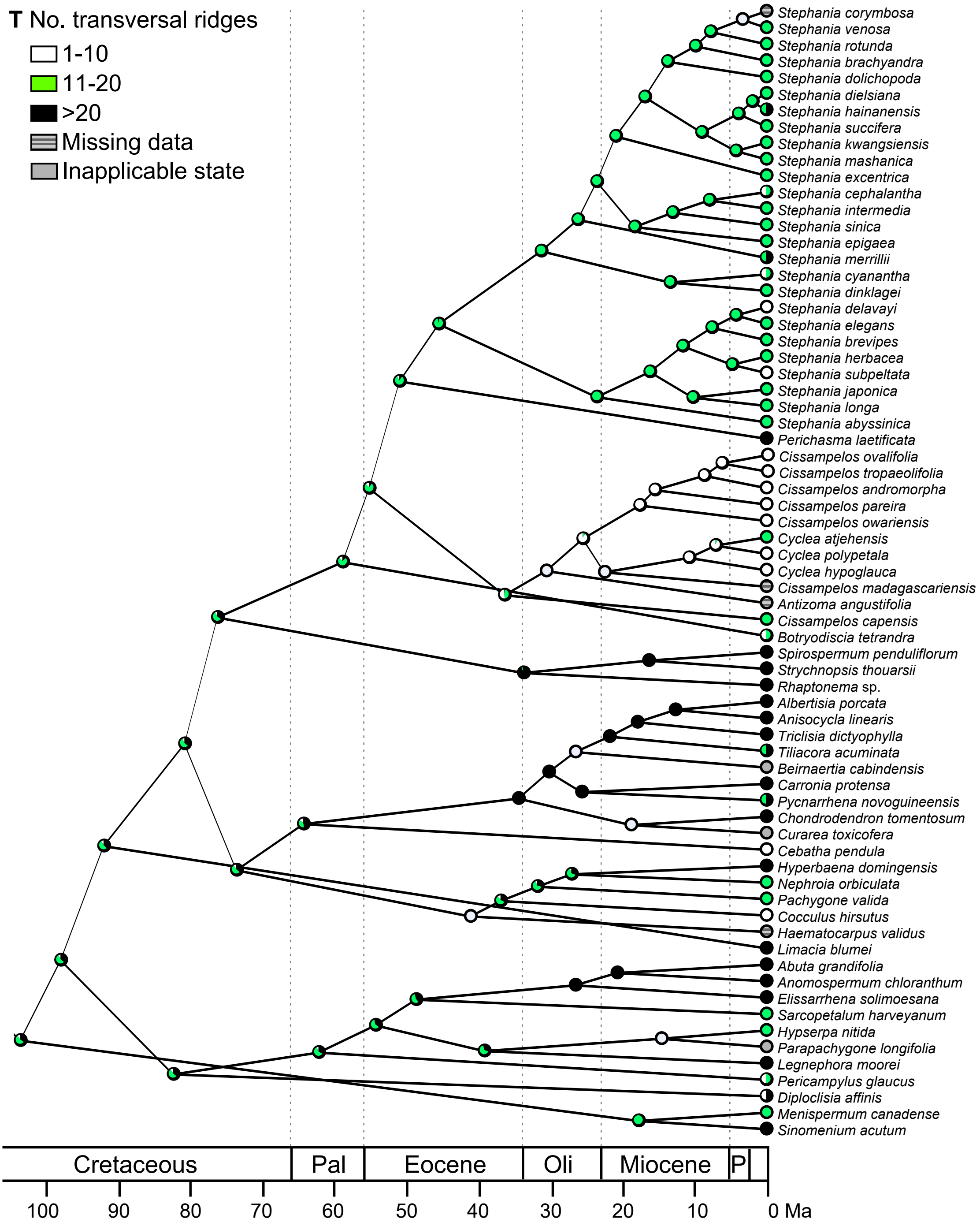
T No. transversal ridges
1-10
11-20
>20
Missing data
Inapplicable state
Stephania corymbosa
Stephania venosa
Stephania rotunda
Stephania brachyandra
Stephania dolichopoda
Stephania dielsiana
Stephania hainanensis
Stephania succifera
Stephania kwangsiensis
Stephania mashanica
Stephania excentrica
Stephania cephalantha
Stephania intermedia
Stephania sinica
Stephania epigaea
Stephania merrillii
Stephania cyanantha
Stephania dinklagei
Stephania delavayi
Stephania elegans
Stephania brevipes
Stephania herbacea
Stephania subpeltata
Stephania japonica
Stephania longa
Stephania abyssinica
Perichasma laetificata
Cissampelos ovalifolia
Cissampelos tropaeolifolia
Cissampelos andromorpha
Cissampelos pareira
Cissampelos owariensis
Cyclea atjehensis
Cyclea polypetala
Cyclea hypoglauca
Cissampelos madagascariensis
Antizoma angustifolia
Cissampelos capensis
Botryodiscia tetrandra
Spirospermum penduliflorum
Strychnopsis thouarsii
Rhaptonema sp.
Albertisia porcata
Anisocycla linearis
Triclisia dictyophylla
Tiliacora acuminata
Beirnaertia cabindensis
Carronia protensa
Pycnarrhena novoguineensis
Chondrodendron tomentosum
Curarea toxicofera
Cebatha pendula
Hyperbaena domingensis
Nephroia orbiculata
Pachygone valida
Cocculus hirsutus
Haematocarpus validus
Limacia blumei
Abuta grandifolia
Anomospermum chloranthum
Elissarrhena solimoesana
Sarcopetalum harveyanum
Hypserpa nitida
Parapachygone longifolia
Legnephora moorei
Pericampylus glaucus
Diploclisia affinis
Menispermum canadense
Sinomenium acutum
Cretaceous
Pal
Eocene
Oli
Miocene
P
100
90
80
70
60
50
40
30
20
10
0 Ma

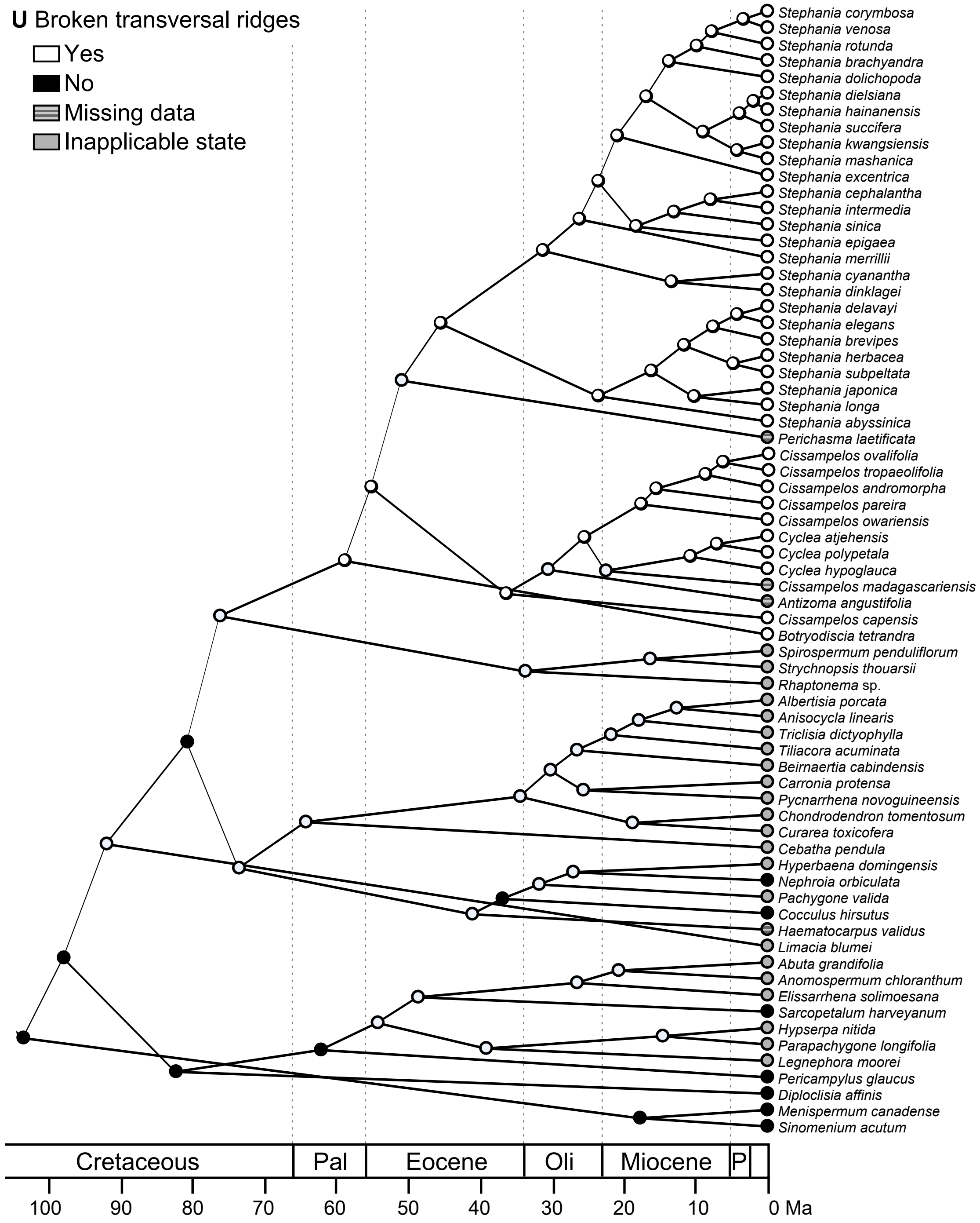
U Broken transversal ridges
Yes
No
Missing data
Inapplicable state
Stephania corymbosa
Stephania venosa
Stephania rotunda
Stephania brachyandra
Stephania dolichopoda
Stephania dielsiana
Stephania hainanensis
Stephania succifera
Stephania kwangsiensis
Stephania mashanica
Stephania excentrica
Stephania cephalantha
Stephania intermedia
Stephania sinica
Stephania epigaea
Stephania merrillii
Stephania cyanantha
Stephania dinklagei
Stephania delavayi
Stephania elegans
Stephania brevipes
Stephania herbacea
Stephania subpeltata
Stephania japonica
Stephania longa
Stephania abyssinica
Perichasma laetificata
Cissampelos ovalifolia
Cissampelos tropaeolifolia
Cissampelos andromorpha
Cissampelos pareira
Cissampelos owariensis
Cyclea atjehensis
Cyclea polypetala
Cyclea hypoglauca
Cissampelos madagascariensis
Antizoma angustifolia
Cissampelos capensis
Botryodiscia tetrandra
Spirospermum penduliflorum
Strychnopsis thouarsii
Rhaptonema sp.
Albertisia porcata
Anisocycla linearis
Triclisia dictyophylla
Tiliacora acuminata
Beirnaertia cabindensis
Carronia protensa
Pycnarrhena novoguineensis
Chondrodendron tomentosum
Curarea toxicofera
Cebatha pendula
Hyperbaena domingensis
Nephroia orbiculata
Pachygone valida
Cocculus hirsutus
Haematocarpus validus
Limacia blumei
Abuta grandifolia
Anomospermum chloranthum
Elissarrhena solimoesana
Sarcopetalum harveyanum
Hypserpa nitida
Parapachygone longifolia
Legnephora moorei
Pericampylus glaucus
Diploclisia affinis
Menispermum canadense
Sinomenium acutum
Cretaceous
Pal
Eocene
Oli
Miocene
P
100
90
80
70
60
50
40
30
20
10
0 Ma

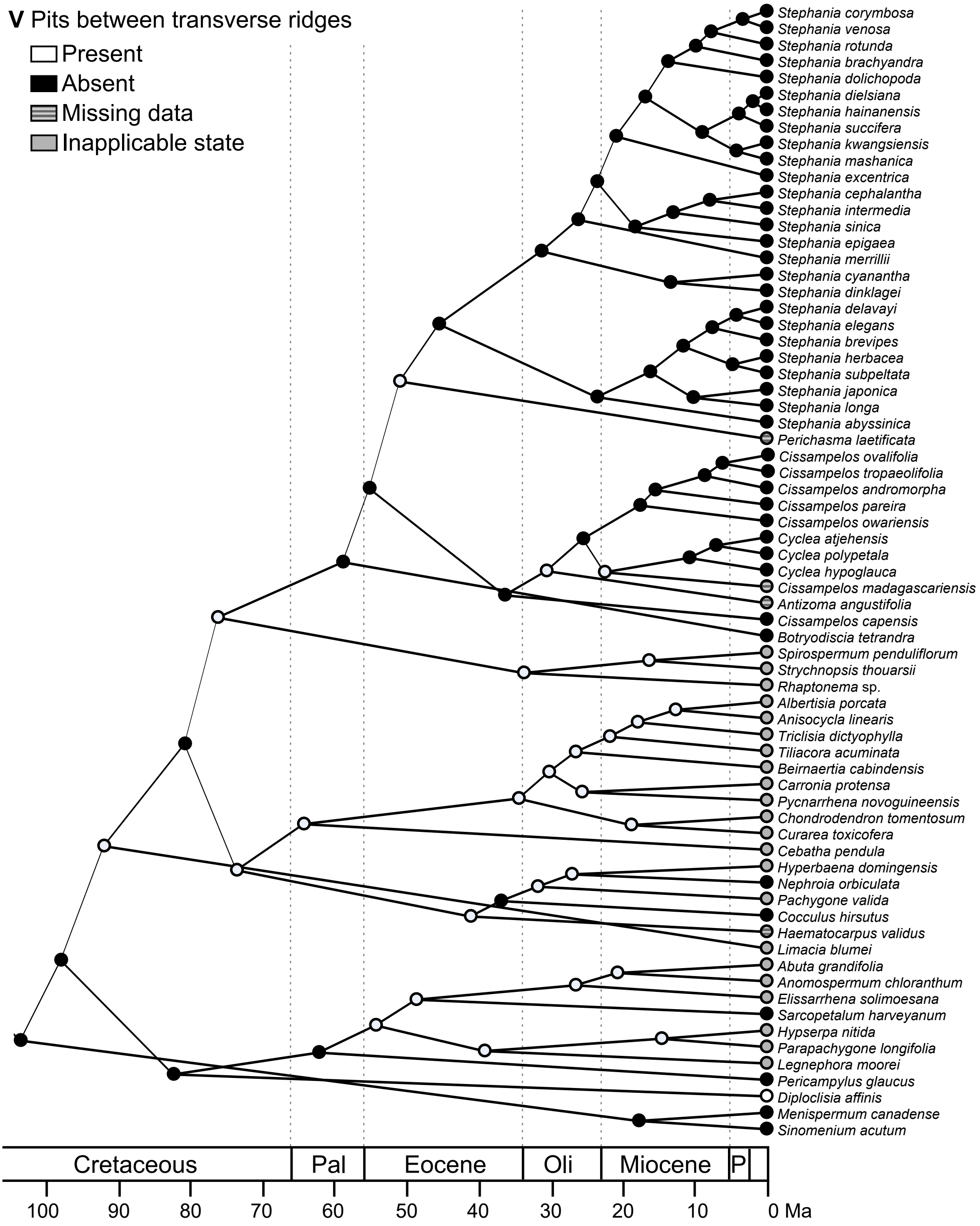

V Pits between transverse ridges
Present
Absent
Missing data
Inapplicable state
Stephania corymbosa
Stephania venosa
Stephania rotunda
Stephania brachyandra
Stephania dolichopoda
Stephania dielsiana
Stephania hainanensis
Stephania succifera
Stephania kwangsiensis
Stephania mashanica
Stephania excentrica
Stephania cephalantha
Stephania intermedia
Stephania sinica
Stephania epigaea
Stephania merrillii
Stephania cyanantha
Stephania dinklagei
Stephania delavayi
Stephania elegans
Stephania brevipes
Stephania herbacea
Stephania subpeltata
Stephania japonica
Stephania longa
Stephania abyssinica
Perichasma laetificata
Cissampelos ovalifolia
Cissampelos tropaeolifolia
Cissampelos andromorpha
Cissampelos pareira
Cissampelos owariensis
Cyclea atjehensis
Cyclea polypetala
Cyclea hypoglauca
Cissampelos madagascariensis
Antizoma angustifolia
Cissampelos capensis
Botryodiscia tetrandra
Spirospermum penduliflorum
Strychnopsis thouarsii
Rhaptonema sp.
Albertisia porcata
Anisocycla linearis
Triclisia dictyophylla
Tiliacora acuminata
Beirnaertia cabindensis
Carronia protensa
Pycnarrhena novoguineensis
Chondrodendron tomentosum
Curarea toxicofera
Cebatha pendula
Hyperbaena domingensis
Nephroia orbiculata
Pachygone valida
Cocculus hirsutus
Haematocarpus validus
Limacia blumei
Abuta grandifolia
Anomospermum chloranthum
Elissarrhena solimoesana
Sarcopetalum harveyanum
Hypserpa nitida
Parapachygone longifolia
Legnephora moorei
Pericampylus glaucus
Diploclisia affinis
Menispermum canadense
Sinomenium acutum
Cretaceous
Pal
Eocene
Oli
Miocene
P
100
90
80
70
60
50
40
30
20
10
0 Ma

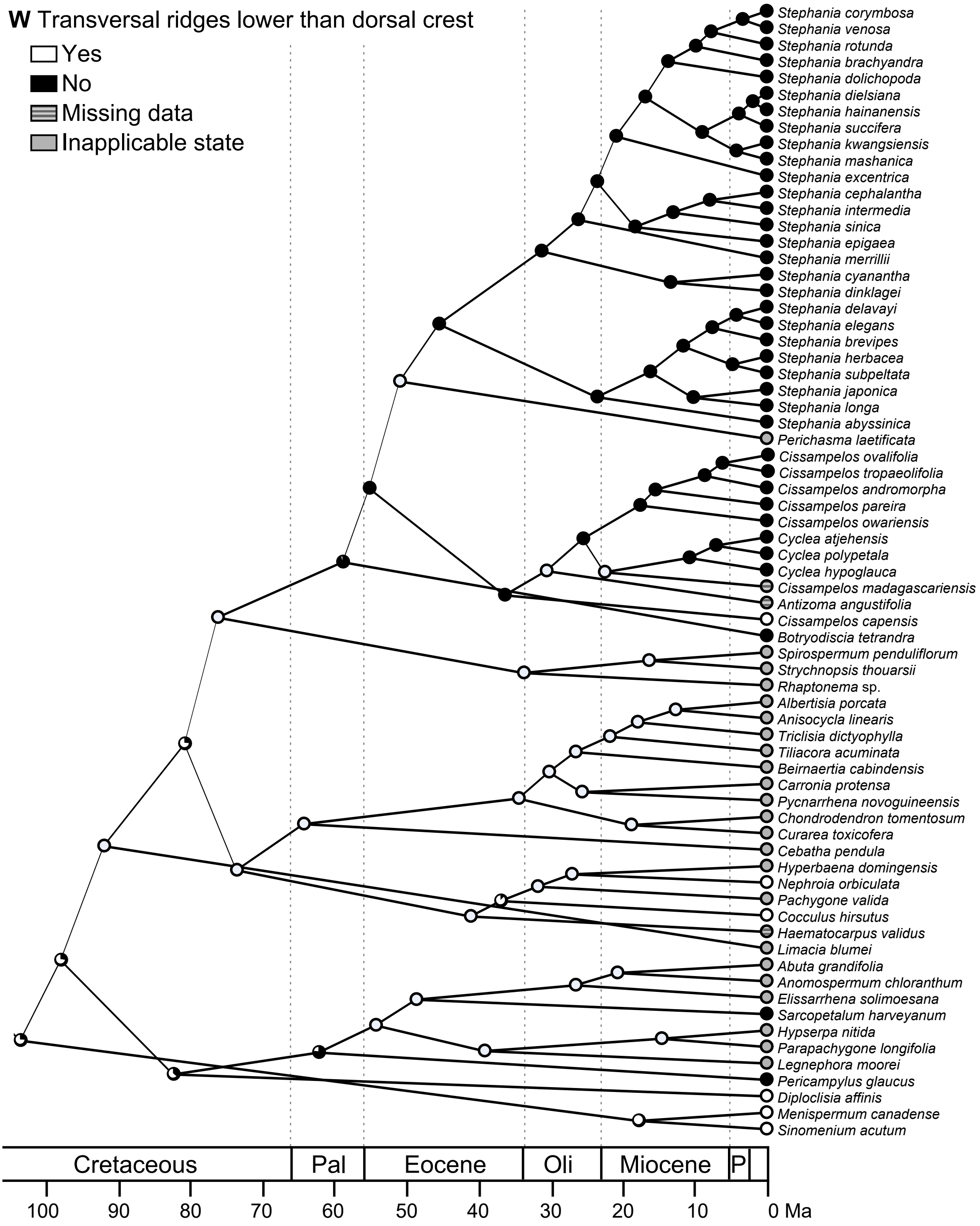
W Transversal ridges lower than dorsal crest
Yes
No
Missing data
Inapplicable state
Stephania corymbosa
Stephania venosa
Stephania rotunda
Stephania brachyandra
Stephania dolichopoda
Stephania dielsiana
Stephania hainanensis
Stephania succifera
Stephania kwangsiensis
Stephania mashanica
Stephania excentrica
Stephania cephalantha
Stephania intermedia
Stephania sinica
Stephania epigaea
Stephania merrillii
Stephania cyanantha
Stephania dinklagei
Stephania delavayi
Stephania elegans
Stephania brevipes
Stephania herbacea
Stephania subpeltata
Stephania japonica
Stephania longa
Stephania abyssinica
Perichasma laetificata
Cissampelos ovalifolia
Cissampelos tropaeolifolia
Cissampelos andromorpha
Cissampelos pareira
Cissampelos owariensis
Cyclea atjehensis
Cyclea polypetala
Cyclea hypoglauca
Cissampelos madagascariensis
Antizoma angustifolia
Cissampelos capensis
Botryodiscia tetrandra
Spirospermum penduliflorum
Strychnopsis thouarsii
Rhaptonema sp.
Albertisia porcata
Anisocycla linearis
Triclisia dictyophylla
Tiliacora acuminata
Beirnaertia cabindensis
Carronia protensa
Pycnarrhena novoguineensis
Chondrodendron tomentosum
Curarea toxicofera
Cebatha pendula
Hyperbaena domingensis
Nephroia orbiculata
Pachygone valida
Cocculus hirsutus
Haematocarpus validus
Limacia blumei
Abuta grandifolia
Anomospermum chloranthum
Elissarrhena solimoesana
Sarcopetalum harveyanum
Hypserpa nitida
Parapachygone longifolia
Legnephora moorei
Pericampylus glaucus
Diploclisia affinis
Menispermum canadense
Sinomenium acutum
Cretaceous
Pal
Eocene
Oli
Miocene
P
100
90
80
70
60
50
40
30
20
10
0 Ma

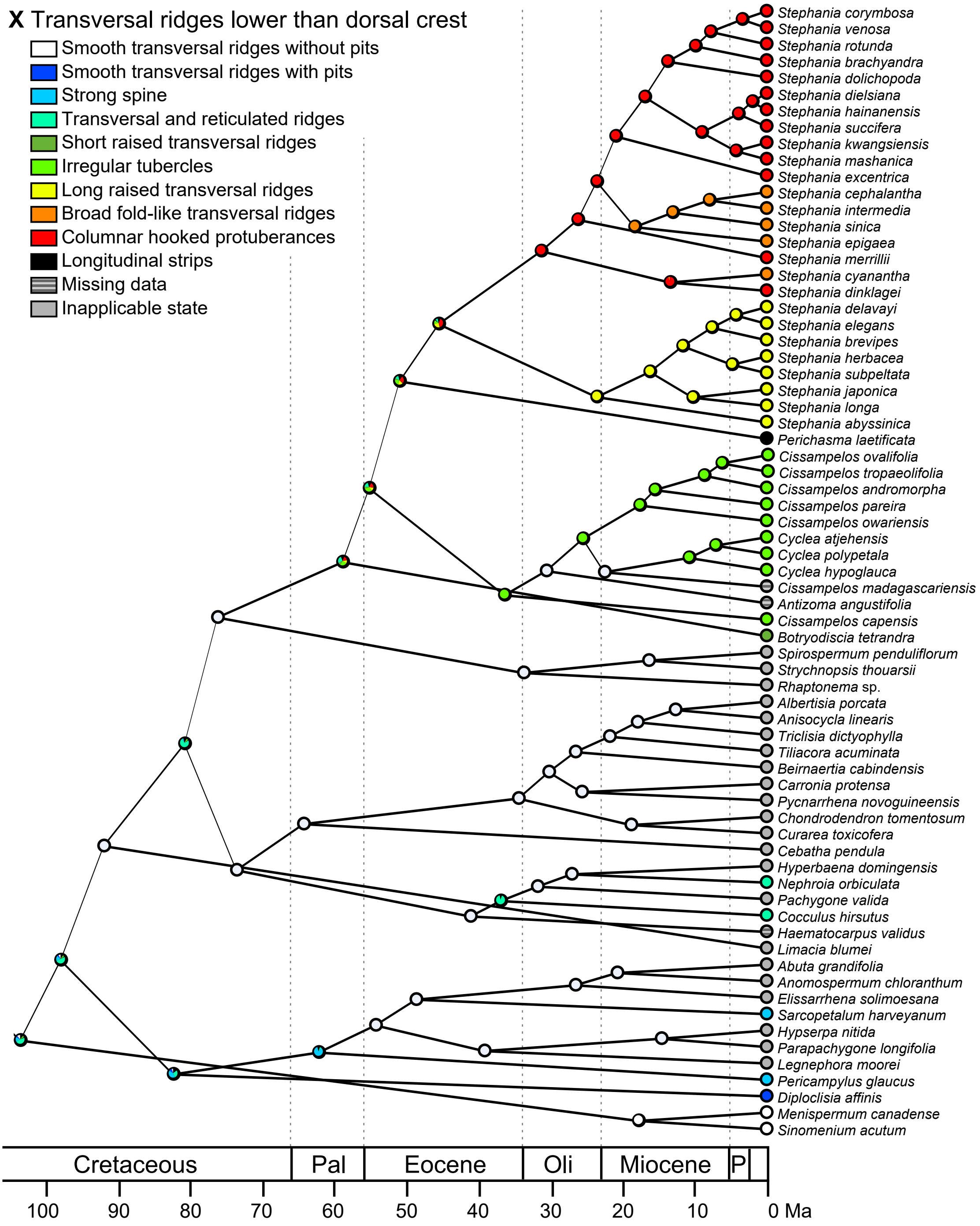
X Transversal ridges lower than dorsal crest
Smooth transversal ridges without pits
Smooth transversal ridges with pits
Strong spine
Transversal and reticulated ridges
Short raised transversal ridges
Irregular tubercles
Long raised transversal ridges
Broad fold-like transversal ridges
Columnar hooked protuberances
Longitudinal strips
Missing data
Inapplicable state
Stephania corymbosa
Stephania venosa
Stephania rotunda
Stephania brachyandra
Stephania dolichopoda
Stephania dielsiana
Stephania hainanensis
Stephania succifera
Stephania kwangsiensis
Stephania mashanica
Stephania excentrica
Stephania cephalantha
Stephania intermedia
Stephania sinica
Stephania epigaea
Stephania merrillii
Stephania cyanantha
Stephania dinklagei
Stephania delavayi
Stephania elegans
Stephania brevipes
Stephania herbacea
Stephania subpeltata
Stephania japonica
Stephania longa
Stephania abyssinica
Perichasma laetificata
Cissampelos ovalifolia
Cissampelos tropaeolifolia
Cissampelos andromorpha
Cissampelos pareira
Cissampelos owariensis
Cyclea atjehensis
Cyclea polypetala
Cyclea hypoglauca
Cissampelos madagascariensis
Antizoma angustifolia
Cissampelos capensis
Botryodiscia tetrandra
Spirospermum penduliflorum
Strychnopsis thouarsii
Rhaptonema sp.
Albertisia porcata
Anisocycla linearis
Triclisia dictyophylla
Tiliacora acuminata
Beirnaertia cabindensis
Carronia protensa
Pycnarrhena novoguineensis
Chondrodendron tomentosum
Curarea toxicofera
Cebatha pendula
Hyperbaena domingensis
Nephroia orbiculata
Pachygone valida
Cocculus hirsutus
Haematocarpus validus
Limacia blumei
Abuta grandifolia
Anomospermum chloranthum
Elissarrhena solimoesana
Sarcopetalum harveyanum
Hypserpa nitida
Parapachygone longifolia
Legnephora moorei
Pericampylus glaucus
Diploclisia affinis
Menispermum canadense
Sinomenium acutum
Cretaceous
Pal
Eocene
Oli
Miocene
P
100
90
80
70
60
50
40
30
20
10
0 Ma
