# Supplementary figures and images for: The endocarp evolution of Cissampelideae (Menispermaceae): integrating extant and fossil species
Source: Ann Bot. 2025 Oct 22;137(6):2015–24. doi: 10.1093/aob/mcaf240 (PMC13274978; doi:10.1093/aob/mcaf240)

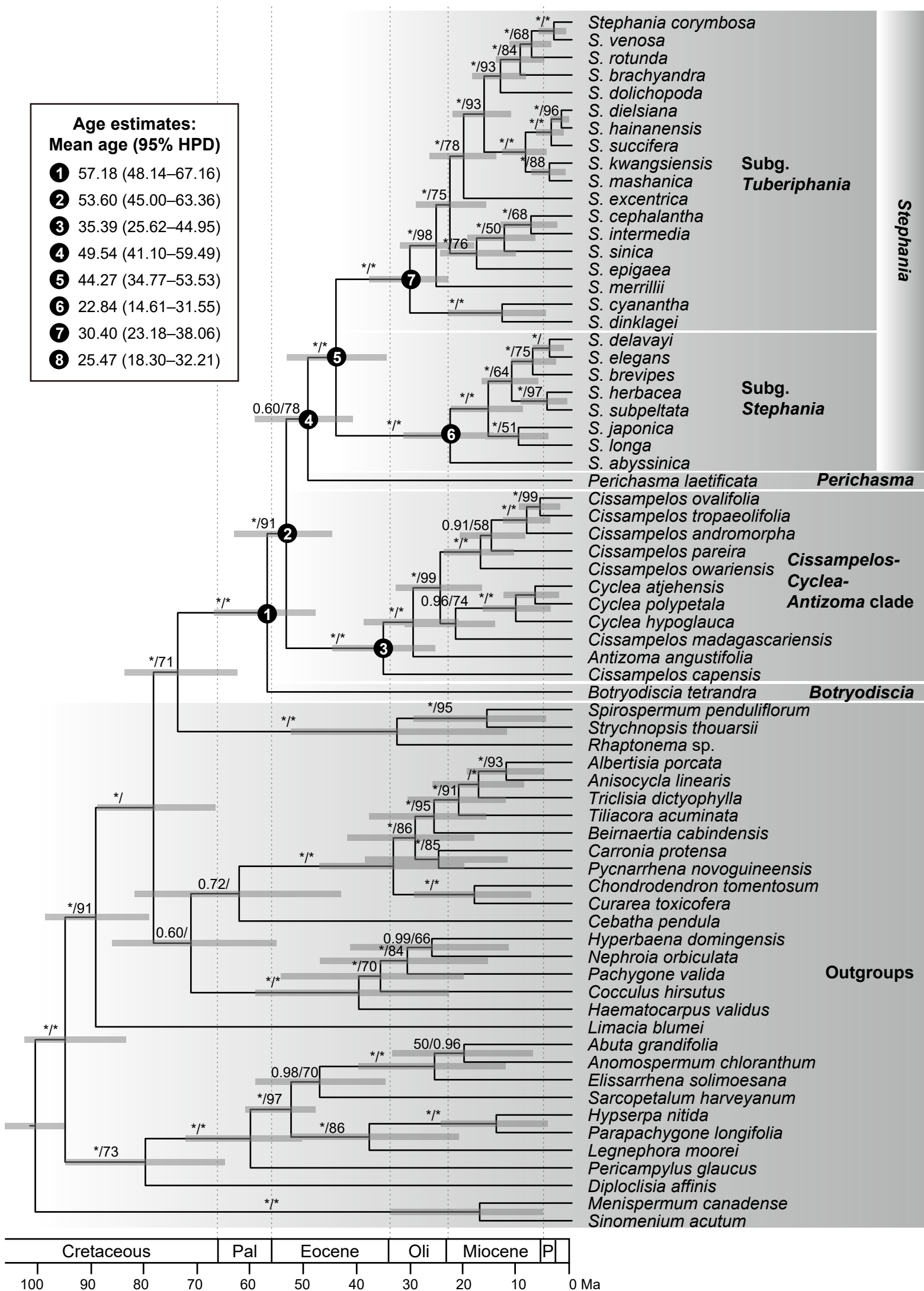

Supplement: mcaf240_Supplementary_Data [file mcaf240_supplementary_data.zip › Lian et al.-Revised Fig. S1.pdf]
