# Supplementary material for: The endocarp evolution of Cissampelideae (Menispermaceae): integrating extant and fossil species
Source: Ann Bot. 2025 Oct 22;137(6):2015–24. doi: 10.1093/aob/mcaf240 (PMC13274978; doi:10.1093/aob/mcaf240)
Supplement: mcaf240_Supplementary_Data [file mcaf240_supplementary_data.zip › Revised Supplementary data.doc]

SUPPLEMENTARY DATA

Figure S1. Chronogram of Cissampelideae inferred from the seven-locus dataset using node-dating in BEAST.

Figure S2. Likelihood inference of evolution of endocarp characters in Cissampelideae with extant and extinct taxa using Mesquite.

Figure S3. Likelihood inference of evolution of endocarp characters in Cissampelideae with extant taxa using Mesquite.

Table S1. Taxa, vouchers and GenBank accession numbers for the sequences used in this study.

Table S2. Endocarp characters and character states used in this study.

Table S3. Data matrix of 24 endocarp characters.

Table S4. Calibrating fossils used for node-dating.

FILE S1. The combined data matrix in nexus format.

**Legend for Figures S1-5**

Figure S1. Chronogram of Cissampelideae inferred from the seven-locus dataset using node-dating in BEAST. Numbers above branches are Bayesian posterior probabilities (PP ≥ 0.60) and bootstrap percentages (BS ≥ 50 %). An asterisk (*) indicates BS = 100% or PP = 1.0. Generic classification is based on Lian *et al*. (2024). Gray bars indicate 95% highest posterior density intervals. Nodes of interests were marked as 1–8. Pal = Palocene; Oli = Oligocene; P = Pliocene.

Figure S2. Likelihood inference of evolution of endocarp characters in Cissampelideae with extant and extinct taxa using Mesquite. (A) Endocarp type; (B) Globose endocarp; (C) Outline endocarp shape; (D) Endocarp length; (E) Endocarp length is much bigger than width (length-width ration >1.5); (F) Endocarp with large central area; (G) Endocarp excavated lateral faces; (H) Perforation present; (I) Condyle parallel to symmetry plane; (J) Chamber; (K) No. dorsal crest; (L) No. lateral crest; (M) Spiny dorsal crest; (N) Spiny lateral crest; (O) A vascular trace near one limb; (P) One limb noticeably longer; (Q) One limb terminating more outwards; (R) Distance between the two limbs; (S) Transversal ridges conspicuous; (T) No. transversal ridges; (U) Broken transversal ridges; (V) Pits between transverse ridges; (W) Transversal ridges lower than dorsal crest; (X) Ornamental type of transversal ridges.

Figure S3. Likelihood inference of evolution of endocarp characters in Cissampelideae with extant taxa using Mesquite. (A) Endocarp type; (B) Globose endocarp; (C) Outline endocarp shape; (D) Endocarp length; (E) Endocarp length is much bigger than width (length-width ration >1.5); (F) Endocarp with large central area; (G) Endocarp excavated lateral faces; (H) Perforation present; (I) Condyle parallel to symmetry plane; (J) Chamber; (K) No. dorsal crest; (L) No. lateral crest; (M) Spiny dorsal crest; (N) Spiny lateral crest; (O) A vascular trace near one limb; (P) One limb noticeably longer; (Q) One limb terminating more outwards; (R) Distance between the two limbs; (S) Transversal ridges conspicuous; (T) No. transversal ridges; (U) Broken transversal ridges; (V) Pits between transverse ridges; (W) Transversal ridges lower than dorsal crest; (X) Ornamental type of transversal ridges.

Table S1. *Taxa, vouchers and GenBank accession numbers for the sequences used in this study. Herbarium acronyms follow Index Herbariorum at <http://sciweb.nybg.org/science2/IndexHerbariorum.asp>*

| Taxon | Voucher | Locality | *rbcL* | *atpB* | *matK* | *ndhF* | *trnL*-*F* | 26S | ITS |
| --- | --- | --- | --- | --- | --- | --- | --- | --- | --- |
| *Antizoma angustifolia* (Burch.) Miers ex Harv. | P.M. Burgoyne & N. Snow 4711 (MO) | South Africa | PP157933 | PP157952 | KX384049 | KX384085 | KX384112 | PP165392 | PP175397 |
| *Botryodiscia tetrandra* (S. Moore) Lian Lian & Wei Wang | He GS 15128 (PE) | Fujian, China | PP157951 | PP157970 | PP157984 | PP157996 | PP158012 | PP165408 | PP175420 |
| *Cissampelos andromorpha* DC. | Ortiz et al. 302 (AMAZ, MO) | Peru | JN051676 | HQ260829 | JN051819 | EF624279 | JN051753 | PP165393 | PP175398 |
| *Cissampelos capensis* L.f. | R.T. Schuh et al. PBI-SA07-L03-H013 (MO) | South Africa | MT153771 | MT153794 | KX384055 | KX384089 | KX384115 | KM364838 | PP175399 |
| *Cissampelos madagascariensis* Miers | P. Antilahimena & V. Razafindraja 8271 (MO) | Madagascar | PP157934 | PP157953 | KX384052 | KX384090 | KX384116 |  | PP175400 |
| *Cissampelos ovalifolia* DC. | Zardini & Chaparro 60300 (MO) | Paraguay | PP157935 | PP157954 | KX384056 | EF624281 | KX384119 |  |  |
| *Cissampelos owariensis* Beauv. ex DC. | JM Acworth 345 (MO) | Central Africa | FJ026473 | FJ026413 |  | EF624282 | PP157997 |  | PP175401 |
| *Cissampelos pareira* L. | Wang HC BN-001 (HIB) | Thailand | AF197590 | FJ026414 | EF143858 | JN051703 | EF143889 | DQ008616 | EF143843 |
| *Cissampelos tropaeolifolia* DC. | Ortiz & al. 229 (MO) | Peru | FJ026475 | FJ026415 | KX384060 | EF624283 | KX384123 |  | PP175402 |
| *Cyclea atjehensis* Forman | Ortiz & Huy 409 (HN, MO) | Vietnam | PP157936 | PP157955 | KX384065 | KX384096 | KX384128 |  |  |
| *Cyclea hypoglauca* (Schauer) Diels | Chen ZD et al. 9812108 (PE) | Guangdong, China | FJ626594 | HQ260833 | EF143862 | JN051707 | EF143894 | FJ626467 | AY017406 |
| *Cyclea polypetala* Dunn | Hong YP 99379 (PE) | Hainan, China | JN051680 | JN051875 | EF143863 | JN051708 | EF143895 |  | AY017407 |
| *Perichasma laetificata* Miers | F Jacques 111 (P) | Cameroon | JN051693 | JN051891 | JN051856 | JN051724 | JN051791 | MT145269 | PP175403 |
| *Stephania abyssinica* (Quart.-Dill. & A.Rich.) Walp. | Chen ZD 2009117 (PE) | Kenya | JN051689 | JN051887 | JN051854 | JN051720 | JN051789 | PP165394 | PP175404 |
| *Stephania brachyandra* Diels | Hong Y-P H043 (PE) | Yunnan, China | JN051690 | JN051888 | EF143871 | JN051721 | EF143903 | MT145271 | AY017401 |
| *Stephania brevipes* Craib | F Jacques 68 (P) | Thailand | PP157937 | PP157956 | PP157971 | PP157985 | PP157998 | PP165395 | PP175405 |
| *Stephania cephalantha* Hayata | Hong Y-P H231 (PE) | Guangxi, China | JN051691 | JN051889 | EF143872 | JN051722 | EF143904 |  | AY017400 |
| *Stephania corymbosa* Walp. | CPG33138 (PE) | Indonesia | PP157938 | PP157957 | PP157972 |  | PP157999 | PP165396 | PP175406 |
| *Stephania cyanantha* Welw. ex Hiern | CPG27415 (PE) | Kenya | PP157939 | PP157958 | PP157973 | PP157986 | PP158000 | PP165397 | PP175407 |
| *Stephania delavayi* Diels | Hong YP et al. H322 (PE) | Guizhou, China | PP157940 | PP157959 | PP157974 | PP157987 | PP158001 | PP165398 | PP175408 |
| *Stephania dielsiana* Y.C. Wu | Hong YP & Shi SH H058 (PE) | Guangdong, China | PP157941 | PP157960 | PP157975 | PP157988 | PP158002 | PP165399 | PP175409 |
| *Stephania dinklagei* (Engl.) Diels | L. Nusbaumer 545 (P) | [Côte d'Ivoire](https://science.mnhn.fr/all/list?country=Côte d'Ivoire) | PX132043 | PX132045 | PX132047 | PX132049 | PX132051 | PX131159 | [PX131161](https://www.ncbi.nlm.nih.gov/nuccore/?term=PX131161:PX131162%5Baccn%5D) |
| *Stephania dolichopoda* Diels | CPG22791 (PE) | Yunnan, China | PP157942 | PP157961 | PP157976 | PP157989 | PP158003 | PP165400 | PP175410 |
| *Stephania elegans* Hook. f. et Thoms. | Hong Y-P 99191 (PE) | Yunnan, China | JN051692 | JN051890 | EF143874 | JN051723 | EF143906 |  | AY017396 |
| *Stephania epigaea* H.S.Lo | Kang & Xie 2012023 (SHMU) | Yunnan, China |  |  |  |  | KJ566166 |  | KJ566135 |
| *Stephania excentrica* H.S.Lo | Kang & Xie 2013015 (SHMU) | Chongqing, China |  |  |  |  | KJ566167 |  | KJ566136 |
| *Stephania hainanensis* Lo et Y. Tsoong | Hong YP 99363 (PE) | Hainan, China | PP157943 | PP157962 | PP157977 | PP157990 | PP158004 | PP165401 | PP175411 |
| *Stephania herbacea* Gagnep. | Kang & Xie 2013013 (SHMU) | Sichuan, China |  |  |  |  | KJ566170 |  | KJ566139 |
| *Stephania intermedia* H.S. Lo | Shui YM 002626 (PE) | Yunnan, China | PP157944 | PP157963 | PP157978 | PP157991 | PP158005 |  |  |
| *Stephania japonica* (Thunb.) Miers | Huang 2054 (HAST) | Taiwan, China | FJ026507 | FJ026447 | JN051855 | EF624335 | JN051790 | MT145270 | PP175412 |
| *Stephania kwangsiensis* H.S. Lo | Qin HN et al. 999 (PE) | Guangxi, China | PP157945 | PP157964 | PP157979 | PP157992 | PP158006 | PP165402 | PP175413 |
| *Stephania longa* Lour. | Hong YP H101 (PE) | Guangxi, China | FJ626473 | AY017399 | EF143907 | EF143875 | JN051725 | HQ260858 | PP175414 |
| *Stephania mashanica* H.S. Lo & B.N. Chang | Anonymous s.n. (PE) | Guangxi, China | PP157946 | PP157965 |  |  | PP158007 | PP165403 | PP175415 |
| *Stephania merrillii* Diels | CPG09665 (PE) | Taiwan, China | PX132044 | PX132046 | PX132048 | PX132050 | PX132052 | PX131160 | PX131162 |
| *Stephania rotunda* Lour. | F Jacques 65 (P) | Thailand | PP157947 | PP157966 | PP157980 | PP157993 | PP158008 | PP165404 | PP175416 |
| *Stephania sinica* Diels | Feng ZB et al. SI0321 (PE) | Sichuan, China | PP157948 | PP157967 | PP157981 | PP157994 | PP158009 | PP165405 | PP175417 |
| *Stephania subpeltata* H.S. Lo | Y.M. Shui et al. 64783 (PE) | Yunnan, China | PP157949 | PP157968 | PP157982 | PP157995 | PP158010 | PP165406 | PP175418 |
| *Stephania succifera* Lo et Y. Tsoong | Hong Y-P 99421 (PE) | Hainan, China | JN051694 | JN051892 | EF143876 | JN051726 | EF143908 |  | AY017403 |
| *Stephania venosa* (Bl.) Spreng. | F Jacques 01 (P) | cult. Bogor | EU526996 | EU526963 | JN051857 | JN051728 | JN051792 | PP165409 | PP175421 |
| **Spirospermeae** |  |  |  |  |  |  |  |  |  |
| *Rhaptonema* sp. | McPherson 18854 (MO) | Madagascar | HQ260796 | HQ260852 | JN051848 | EF624327 | JN051783 | MT145272 | PP175422 |
| *Spirospermum penduliflorum* DC. | McPherson 14176 (MO) | Madagascar | JN051688 | JN051886 | JN051853 | JN051719 | JN051788 |  | PP175423 |
| *Strychnopsis thouarsii* Baill. | Schatz et al. 3728 (MO, P, TEF, WAG) | Madagascar | HQ260803 | HQ260859 | JN051858 | EF624337 | JN051793 | MT145273 | PP175424 |
| **Tiliacoreae** |  |  |  |  |  |  |  |  |  |
| *Albertisia porcata* Breteler (*A*. *papuana* for 26S) | McPherson 16678 (MO) | Gabon | HQ260758 | HQ260814 | KX384047 | EF624261 | KX384110 | KM364835 |  |
| *Anisocycla linearis* Pierre ex Diels | Hong Wa et al. 466 (MO) | Madagascar | HQ260759 | HQ260816 | JN051805 | EF624263 | JN051739 | MW621981 |  |
| *Beirnaertia cabindensis* (Exell & Mendonca) Troupin | Walters & Niangadouma 1267 (MO) | Gabon | HQ260766 | HQ260822 | JN051811 | EF624270 | JN051745 | MW621982 |  |
| *Carronia protensa* (F. Muell.) Diels | van der Werff & Gray 17049 (MO) | Australia | HQ260769 | HQ260825 | JN051815 | EF624274 | JN051749 | MW621983 |  |
| *Chondrodendron tomentosum* Ruiz & Pav. | Ortiz et Vásquez 217 (AMAZ, MO) | Peru | HQ260771 | HQ260771 | JN051818 | EF624278 | JN051752 | KM364844 |  |
| *Curarea toxicofera* (Wedd.) Barneby & Krukoff | Ortiz 184 (AMAZ, MO) | Peru | FJ026480 | FJ026420 | KX384063 | EF624291 | KX384126 | KM364848 |  |
| *Pycnarrhena novoguineensis* Miq. | Gray 8794 (MO) | Australia | HQ260795 | HQ260851 | JN051847 | EF624326 | JN051782 | MW621989 |  |
| *Tiliacora acuminata* (Lam.) Hook. f. & Thoms. | F Jacques 11 (P) | cult. West Java | JN051696 | JN051895 | JN051861 | JN051730 | JN051796 | MW621997 |  |
| *Triclisia dictyophylla* Diels | Kenfack & Zapfack 2038 (MO) | Cameroon | HQ260810 | HQ260866 | JN051866 | EF624344 | JN051801 | MW622000 |  |
| **Cebatheae** |  |  |  |  |  |  |  |  |  |
| *Cebatha pendula* (J.R.Forst. & C.Forst.) Kuntze | DDF 110 (PE) | Kenya | MT153759 | FJ026418 | MT153802 | MT153824 | MT153843 | MT145258 |  |
| **Pachygoneae** |  |  |  |  |  |  |  |  |  |
| *Cocculus hirsutus* (L.) Diels | Chen ZD et al. 21 (PE) | Kenya | MT153748 | MT153772 | MT153795 | MT153813 | MT153832 | MT145246 |  |
| *Nephroia orbiculata* (L.) Lian Lian & Wei Wang | Zhong SW et al. 724 (PE) | Hubei, China | MT153757 | MT153781 | MT153800 | MT153822 | MT153841 | MT145256 |  |
| *Haematocarpus validus* (Miers) Bakh.f. ex Forman | Chase 1321 (K) | cult. West Java | JN051682 | JN051878 | JN051829 | JN051711 | JN051763 | KM364859 |  |
| *Hyperbaena domingensis* (DC.) Benth. | van der Werff et al. 19586 (MO) | Ecuador | HQ260782 | HQ260837 | JN051830 | EF624299 | JN051764 | MT145266 |  |
| *Pachygone valida* Diels | Hong YP 99247 (PE) | Yunnan, China | HQ260792 | HQ260848 | EF143850 | JN051713 | EF143881 |  |  |
| **Limacieae** |  |  |  |  |  |  |  |  |  |
| *Limacia blumei* (Boerl.) Diels | F Jacques 07 (P) | cult. West Java | JN051683 | JN051879 | JN051836 | EF624309 | JN051770 |  |  |
| **Anomospermeae** |  |  |  |  |  |  |  |  |  |
| *Abuta grandifolia* (Mart.) Sandwith | Ortiz 221 (MO) | Peru | FJ026459 | FJ026398 | KX384044 | EF624258 | KX384107 | KM364830 |  |
| *Anomospermum chloranthum* Diels | Ortiz & Aguilar 324 (INB, MO) | Costa Rica | HQ260760 | JN051869 | JN051806 | EF624265 | JN051740 | MK288755 |  |
| *Elissarrhena solimoesana* (Moldenke) Wei Wang & R. Ortiz. | Ortiz & Vargas 198 (MO, QCNE) |  | HQ260762 | HQ260819 | JN051809 | EF624268 | JN051743 | MK288758 |  |
| *Diploclisia affinis* (Oliv.) Diels (*D*. *glaucescens* for 26S) | Hong YP H149 (PE) | Guangxi, China | JN051681 | JN051876 | EF143866 | JN051709 | EF143898 | KM364853 |  |
| *Hypserpa nitida* Miers ex Benth. (*H. decumbens* for 26S) | Hong YP 99378 (PE) |  | FJ626596 | HQ260839 | EF143868 | JN051712 | EF143900 | KM364863 |  |
| *Parapachygone longifolia* Forman | Gray 9245 (QRS) | Australia | JN051686 | JN051883 | JN051843 | JN051715 | JN051778 | KM36487 |  |
| *Pericampylus glaucus* (Lam.) Merr. | Chen ZD et al. 9812095 (PE) | Guangdong, China | FJ626598 | JN051884 | EF143869 | EF624324 | EF143901 | FJ626471 |  |
| *Legnephora moorei* (F. Muell.) Miers | van der Werff & Gray 17053 (MO) | Australia | HQ260787 | HQ260842 | JN051834 | EF624307 | JN051768 | MT145274 |  |
| *Sarcopetalum harveyanum* F.Muell. | van der Werff 17058 (MO) | Australia | HQ260798 | HQ260854 | JN051850 | EF624329 | JN051785 | MT145275 |  |
| **Menispermeae** |  |  |  |  |  |  |  |  |  |
| *Menispermum canadense* L. | Ortiz et al. 235 (MO) | USA | AF190437 | AF093384 | GU266604 | EF624311 | JN051771 | KM364867 |  |
| *Sinomenium acutum* (Thunb.) Rehder & E.H.Wilson | Hong YP H006 (PE) | Henan, China | FJ626600 | JN051718 | EF143870 | JN051718 | EF143902 | FJ626472 |  |

Table S2. *Endocarp characters and character states used in this study. Morphological matrix of extant and fossil Cissampelideae, modified from combined matrix of Herrera (2011), Jacques et al. (2011), Wefferling et al. (2013), Ortiz et al. (2016) and Lian et al. (2024).*

| No. | Character | Character state |
| --- | --- | --- |
| 1 | Endocarp type | 0, horseshoe-shaped; 1, cochleate; 2, spiral |
| 2 | Globose endocarp | 0, yes; 1, no |
| 3 | Outline endocarp shape | 0, obovate; 1, elliptic; 2, rounded; 3, comma-shaped |
| 4 | Endocarp length (mm) | 0, small (<5 mm); 1, medium (5-10 mm); 2, large (>10 mm) |
| 5 | Endocarp length is much bigger than width (length-width ration >1.5) | 0, yes; 1, no |
| 6 | Endocarp with large central area | 0, yes; 1, no |
| 7 | Endocarp excavated lateral faces | 0, yes; 1, no |
| 8 | Perforation present | 0, yes; 1, no |
| 9 | Condyle parallel to symmetry plane | 0, yes; 1, no |
| 10 | Chamber | 0, present; 1, absent |
| 11 | No. dorsal crest | 0, 0; 1, 1; 2, 2 |
| 12 | No. lateral crest | 0, 0; 1, 1; 2, 2 |
| 13 | Spiny dorsal crest | 0, yes; 1, no |
| 14 | Spiny lateral crest | 0, yes; 1, no |
| 15 | A vascular trace near one limb | 0, yes; 1, no |
| 16 | One limb noticeably longer | 0, yes; 1, no |
| 17 | One limb terminating more outwards | 0, yes; 1, no |
| 18 | Distance between the two limbs | 0, obvious; 1, lacking |
| 19 | Transversal ridges conspicuous | 0, yes; 1, no |
| 20 | No. transversal ridges | 0, 1-10; 1, 11-20; 2, >20 |
| 21 | Broken transversal ridges | 0, yes; 1, no |
| 22 | Pits between transverse ridges | 0, present; 1, absent |
| 23 | Transversal ridges lower than dorsal crest | 0, yes; 1, no |
| 24 | Ornamental type of transversal ridges | 0, smooth transversal ridges without pits; 1, smooth transversal ridges with pits; 2, strong spine; 3, transversal and reticulated ridges; 4, short raised transversal ridges; 5, irregular tubercles; 6, long raised transversal ridges; 7, broad fold-like transversal ridges; 8, columnar hooked protuberances; 9, longitudinal strips |

*Table S3. Data matrix of 24 endocarp characters. Taxa with “^†^” are fossil species. Inapplicable states are coded with a dash “–”; “?”= missing data.*

|  | Character number | | | | | | | | | | | | | | | | | | | | | | | |
| --- | --- | --- | --- | --- | --- | --- | --- | --- | --- | --- | --- | --- | --- | --- | --- | --- | --- | --- | --- | --- | --- | --- | --- | --- |
| Taxa | 1 | 2 | 3 | 4 | 5 | 6 | 7 | 8 | 9 | 10 | 11 | 12 | 13 | 14 | 15 | 16 | 17 | 18 | 19 | 20 | 21 | 22 | 23 | 24 |
| *Abuta grandifolia* | 0 | 0 | 1 | 2 | 0/1 | 1 | 1 | 1 | 1 | 1 | 0 | 0 | 1 | 1 | 1 | 1 | 1 | 1 | 1 | – | – | – | – | – |
| *Albertisia porcata* | 0 | 0 | 1 | 2 | 0 | 1 | 1 | 1 | 1 | 1 | 0 | 0 | 1 | 1 | 1 | 1 | 1 | 1 | 1 | – | – | – | – | – |
| *Anisocycla linearis* | 0 | 0 | 1 | 2 | 0/1 | 1 | 1 | 1 | 1 | 1 | 0 | 0 | 1 | 1 | 1 | 1 | 1 | 1 | 1 | – | – | – | – | – |
| *Anomospermum chloranthum* | 0 | 0 | 1 | 2 | 1 | 1 | 1 | 1 | 1 | 1 | 0 | 0 | 1 | 1 | 1 | 1 | 1 | 1 | 1 | – | – | – | – | – |
| *Antizoma angustifolia* | ? | ? | ? | ? | ? | ? | ? | ? | ? | ? | ? | ? | ? | ? | ? | ? | ? | ? | ? | ? | ? | ? | ? | ? |
| *Beirnaertia cabindensis* | 0 | 0 | ? | ? | ? | 1 | 1 | 1 | 1 | 1 | 0 | 0 | 1 | 1 | 1 | 1 | 1 | 1 | 1 | – | – | – | – | – |
| *Carronia protensa* | 0 | 0 | 0 | 2 | 0 | 1 | 1 | 1 | 1 | 1 | 1 | 0 | 1 | 1 | 1 | 1 | 0 | 1 | 1 | – | – | – | – | – |
| *Cebatha pendula* | 1 | 1 | 3 | 0 | 1 | 1 | 0 | 1 | 0 | 1 | 1 | 1 | 1 | 1 | 1 | 1 | 1 | 1 | 1 | – | – | – | – | – |
| *Chondrodendron tomentosum* | 0 | 0 | 1 | 2 | 1 | 1 | 1 | 1 | 1 | 1 | 0 | 0 | 1 | 1 | 1 | 1 | 1 | 1 | 1 | – | – | – | – | – |
| *Cissampelos andromorpha* | 0 | 1 | 0 | 0 | 1 | 0 | 0 | 1 | 0 | 0 | 1 | 2 | 0 | 0 | 1 | 1 | 1 | 0 | 0 | 1 | 0 | 1 | 1 | 5 |
| *Cissampelos capensis* | 0 | 1 | 0 | 1 | 1 | 0 | 0 | 1 | 0 | 0 | 1 | 2 | 0 | 0 | 1 | 0 | 1 | 0 | 0 | 2 | 0 | 1 | 0 | 5 |
| *Cissampelos defranceschii*^†^ | 0 | 1 | 0 | 0 | 1 | 0 | 0 | 1 | 0 | 0 | 1 | 2 | 0 | 0 | 1 | 0 | 1 | 0 | 0 | 1 | ? | 1 | 0 | 5 |
| *Cissampelos madagascariensis* | ? | ? | ? | ? | ? | ? | ? | ? | ? | ? | ? | ? | ? | ? | ? | ? | ? | ? | ? | ? | ? | ? | ? | ? |
| *Cissampelos ovalifolia* | 0 | 1 | 0 | 0 | 1 | 0 | 0 | 0 | 0 | 0 | 1 | 2 | 0 | 0 | 1 | 0 | 1 | 0 | 0 | 1 | 0 | 1 | 1 | 5 |
| *Cissampelos owariensis* | 0 | 1 | 0 | 0 | 1 | 0 | 0 | 1 | 0 | 0 | 1 | 2 | 0 | 0 | 1 | 0 | 0 | 0 | 0 | 2 | 0 | 1 | 1 | 5 |
| *Cissampelos pareira* | 0 | 1 | 0 | 0 | 1 | 0 | 0 | 0 | 0 | 0 | 1 | 2 | 0 | 0 | 1 | 0 | 1 | 0 | 0 | 1/2 | 0 | 1 | 1 | 5 |
| *Cissampelos rusingensis*^†^ | 0 | 1 | 0 | 1 | 1 | 0 | 0 | 1 | 0 | 0 | 1 | 2 | 0 | 0 | 1 | 0 | 1 | 0 | 0 | ? | ? | 1 | 0 | ? |
| *Cissampelos tropaeolifolia* | 0 | 1 | 0 | 0 | 1 | 0 | 0 | 1 | 0 | 0 | 1 | 2 | 0 | 0 | 1 | 1 | 1 | 0 | 0 | 1 | 0 | 1 | 1 | 5 |
| *Cocculus hirsutus* | 1 | 1 | 3 | 0 | 1 | 1 | 0 | 0 | 0 | 1 | 1 | 1 | 1 | 1 | 1 | 1 | 1 | 1 | 0 | 1 | 1 | 1 | 0 | 3 |
| *Curarea toxicofera* | 0 | 0 | ? | ? | ? | 1 | 1 | 1 | 1 | 1 | 0 | 0 | 1 | 1 | 1 | 1 | 1 | 1 | 1 | – | – | – | – | – |
| *Cyclea atjehensis* | 0 | 1 | 0 | 1 | 1 | 0 | 0 | 1 | 0 | 0 | 1 | 2 | 0 | 0 | 1 | 1 | 1 | 0 | 0 | 1 | 0 | 1 | 1 | 5 |
| *Cyclea hypoglauca* | 0 | 1 | 0 | 0 | 1 | 0 | 0 | 1 | 0 | 0 | 1 | 2 | 0 | 0 | 1 | 1 | 1 | 0 | 0 | 1/2 | 0 | 1 | 1 | 5 |
| *Cyclea polypetala* | 0 | 1 | 0 | 0 | 1 | 0 | 0 | 0 | 0 | 0 | 1 | 2 | 0 | 0 | 1 | 1 | 1 | 0 | 0 | 1 | 0 | 1 | 1 | 5 |
| *Diploclisia affinis* | 0 | 1 | 0 | 0/2 | 0/1 | 0 | 0 | 1 | 0/1 | 1 | 1 | 1 | 1 | 1 | 1 | 1 | 0/1 | 0/1 | 0 | 2 | 1 | 0 | 0 | 1 |
| *Elissarrhena solimoesana* | 0 | 0 | 1 | 2 | 0 | 1 | 1 | 1 | 1 | 1 | 0 | 0 | 1 | 1 | 1 | 1 | 1 | 1 | 1 | – | – | – | – | – |
| *Haematocarpus validus* | ? | ? | ? | ? | ? | ? | ? | ? | ? | ? | ? | ? | ? | ? | ? | ? | ? | ? | ? | ? | ? | ? | ? | ? |
| *Hyperbaena domingensis* | 1 | 1 | 3 | 2 | 1 | 1 | 1 | 1 | 1 | 1 | 1 | 0 | 1 | 1 | 1 | 1 | 1 | 1 | 1 | – | – | – | – | – |
| *Hypserpa nitida* | 0 | 0 | 2 | 1 | 1 | 1 | 1 | 1 | 0 | 1 | 0 | 0 | 1 | 1 | 1 | 1 | 1 | 1 | 1 | – | – | – | – | – |
| *Legnephora moorei* | 0 | 1 | 2 | 2 | 1 | 0 | 1 | 1 | 0 | 1 | 1 | 0 | 1 | 1 | 1 | 1 | 1 | 0 | 1 | – | – | – | – | – |
| *Limacia blumei* | 0 | 1 | 0 | 2 | 1 | 0 | 1 | 1 | 0 | 1 | 1 | 0 | 1 | 1 | 1 | 1 | 1 | 1 | 1 | – | – | – | – | – |
| *Menispermum canadense* | 0 | 1 | 2 | 1 | 1 | 0 | 0 | 1 | 0 | 1 | 1 | 1 | 0/1 | 0/1 | 0 | 1 | 1 | 0 | 0/1 | 2 | 1 | 1 | 0 | 0 |
| *Nephroia orbiculata* | 1 | 1 | 3 | 1 | 1 | 1 | 0 | 1 | 0 | 1 | 1 | 1 | 1 | 1 | 1 | 1 | 1 | 1 | 0 | 1 | 1 | 1 | 0 | 3 |
| *Pachygone valida* | 1 | 1 | 3 | 1 | 1 | 1 | 0 | 0/1 | 0 | 1 | 0 | 0 | 1 | 1 | 1 | 1 | 1 | 1 | 1 | – | – | – | – | – |
| *Palaeoluna bogotensis*^†^ | 0 | 1 | 0 | 1 | 1 | 0 | 0 | 1 | 0 | 1 | 1 | 1 | 1 | 0 | 1 | 0 | 1 | 0 | 0 | 1 | ? | 1 | 0 | 4 |
| *Parapachygone longifolia* | 0 | 0 | 2 | ? | 1 | 1 | 1 | 1 | 0 | 1 | 0 | 0 | 1 | 1 | 1 | ? | 1 | 0 | 1 | – | – | – | – | – |
| *Pericampylus glaucus* | 0 | 1 | 2 | 0/1 | 1 | 0 | 0 | 1 | 0 | 1 | 2 | 2 | 0 | 0 | 1 | 1 | 1 | 0 | 0 | 1 | 1 | 1 | 1 | 2 |
| *Perichasma laetificata* | 0 | 1 | 0 | 2 | 1 | 0 | 0 | 1 | 0 | 1 | 1 | – | 0 | – | 1 | 0 | 1 | 0 | 0 | 1 | – | – | – | 9 |
| *Pycnarrhena novoguineensis* | 0 | 0 | 1 | 1/2 | 1 | 1 | 1 | 1 | 1 | 1 | 0 | 0 | 1 | 1 | 1 | 1 | 1 | 1 | 1 | – | – | – | – | – |
| *Rhaptonema* sp. | 2 | 1 | 2 | 2 | 1 | 1 | 1 | 1 | 0 | 1 | 1 | 0 | 1 | 1 | 1 | – | – | – | 1 | – | – | – | – | – |
| *Sarcopetalum harveyanum* | 0 | 1 | 2 | 1 | 1 | 0 | 0 | 1 | 0 | 1 | 2 | 2 | 0 | 0 | 1 | 1 | 1 | 0 | 0 | 1 | 1 | 1 | 1 | 2 |
| *Sinomenium acutum* | 0 | 1 | 2 | 1 | 1 | 0 | 0 | 1 | 0 | 1 | 1 | 1 | 0 | 0 | 0 | 1 | 1 | 0 | 0 | 2 | 1 | 1 | 0 | 0 |
| *Spirospermum penduliflorum* | 2 | 1 | 2 | 2 | 1 | 1 | 1 | 1 | 0 | 1 | 1 | 0 | 1 | 1 | 1 | – | – | – | 1 | – | – | – | – | – |
| *Stephania abyssinica* | 0 | 1 | 0 | 1 | 1 | 0 | 0 | 1 | 0 | 1 | 1 | 1 | 0 | 0 | 1 | 0 | 1 | 0 | 0 | 0 | 0 | 1 | 1 | 6 |
| *Stephania auriformis*^†^ | 0 | 1 | 0 | 1 | 0 | 0 | 0 | 0 | 0 | 1 | 1 | 1 | 1 | 1 | 1 | 1 | 0 | 1 | 0 | 2 | 1 | 0 | 0 | 1 |
| *Stephania bangorensis*^†^ | 0 | 1 | 0 | 1 | 1 | 0 | 0 | ? | 0 | 1 | 1 | 1 | 1 | 0 | 1 | 1 | 1 | 0 | 0 | 1 | 1 | 1 | 0 | 4 |
| *Stephania brachyandra* | 0 | 1 | 0 | 1 | 1 | 0 | 0 | 0 | 0 | 1 | 1 | 1 | 0 | 0 | 1 | 1 | 1 | 0 | 0 | 1 | 0 | 1 | 1 | 8 |
| *Stephania brevipes* | 0 | 1 | 0 | 1 | 1 | 0 | 0 | 0 | 0 | 1 | 1 | 1 | 0 | 0 | 1 | 1 | 1 | 0 | 0 | 0 | 0 | 1 | 1 | 6 |
| *Stephania cephalantha* | 0 | 1 | 0 | 0/1 | 1 | 0 | 0 | 0/1 | 0 | 1 | 1 | 1 | 0 | 0 | 1 | 1 | 1 | 0 | 0 | 1 | 0 | 1 | 1 | 7 |
| *Stephania corymbosa* | 0 | 1 | 0 | ? | 1 | 0 | 0 | 0 | 0 | 1 | 1 | 1 | 0 | 0 | 1 | 1 | 1 | 0 | 0 | 1 | 0 | 1 | 1 | 8 |
| *Stephania cyanantha* | 0 | 1 | 0 | 0/1 | 1 | 0 | 0 | 1 | 0 | 1 | 1 | 1 | 0 | 0 | 1 | 0 | 1 | 0 | 0 | 1 | 0 | 1 | 1 | 7 |
| *Stephania delavayi* | 0 | 1 | 0 | 0 | 1 | 0 | 0 | 0 | 0 | 1 | 1 | 1 | 0 | 0 | 1 | ? | 1 | 0 | 0 | 0 | 0 | 1 | 1 | 6 |
| *Stephania dielsiana* | 0 | 1 | 0 | 1 | 1 | 0 | 0 | 0 | 0 | 1 | 1 | 1 | 0 | 0 | 1 | 1 | 1 | 0 | 0 | 1 | 0 | 1 | 1 | 8 |
| *Stephania dolichopoda* | 0 | 1 | 0 | 1 | 1 | 0 | 0 | 0 | 0 | 1 | 1 | 1 | 0 | 0 | 1 | 1 | 1 | 0 | 0 | 1 | 0 | 1 | 1 | 8 |
| *Stephania elegans* | 0 | 1 | 0 | 1 | 1 | 0 | 0 | 1 | 0 | 1 | 1 | 1 | 0 | 0 | 1 | ? | 1 | 0 | 0 | 0 | 0 | 1 | 1 | 6 |
| *Stephania epigaea* | 0 | 1 | 0 | 1 | 1 | 0 | 0 | 1 | 0 | 1 | 1 | 1 | 0 | 0 | 1 | 1 | 1 | 0 | 0 | 1 | 0 | 1 | 1 | 7 |
| *Stephania excentrica* | 0 | 1 | 0 | 1 | 1 | 0 | 0 | 0 | 0 | 1 | 1 | 1 | 0 | 0 | 1 | 1 | 1 | 0 | 0 | 1 | 0 | 1 | 1 | 8 |
| *Stephania geniculata*^†^ | 0 | 1 | 0 | 0 | 1 | 0 | 0 | ? | 0 | 1 | 1 | 1 | 1 | 0 | 1 | 0 | 1 | 0 | 0 | 1 | 1 | 1 | 0 | 4 |
| *Stephania hainanensis* | 0 | 1 | 0 | 1/2 | 1 | 0 | 0 | 0 | 0 | 1 | 1 | 1 | 0 | 0 | 1 | 1 | 1 | 0 | 0 | 1 | 0 | 1 | 1 | 8 |
| *Stephania herbacea* | 0 | 1 | 0 | 1 | 1 | 0 | 0 | 1 | 0 | 1 | 1 | 1 | 0 | 0 | 1 | 1 | 1 | 0 | 0 | 0 | 0 | 1 | 1 | 6 |
| *Stephania hootae*^†^ | 0 | 1 | 0 | 1 | 1 | 0 | 0 | 0 | 0 | 0 | 1 | 2 | 0 | 0 | 1 | 0 | 1 | 0 | 0 | 2 | 0 | 1 | 1 | 5 |
| *Stephania intermedia* | 0 | 1 | 0 | 1 | 1 | 0 | 0 | 0/1 | 0 | 1 | 1 | 1 | 0 | 0 | 1 | 1 | 1 | 0 | 0 | 1 | 0 | 1 | 1 | 7 |
| *Stephania jacquesii*^†^ | 0 | 1 | 0 | 1 | 1 | 0 | 0 | 0 | 0 | 1 | 1 | 1 | 1 | 1 | 1 | 1 | 0 | 0 | 0 | 2 | 1 | 0 | 0 | 1 |
| *Stephania japonica* | 0 | 1 | 0 | 1 | 1 | 0 | 0 | 0/1 | 0 | 1 | 1 | 1 | 0 | 0 | 1 | 1 | 1 | 0 | 0 | 0 | 0 | 1 | 1 | 6 |
| *Stephania kwangsiensis* | 0 | 1 | 0 | 1 | 1 | 0 | 0 | 0 | 0 | 1 | 1 | 1 | 0 | 0 | 1 | 1 | 1 | 0 | 0 | 1 | 0 | 1 | 1 | 8 |
| *Stephania longa* | 0 | 1 | 0 | 1 | 1 | 0 | 0 | 0/1 | 0 | 1 | 1 | 1 | 0 | 0 | 1 | 1 | 1 | 0 | 0 | 0 | 0 | 1 | 1 | 6 |
| *Stephania mashanica* | 0 | 1 | 0 | 1 | 1 | 0 | 0 | 0 | 0 | 1 | 1 | 1 | 0 | 0 | 1 | 1 | 1 | 0 | 0 | 1 | 0 | 1 | 1 | 8 |
| *Stephania miocenica*^†^ | 0 | 1 | 0 | 0 | 1 | 0 | 0 | 1 | 0 | 1 | 1 | 1 | 0 | 0 | 1 | 0 | 1 | 0 | 0 | 0 | ? | 1 | 1 | 6 |
| *Stephania ornamenta*^†^ | 0 | 1 | 0 | 0 | 1 | 0 | 0 | 0 | 0 | 1 | 1 | 1 | 1 | 1 | 1 | 1 | 1 | 0 | 0 | 2 | 1 | 0 | 0 | 1 |
| *Stephania palaeosudamericana*^†^ | 0 | 1 | 0 | 0/1 | 1 | 0 | 0 | ? | 0 | 1 | 1 | 1 | 1 | 0 | 1 | 0 | 1 | 0 | 0 | 1 | ? | 1 | 0 | 4 |
| *Stephania psittaca*^†^ | 0 | 1 | 0 | 0 | 1 | 0 | 0 | 1 | 0 | 1 | 1 | 1 | 1 | 0 | 1 | 0 | 1 | 0 | 0 | 1 | 1 | 1 | 0 | 4 |
| *Stephania rotunda* | 0 | 1 | 0 | 1 | 1 | 0 | 0 | 0 | 0 | 1 | 1 | 1 | 0 | 0 | 1 | 1 | 0 | 0 | 0 | 1 | 0 | 1 | 1 | 8 |
| *Stephania shuangxingii*^†^ | 0 | 1 | 0 | 0/1 | 0/1 | 0 | 0 | ? | 0 | 1 | 1 | 1 | 1 | 0 | 1 | 0 | 1 | 0 | 0 | 1 | 1 | 1 | 0 | 4 |
| *Stephania sinica* | 0 | 1 | 0 | 1 | 1 | 0 | 0 | 1 | 0 | 1 | 1 | 1 | 0 | 0 | 1 | 0 | 1 | 0 | 0 | 1 | 0 | 1 | 1 | 7 |
| *Stephania subpeltata* | 0 | 1 | 0 | 0 | 1 | 0 | 0 | 1 | 0 | 1 | 1 | 1 | 0 | 0 | 1 | 1 | 1 | 0 | 0 | 0 | 0 | 1 | 1 | 6 |
| *Stephania succifera* | 0 | 1 | 0 | 1 | 1 | 0 | 0 | 0 | 0 | 1 | 1 | 1 | 0 | 0 | 1 | 1 | 1 | 0 | 0 | 1 | 0 | 1 | 1 | 8 |
| *Stephania tetrandra* | 0 | 1 | 0 | 0/1 | 1 | 0 | 0 | 1 | 0 | 1 | 1 | 1 | 0 | 0 | 1 | 0 | 1 | 0 | 0 | 1 | 0 | 1 | 1 | 4 |
| *Stephania venosa* | 0 | 1 | 0 | 1 | 1 | 0 | 0 | 0 | 0 | 1 | 1 | 1 | 0 | 0 | 1 | 1 | 1 | 0 | 0 | 1 | 0 | 1 | 1 | 8 |
| *Stephania wilfii*^†^ | 0 | 1 | 0 | 0/1 | 1 | 0 | 0 | ? | 0 | 1 | 1 | 1 | 1 | 0 | 1 | 1 | 1 | 0 | 0 | 1 | 1 | 1 | 0 | 4 |
| *Strychnopsis thouarsii* | 2 | 1 | 2 | 2 | 1 | 1 | 1 | 1 | 0 | 1 | 1 | 0 | 1 | 1 | 1 | – | – | – | 1 | – | – | – | – | – |
| *Tiliacora acuminata* | 0 | 0 | 0/1 | 1/2 | 1 | 1 | 1 | 1 | 1 | 1 | 0/1 | 0 | 1 | 1 | 1 | 1 | 0 | 1 | 1 | – | – | – | – | – |
| *Triclisia dictyophylla* | 0 | 0 | 1 | 2 | 1 | 1 | 1 | 1 | 1 | 1 | 0 | 0 | 1 | 1 | 1 | 1 | 0 | 1 | 1 | – | – | – | – | – |

Table S4. Calibrating fossils used for node-dating.

| Node | Fossil | Extant taxon | Age (Ma) | Locality | Justification |
| --- | --- | --- | --- | --- | --- |
| 1 | *Stephania ornamenta* | *Diploclisia* | 59.24 | Guangdong, China | The fossil *Stephania ornamenta* (Han *et al*., 2020) united with extant *Diploclisia* based on phylogenetic analysis (Lian *et al*., 2024). Thus, we used this fossil to constrain the stem node of *Diploclisia*. |
| 2 | *Eohypserpa* sp. | *Hypserpa*, *Parapachygone* and *Legnephora* | 48.07 | Le Quesnoy, France | The fossil *Eohypserpa* sp. (Jacques and De Franceschi, 2005) united with extant *Hypserpa*, *Parapachygone* and *Legnephora* based on phylogenetic analysis (Jacques *et al*., 2011). Thus, we used this fossil to constrain the stem node of the clade containing *Hypserpa*, *Parapachygone*, and *Legnephora*. |
| 3 | *Sarcopetalum jackieae* | *Sarcopetalum* | 23.04 | Queensland, Australia | The fossil *Sarcopetalum jackieae* is assignable to extant monotypic *Sarcopetalum* according to the flattened condylar lateral face, semicircular outline of the endocarp, and alternating spines on spinulose ornamentation on lateral faces (Rozefelds, 1991). |
| 4 | *Davisicarpum gibbosum* | *Limacia* | 48.07 | London clay, UK | Based on the inflated median area of endocarps partly surrounded by a flattened ribbon-like locular rim, the fossil *Davisicarpum* is allied to extant *Limacia* (Chandler, 1978; Manchester, 1994). |
| 5 | *Triclisia inflata* | *Triclisia* | 15.98 | Rusinga island, Kenya | Based on the size, shape and ornamentation of endocarps, the fossil *Triclisia inflata* is assignable to extant *Triclisia* (Chesters, 1957). |
| 6 | *Stephania psittaca* | Cissampelideae | 61.66 | Chubut, Argentina | The fossil *Stephania psittaca* (Jud *et al*., 2018) is resolved as the stem group members of Cissampelideae based on phylogenetic analysis (Lian *et al*., 2024). Thus, we used this fossil to constrain the stem node of Cissampelideae. |
| 7 | *Stephania hootae* | *Cissampelos*, *Cyclea* and *Antizoma* | 37.71 | Hessen, Germany | The fossil *Stephania hootae* (Collinson *et al*., 2012) united with extant *Cissampelos*, *Cyclea* and *Antizoma* based on phylogenetic analysis (Lian *et al*., 2024). Thus, we used this fossil to constrain the stem node of the clade containing *Cissampelos*, *Cyclea* and *Antizoma*. |
| 8 | *Stephania miocenica* | *Stephania* subg. *Stephania* | 15.98 | Nyanza, Kenya | The fossil *Stephania miocenica* united with extant *Stephania* subg. *Stephania* based on phylogenetic analysis (Lian *et al*., 2024). Thus, we used this fossil to constrain the stem node of the *Stephania* subg. *Stephania*. |
